# Constant pH Simulation with FMM Electrostatics in GROMACS. (A) Design and Applications

**DOI:** 10.1021/acs.jctc.4c01318

**Published:** 2025-02-07

**Authors:** Eliane Briand, Bartosz Kohnke, Carsten Kutzner, Helmut Grubmüller

**Affiliations:** Theoretical and Computational Biophysics, Max Planck Institute for Multidisciplinary Sciences, Am Fassberg 11, 37077 Göttingen, Germany

## Abstract

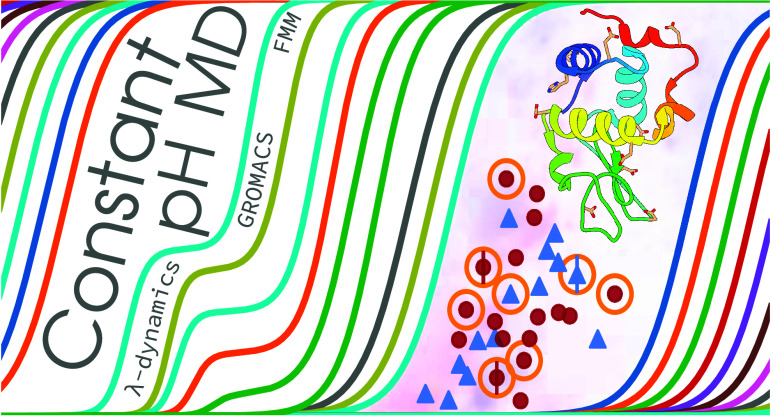

The structural dynamics of biological macromolecules, such as proteins, DNA/RNA, or complexes thereof, are strongly influenced by protonation changes of their typically many titratable groups, which explains their sensitivity to pH changes. Conversely, conformational and environmental changes of the biomolecule affect the protonation state of these groups. With few exceptions, conventional force field-based molecular dynamics (MD) simulations neither account for these effects nor do they allow for coupling to a pH buffer. Here, we present design decisions and applications of a rigorous Hamiltonian interpolation λ-dynamics constant pH method in GROMACS, which rests on GPU-accelerated Fast Multipole Method (FMM) electrostatics. Our implementation supports both CHARMM36m and Amber99sb*-ILDN force fields and is largely automated to enable seamless switching from regular MD to constant pH MD, involving minimal changes to the input files. Here, the first of two companion papers describes the underlying constant pH protocol and sample applications to several prototypical benchmark systems such as cardiotoxin V, lysozyme, and staphylococcal nuclease. Enhanced convergence is achieved through a new dynamic barrier height optimization method, and high p*K*_a_ accuracy is demonstrated. We use Functional Mode Analysis (FMA) and Mutual Information (MI) to explore the complex intra- and intermolecular couplings between the protonation states of titratable groups as well as those between protonation states and conformational dynamics. We identify striking conformation-dependent p*K*_a_ variations and unexpected inter-residue couplings. Conformation–protonation coupling is identified as a primary cause of the slow protonation convergence notorious to constant pH simulations involving multiple titratable groups, suggesting enhanced sampling methods to accelerate convergence.

## 1 Introduction

The activity of hydrogen ions (pH) is one of the most important solution conditions for biomolecular systems, alongside temperature and ionic strength. This importance stems from its direct influence on titratable residues, like Histidine (His), Aspartic acid (Asp), or Glutamic Acid (Glu), which switch between protonated and deprotonated forms. Alongside pH, other factors contribute to residue protonation: first, the intrinsic p*K*_a_ of the moiety (i.e., the free energy of deprotonation linked to the chemical bond breaking); second, and most relevantly in proteins, the local electrostatic and steric environment, which is time- and conformation-dependent. In turn, the charge change upon (de)protonation influences the dynamics of the protein through electrostatic interactions, ultimately affecting the conformations adopted. The close interplay between protonation state and conformational ensemble is relevant for many biological phenomena such as (i) the stabilization of protein structure, either through polarization effects or direct salt bridge interactions,^1^ (ii) protein folding,^2^ or (iii) the catalytic activities of enzymes.

However, most molecular dynamics (MD) simulations today use fixed protonation states, which cannot capture the aforementioned interplay between conformation and protonation, and also do not allow for simulations at specific pH values. To address these limitations, constant pH simulations (CPH-MD) have been developed^3−21^ which enable dynamic protonation changes in response to pH and the electrostatic environment of titratable groups throughout the simulation. The development of such CPH-MD methods faces various challenges, which we summarize below to motivate and explain our solutions, as implemented in our GROMACS-based CPH-MD code.

Ideally, describing protonation in MD simulations by explicit hydronium ions and reactive titratable groups would yield a constant pH method which accurately reproduces both thermodynamic and kinetic properties. For the aim of obtaining an accurate conformational and titration ensemble, e.g., for calculating titration curves, this approach is inefficient for several reasons. First, protonation changes within the protein core can be as slow as tens of microseconds.^22^ Second, the diffusion of explicit hydronium ions between protonatable residues adds another hurdle for a protonation event to occur. Finally, physiological pH cannot be easily realized in typically sized simulation boxes with explicit, integer-charged hydronium ions.^14^ In the context of MD, methods to overcome these impediments are required.

Indeed, a variety of constant pH methods have been developed that introduce virtual titration coordinates instead of explicit, diffusing protons,^23,24^ see de Oliveira et al.^25^ for a concise review. They can be broadly categorized by (a) how they treat the solvent — implicitly^3−9^ versus explicitly^10−21^—and (b) by the type of the titration coordinate — discrete (e.g., refs (5,6,8,12,13,19,26−28)) versus continuous (e.g., refs (3,4,7,9−11,14−16,18,20,21,29−31)).

Discrete titration coordinate methods employ Monte Carlo (MC) steps at regular intervals to switch between two states for each titratable residue: protonated and deprotonated. As the energy difference between these states in explicit solvent tends to be quite large due to the water orientation effect, earlier MC protocols used an implicit solvent auxiliary simulation based on Generalized Born^6,8,27^ or Poisson–Boltzmann electrostatics.^5,13,26^ Recent MC protocols have partially mitigated this limitation by substituting implicit solvent evaluations for brief nonequilibrium MD simulations^19,32,33^ or thermodynamic integration,^12,34^ as well as efficiently choosing candidate residues for protonation state change,^19^ showing that discrete coordinate methods are still under active development.

Continuous titration methods^4,7,35^ use a continuous titration coordinate λ to interpolate between two Hamiltonians representing a protonated and a deprotonated state, respectively,^36^ which avoids the large energy jumps of discrete methods by allowing a progressive rearrangement of the local environment. Because this enables both explicit solvation and also better sampling of protonation states, we opted for this variant.

However, without further adjustments, such a continuous titration coordinate leads to a large fraction of simulation time spent in unphysical intermediate states. This is mitigated through λ-coordinate transformation^4,14,37−39^ or a bias potential that favors the physical end states.^40^ Within this family of methods, two distinct but conceptually close approaches have been proposed for the interpolation between protonation states, Hamiltonian interpolation and charge interpolation^41,42^ (more generally, parameter interpolation). Although Hamiltonian interpolation is the physically canonical approach, early implementations faced computational challenges. The need for multiple electrostatic interaction evaluations — one for each protonation state — led to a linear slowdown as the number of protonatable residues increased.^14^ Charge interpolation offers a solution here, achieving better simulation performances that are independent of the number of titratable sites.^18,41,42^ We will discuss the differences between these interpolation schemes in our companion publication,^43^ including their effect on constant pH simulations.

The representation of tautomeric forms of protonatable residues, such as the δ and ϵ forms of singly protonated Histidine, poses an additional challenge in constant pH simulations. This necessitates representing multiple protonated states for a given residue — typically two — rather than a simple binary switch between unique protonated and deprotonated forms, resulting in a three-state model. Continuous titration methods often address this challenge by introducing a dual λ coordinate system,^7^ one λ governs protonation, while the other determines the tautomeric form. Here we adopted a variant of this approach.^44^

Another challenge is that changing protonation states cause a varying total charge of the simulation box. Non-neutral, periodic simulation boxes may cause simulation artifacts,^45−47^ and transitions between differently charged systems under periodic boundary conditions fail to accurately represent real macroscopic systems, which are charge neutral. These effects are particularly pronounced in inhomogeneous systems and when considering free energies,^48^ especially protonation free energies. This issue has been addressed in three main ways: (1) attempting an analytical correction,^18,45,49^ (2) computing an approximate correction specific to the system of interest with a short presimulation,^19^ or (3) using charge buffer particles in bulk solvent to maintain neutrality.^15,40,50^ The latter option, when combined with a sufficiently large simulation box, most closely mimics a real system and is thus our chosen approach.

Finally, the correct treatment of Coulomb forces remains a key challenge in biomolecular simulations due to their long-range nature and computational complexity. Particle Mesh Ewald (PME)^51^ methods are now the *de facto* standard for electrostatics in MD, and are very efficient at low to moderate parallelization, especially when implemented for GPUs.^52−54^ Recently, PME has been successfully used for constant pH simulations.^21,39,41^ However, at high parallelization, global communication becomes a scaling bottleneck,^55^ with the number of messages increasing quadratically with the number of ranks.^56^ This limitation becomes particularly pressing for exascale computing, necessitating the development of Coulomb solvers with improved scaling properties.

We have therefore decided to calculate electrostatic interactions with the Fast Multipole Method (FMM),^57^ which shows improved asymptotic scaling through astute use of hierarchical decomposition, and also avoids unfavorable scaling when combined with Hamiltonian interpolation λ-dynamics. Moreover, FMM surpasses PME in terms of parallelization potential.^58−60^ For instance, in the high parallelization regime, the MODYLAS MD simulation code has successfully deployed FMM electrostatics for a 10 M atoms MD system over 524,288 CPU cores.^61^ Though our FMM implementation as described in more detail in the companion paper^43^ is not currently massively parallel, and thus a typical biomolecule in solution runs about three times slower compared to PME, it has already shown performance advantages for large sparse systems (e.g., droplets or vapor), where it outperforms PME on a single GPU.^62^

To assess the accuracy of our constant-pH implementation, we titrated benchmark proteins, which have NMR-determined residue p*K*_a_ values, namely cardiotoxin V, lysozyme and staphylococcal nuclease. Additionally, we compared the performance of our code with other recent implementations using these benchmark systems.

We further developed novel tools to uncover the underlying drivers of protonation. Using Mutual Information,^63,64^ we uncovered interactions between titratable residues in a model-free manner. Using Functional Mode Analysis,^65,66^ we characterized the couplings between protein conformation and protonation states. This approach yielded both qualitative insights into the coupling mechanisms and quantitative data on conformation-induced p*K_a_* shifts. Our analysis also explains why specific residues pose challenges for accurate titration in constant pH simulations in general.

Recognizing that methodological complexities and setup difficulties have so far impeded the widespread adoption of constant pH simulations, and leveraging the existing expertise of MD users, we integrated our code into the GROMACS^54^ software suite, adhering closely to its established usage and settings conventions. Through carefully selected and assessed default parameters, and extensive setup automation, we demonstrate that an acquainted GROMACS user can simulate a protein using our constant pH code with very little additional effort.

## 2 Methods

We have made the following choices for our GROMACS^54^ constant pH MD implementation: First, we used established techniques, including Hamiltonian interpolation λ-dynamics^35^ with a three-state model for protonatable residue tautomers^7^ and charge buffers for electroneutrality.^40^ Second, we developed Dynamic Barrier Optimization (DBO), a sampling enhancement technique. DBO controls protonation/deprotonation transition rates by dynamically regulating free energy barrier heights. This approach optimizes computational resource utilization. We demonstrate that DBO achieves comparable p*K*_a_ accuracy while accelerating convergence in constant pH simulations.

### 2.1 Constant pH Simulations Based on λ-Dynamics

The λ-dynamics method described here builds upon previous work.^14,35,44,67^ The present section summarizes the main concepts of λ-dynamics-based constant pH simulations, while subsequent sections, starting from 2.3, introduce novel features of our FMM-based implementation.

In λ-dynamics, titratable *sites* — typically protein residues that change their total charge by ±1 upon (de)protonation — are described by a λ-dependent Hamiltonian (36) which is a weighted sum of the Hamiltonians representing the protonated () and the deprotonated forms () of the site. The variable λ linearly interpolates between these Hamiltonians, with each chemically distinct state of a site referred to as a *form*. Sites can encompass not only protonatable residues but also peptide termini, ionizable lipids, and small molecules such as ligands or drug-like compounds.

In contrast to free energy methods like thermodynamic integration^34^ (TI) which use λ as an input parameter to drive the system Hamiltonian from the A to the B state, here, λ is treated as an additional, dynamic degree of freedom — a pseudoparticle with mass, velocity, and consequently, kinetic energy .^35,36^ Forces acting on the λ-pseudoparticle, given by , derive from the following extended Hamiltonian
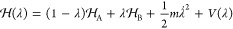
1

To properly control the dynamics of the λ-particle, an additional potential *V*(λ) is introduced, which

(R1) Accounts for the chemical energy difference between protonated and deprotonated states not captured by the molecular mechanics (MM) force field,
(R2) Incorporates the chosen pH by establishing a reference free energy of protonation Δ*G*_0_ for each titratable group,
(R3) Biases the λ-coordinate toward values near 0 or 1, representing fully protonated or deprotonated states, respectively, while disfavoring intermediate, unphysical states,
(R4) Establishes a barrier between protonated and deprotonated state that allows controlling the transition kinetics.

A two-well potential,^14,40,68^ detailed in section 2.2, has been implemented to meet all four requirements (R1–R4). To control protonation and deprotonation rates, achieve high transition rates while avoiding unphysical intermediate states, we dynamically adjust the height of the central barrier as described below (R4).

As in previous work,^44^ a second λ-coordinate is used to alternate between protonation tautomers (e.g., histidine δ and ϵ forms), interpolating between all forms with the following expression (λ̅ ≔1 – λ),
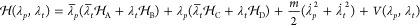
2This scheme can be extended to any number of forms with additional λ-coordinates (which is already supported by our FMM electrostatics solver), although two are sufficient for a constant pH treatment of protein residues and termini. Our current implementation applies the full Hamiltonian (eq 2) to all titratable sites. For sites lacking tautomers, we set  and . Chemically equivalent tautomeric states have the same microscopic p*K*_a_ for the transition toward one or the other tautomer form. This phenomenon is exemplified by the two protonated forms of Glutamate (Glu), which differ only in which oxygen atom of the carboxyl group bears the proton. Conversely, Histidine (His) has one double protonated form, but two chemically distinct single protonated forms (δ and ϵ tautomers) with different microscopic p*K*_a_ values.

In principle, any chemical moiety or residue can be made dynamically protonatable through this scheme. However, because MM force fields are not parametrized to give correct (de)protonation energies, a calibration process, described below, is necessary for each protonatable residue. This computationally intensive procedure is required once for titratable residue types (e.g., Glu, His, ...) in the used force field. This calibration is precalculated here for most relevant residues with a p*K*_a_ close to physiological pH, namely His, Glu, and aspartic acid (Asp) (cf. Supporting Information). Peptide termini are not titratable in the present version of our code.

Depending on the particular force field, the protonated and deprotonated form of a titratable residue ( and ) can differ in ways other than charge, e.g., bond and Lennard-Jones interaction parameters. In our current implementation, we posit that these differences do not affect the dynamics as much as electrostatics. Consequently, we will limit ourselves here to the change of charges of the atoms comprising the sites. All other interaction parameters reflect those of the fully protonated form.

To offer both ease of use and flexibility, most constant pH-related parameters described below are user-controllable via GROMACS input file directives, indicated by the symbol (•). We have defined default settings for protein simulations to mitigate the potential configuration burden arising from this flexibility. These parameters, also employed in our simulations, facilitate reproduction of our settings for other systems, as detailed in section 2.13.

### 2.2 Calibration of Protonation Dynamics

The *V*(λ) term in the Hamiltonian (eq 1) ensures that the protonation behavior produced by the simulation at a given pH satisfies our four requirements R1–R4. This is achieved by calibrating the chemical free energy of protonation against experiments (R1), by coupling the protonation behavior to an external pH bath (R2), concentrating the λ values on physical states (R3), and controlling the (de)protonation rates by a central barrier (R4). Accordingly, we break down *V*(λ) into separate contributions
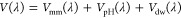
3*V*_mm_ and *V*_pH_ together provide the calibration (R1) and (R2), whereas *V*_dw_ addresses (R3) and (R4). We will now discuss these three individual contributions.

The free energy of protonation derived from an MM simulation, Δ*G*_MM_, does not generally match the actual chemical free energy of protonation, Δ*G*_chem_, as the force field does not take into account the energy of bond cleavage and formation resulting from chemical reactions. To correct this mismatch, Δ*G*_MM_ is calibrated to be equal to Δ*G*_chem_ for reference compounds, using the *V*_MM_(λ) potential, such that constant pH simulation of these reference compounds yields the same p*K*_a_ as experimental titrations.^14^

Therefore, prior to the constant pH simulation, TI simulations of the individual amino acid in solution were performed to determine the free energy landscape along λ over the entire λ interval for the force field used. Defining *V*_MM_(λ) ≔−Δ*G*_MM_(λ) establishes an energy landscape that is perfectly flat for the reference residue at pH = p*K*_a,ref_. Subsequently, when the residue is placed in an environment different from the calibration conditions, e.g., within a protein, the local environment results in a deviation from this flat potential, corresponding to a shift in p*K*_a_, which is the goal of constant pH MD simulations.

Simulations of the reference compound in such a flat potential landscape would yield uniformly distributed λ values, including the unphysical intermediate states. Therefore, to concentrate the distribution on physically meaningful end states around λ = 0 and 1, a double-well potential *V*_dw_ with a central barrier is used. The barrier is chosen high enough to minimize the time spent in unphysical semiprotonated states, but low enough to allow for enough transitions between states to obtain a chosen sampling efficiency.

Finally, to achieve a different population ratio for the protonated and deprotonated forms when pH ≠ p*K*_a,ref_, a pH-dependent potential sets the relative height of the two wells according to the free energy of deprotonation at a given pH
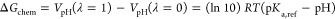
4For details on the TI protocol, see Supporting Information (Section 1).

### 2.3 Partition Function Correction

**Figure 1:**
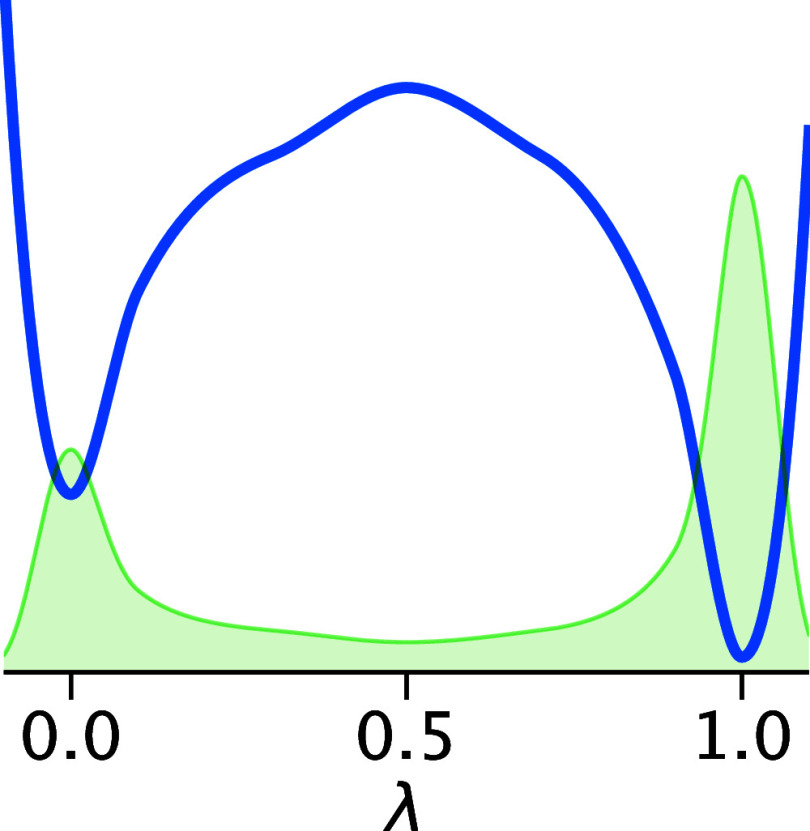
Shape of the double well potential *V*_dw_(λ) (blue), with population histogram in green (at an exaggerated temperature for illustration purpose). The spline corresponding to the shape is defined in the Supporting Information (Section 6).

As a side effect, the typically asymmetrical shape of the two potential wells of *V*_dw_(λ) (Figure 1) exerts an entropic bias on the protonated versus deprotonated population. To compensate for this bias, we calculate the respective partition functions
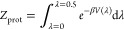
5
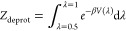
6

Subsequently, the well depths are iteratively refined until the resulting free energy Δ*G* = −1/β ln(*Z*_prot_/*Z*_deprot_) agrees with the target free energy Δ*G* = *k*_B_*T* ·ln 10·(p*K*_a,ref_ – pH).

If enabled (•), the Partition Function Correction is automatically applied when needed, i.e., before the first simulation step, and when adjusting the barrier height or well position (see next section). We emphasize that this correction is not applied *post hoc*; rather, it is computed at runtime, ensuring accurate protonation state ratios during the simulation.

### 2.4 Dynamic Barrier and Well Optimization

The double well potential and Partition Function Correction described above satisfy requirements R1 and R2 (see Section 2.1), while partially addressing R3 and R4. However, two issues remain to be resolved.

First, although the double well potential has wells centered at 0 and 1, the average λ value within each well typically deviates from these ideal values, e.g., when the local environment exerts strong forces on λ through other terms of the Hamiltonian (eq 1), causing the averaged protonated and deprotonated charges to diverge from their physically meaningful integer values.

Second, whereas the central barrier suppresses undesirable intermediate states, it also suppresses transitions between the protonated and deprotonated state, which are necessary for convergence in protonation space. Here, a practical and user-controllable trade-off between sampling efficiency and avoiding nonphysical protonation states is desirable.

To address these concerns, we developed a Dynamic Barrier and Well Optimization (DBO) feature, which dynamically regulate the wells position and barrier height of the double well potential *V*_dw_.

The first algorithm fine-tunes the position of the well to ensure the desired average λ value of 0 and 1, respectively, when in the well. Our implementation automatically collects statistics on the λ values when in the vicinity of each of the wells, as well as the fraction of time spent there, in blocks of 40 ps duration (•). The criteria for proximity to the well located at λ = 0 and 1 is λ < 0.2 and λ > 0.8, respectively. At the end of each of these blocks, if λ has remained more than 70% of the time in the block near one end point, and if the difference between the average λ and the ideal value 0 or 1 (depending on the considered well) is greater than a tolerance of 0.03 (•), the well position is shifted laterally by 0.5 times this difference. A maximum adjustment range (±0.08 (•)) is used to avoid artifacts in highly coupled residues.

The second algorithm adjusts the barrier height for each residue independently (as well as for the protonation and tautomer coordinates within a residue if applicable). It is inspired by the Adaptive Landscape Flattening technique typically used in ligand-binding λ-dynamics.^69,70^ Statistics on the number of frames spent in-transition, defined as satisfying 0.2 < λ < 0.8, are collected in 1 ns blocks (•). The central barrier *V*_dw_ is adjusted up or down in 1.0 kJ/mol increments (•) in a 1–20 kJ/mol interval (•) until the fraction of in-transition frame reaches the given target value, which is 25% (•) for this work, with a tolerance of 5% (•). The starting barrier height is the same as for runs without barrier adjustment, namely 6 kJ/mol (•).

For the tautomer coordinate, two different barriers are used (•), depending on whether the site is protonated or deprotonated, as the force felt in the tautomerically degenerate state is close to zero, and the barrier should therefore be lowered to achieve the desired transition rate in that state. The aim of this algorithm is to make the transition rates of all sites similar, taking into account additional barriers that the local environment of the respective titratable site may or may not induce, and without manual intervention by the user.

Following each adjustment, the λ-trajectory frames are tagged with a censoring flag for a duration of 10 ps (•). These censored frames are not considered during postsimulation analyses to eliminate possible relaxation effects after the small but instantaneous change of *V*(λ). Both the barrier and the well adjustment can be enabled independently if desired (•). In practice, DBO adjustments occur frequently during the first tens of nanoseconds of the simulation, after which the regulated parameters typically stabilize until larger conformational changes occur.

### 2.5 Sites Acting as Charge Buffers

In the context of periodic boundary conditions and free energy calculations, electroneutrality of the simulation box is crucial.^47,71^ To ensure that this requirement is met despite the dynamically changing charges of titratable residues, buffer sites that take up a compensating charge^15,50^ have been used. Water molecules were selected for this role,^50^ with the oxygen atom switching from its charge of −0.834 *e* to +0.166 *e* in the protonated buffer state, as described previously.^44^ An explicit negative buffer state was not necessary, as permanent negative ions (Cl^–^) are automatically added to compensate for a positively charged initial state of the residues during system preparation. In the current implementation, each titratable residue is paired with a corresponding charge buffer site, and the total charge of each pair is kept constant.

To avoid artificial electrostatic interactions between a residue and its buffer site, the distance between the oxygen of the buffer molecule and the C_α_ of the titratable site is restrained to 3 nm (•) (>3 Debye lengths), with a force constant of *k*_restr_ = 50 kJ mol^–1^ nm^–1^ (•). Although a distance of 3 nm was chosen here, longer distances are possible but require *V*_mm_ recalibration.

As the charge buffers should be located in bulk solvent, a minimum distance restraint was also added between each buffer molecule and every C_α_ atom (•), as well as between the buffer themselves, ensuring a distance of at least 2 nm (•). The group of atoms for which restraints are generated can be extended through a user setting to, for example, lipid headgroups, as necessary for the system of interest. The one-sided restraint was achieved with an harmonic potential using the same force constant *k*_restr_ = 50 kJ mol^–1^ nm^–1^ (•), which applies force only when the distance is less than the threshold. This reuses the existing GROMACS distance restraint code and is automatically introduced at the preprocessing stage. The GROMACS genion tool, which places ions during system preparation, has been enhanced to add these buffers automatically, in accordance with the above distance conditions.

We are aware that for larger systems, the number of buffer sites may become so large as to require larger simulation boxes, which decreases simulation speed; a possible future solution is to adopt a strategy that exploits the fact that ’nearly all protonated’ and ’nearly all deprotonated’ states are rare in this case, and can therefore be neglected, thus markedly reducing the required number of buffer sites.^40^

### 2.6 Force Field Modification

The force fields used for this work — and for which this implementation provides full support — are CHARMM36m^72^ and Amber99sb*-ILDN.^73,74^ For the former, the recommended CHARMM-modified TIP3P water model^72^ is used, whereas for the latter, unmodified TIP3P molecules are used.^75^

Glu and Asp residues each have two carboxylate oxygens susceptible to protonation. For technical reasons, in many implementations of constant pH MD, including ours, both of these oxygens carry a hydrogen atom (proton), with the deprotonated states described by assigning zero charge to one or both of the protons. However, charge-neutralized hydrogens tend to adopt an undesirable *anti* conformation that persists after the charge is restored. This *anti* conformation is not an artifact,^76−78^ and may be dominant in certain environments like enzymatic active sites.^79^ However, allowing this conformation renders *V*_mm_ calibration more challenging due to the slow *syn-anti* transition.^76^ As an approximation, we thus chose to enforce the *syn* orientation through a dihedral restraint. This restraint is flat-bottomed and harmonic ( where |ϕ| > ϕ_0_), acting when the angle increases beyond 50° (•) toward the *anti* conformation (ϕ_0_ = 50° = 0.87 rad), with a stiff force constant of *k* = 30 kJ mol^–1^ rad^–2^ (•). The threshold ϕ_0_ was chosen such that |ϕ| is always lower in the protonated state, thus having no influence on the angle distribution in that state while effectively suppressing the *anti* conformation in the deprotonated form.

In Amber99sb*-ILDN, the charges of all atoms, including backbone atoms, differ between protonated and deprotonated forms of each protonatable residue. As the backbone atoms have electrostatic interactions with the side chain atoms of the *N* – 1 and *N* + 1 residues (beyond the 1–3 exclusion), this would require a separate reference compound calibration for each possible pair of neighboring residues, which is impractical. We have therefore chosen to follow previous work on this matter^6,44^ and deviate from the force field-specified backbone charges for ease of implementation. The backbone atomic charges of the deprotonated form were thus reassigned to be the same as in the protonated form. This backbone charge change caused a slight deviation from neutrality over the whole residue, which was compensated by distributing the opposite charge uniformly on the side chain atoms of the deprotonated form. Note that this procedure does not need to be applied to CHARMM36m due to unchanging backbone charges.

### 2.7 Constant pH Simulations with FMM Electrostatics

Among all Coulomb interactions between particle pairs, only a small fraction is affected by changes in λ variables, even for systems with numerous titratable sites. One should therefore expect minimal computational overhead for the constant pH feature. However, a naive use of PME for constant pH necessitates computing electrostatics twice per λ-variable, one for each form of the titratable residue. This duplication arises from the nonlocal nature of the Fourier transform underlying PME, requiring separate charge grids for each protonation state,^14,44^ resulting in prohibitive slowdowns.

We opted for the rigorous Hamiltonian interpolation (eq 1) over charge scaling, despite the good performance of the latter with PME.^41^ This choice required replacing PME with FMM, whose locality maintains low computational overhead. Our FMM implementation, its integration with λ-dynamics, and comprehensive performance analysis are described separately.^43^

### 2.8 Simulations at Constant pH: Parameters and Setup

All simulations used FMM electrostatics and were performed at a temperature of 300 K and a salt concentration of 150 mM NaCl. Simulations were conducted in the NVT ensemble (see also Supporting Information 4.1), as our FMM has not yet been validated with a barostat. We employed the Bussi-Parrinello-Donadio thermostat^80^ with a coupling time τ = 0.1 ps and an integration step size of 2 fs. Specific simulation settings are detailed in the template .mdp files provided in the Supporting Information. We employed standard force field parameters, except for constant pH-specific settings.

#### 2.8.1 Constant pH Parameters

For the λ particles, the Velocity Verlet integrator^81^ with an integration step size of 2 fs was used. The protonation state of the system was saved every 0.5 ps (•). Test simulations suggested a mass of 60 u (•) for the λ particles, as an optimal trade-off between transition rates (affecting titration curve convergence) and integrator stability, which we used for our constant pH simulations. We note that λ-dynamics is rather insensitive to the particular choice of this mass, within an order of magnitude.

The temperature in the λ-subsystem was maintained, as in the MD simulations, by the Bussi-Parrinello-Donadio thermostat^80^ with a coupling constant of τ = 1 ps (•). The λ thermostat is separate from the one acting on the atomic velocities, but here it was set to the same temperature of 300 K (•). Test simulations have shown that this particular thermostat is insensitive to the choice of coupling time for MD simulations in general,^80^ within a 0.01 to 10 ps range, such that τ = 0.1 ps could have been used as well. We additionally verified that the choice of τ in that interval does not affect the p*K*_a_ in constant pH titrations. Our implementation also supports the Andersen thermostat (•) as an alternative.

A 50 ps λ-equilibration run, with position-restrained heavy atoms and a low λ-barrier, preceded all λ-dynamics simulations to ensure initial protonation states compatible with the conformation.

### 2.9 Titration Simulations

Constant pH MD enables computational titration of proteins. This involves simulating the protein at various pH values, using multiple replicas per pH point, to generate an analog of an experimental titration curve for each residue. Such series of simulations are used to determine the p*K*_a_ of these residues as well as to study the pH-dependent behavior of the protein or residue of interest, including conformational change and coupling of protonation. Table 1 summarizes the parameters used for titration simulations for 5 test systems.

**Table 1:** Specifications of Titration Simulations Carried Out With CHARMM36m and Amber99sb*-ILDN

		reference p*K*_a_						
protein or residue	PDB code	Asp	Glu	His	# of titr. residue	# of replicas	time per repl. (ns)	box (nm)
capped Asp, Glu, His		4.0^84^	4.4^84^	6.38^a^^85^	1	20	15	6
pentapeptides GXAXG		N/A	4.08	6.54	2	30	50	6
cardiotoxin V	1CVO^86^	3.65	4.25	6.39^b^	4	40	100	8
hen lysozyme	2LZT^87^	3.65	4.25	6.39^b^	10	40	75	8
SNase mutant ΔPHS	3BDC^88^	3.90	4.36	6.46	19	40	75	8

a Microscopic p*K*_a_ values 6.53 and 6.92,

b Microscopic p*K*_a_ values 6.53 and 6.94.

In the first group of titration simulations, the single residues Glu, Asp and His were titrated, each with both termini capped by the ACE and NME pseudoresidues, which are acetyl and *N*-methyl moieties attached via peptide bonds, at the N-terminus and C-terminus, respectively. Parameters for ACE and NME are available in both the CHARMM36m and Amber99sb*-ILDN force fields. Because these methyl-capped residues are the reference compounds that were used to calibrate the force field compensation potential *V*_MM_(λ), this constituted a self-consistency check, as computational titration should reproduce the reference p*K*_a_ for these residues, including the microscopic p*K*_a_ values for His form δ and ϵ. In recent constant pH works,^18,21^ AAXAA peptides, with X the residue of interest (Glu, His, Asp, ...), have been used as reference compounds instead.^82^ As calibration only corrects for changes in potential energy upon protonation which are not described by the MD force field, there should not be much difference between blocked residues and pentapeptides, so long as the reference p*K*_a_ values used have been measured on these particular compounds. Indeed, as detailed in Supporting Information section 5, these alternative reference compounds are equivalent in terms of calibration.

In the second set of titrations, the pentapeptides GEAEG, GEAHG, GHAEG and GHAHG were titrated, starting from an initial conformation of a straight chain (backbone in the same plane) with extended side chains, generated using the Avogadro software (version 1.2).^83^

The third group was the titration of snake cardiotoxin V from the Chinese cobra *Naja naja atra*.^42,86,89^ The fourth and fifth titration sets used the hen egg lysozyme and *Staphylococcus* nuclease mutant ΔPHS, respectively. Both of these proteins are typical benchmark systems for constant pH methods, thus enabling comparison with previous constant pH implementations.^18,21,41,42,90^

To facilitate comparison with the recent PME-based constant pH implementation in GROMACS,^41,42^ we chose simulation conditions for cardiotoxin V and hen egg lysozyme that matched closely with that work, including the pH range and the reference p*K*_a_ values, allowing for direct comparison of residue p*K*_a_.

Titrations were performed over a pH range encompassing all experimentally reported p*K*_a_ values, extending 1 pH unit beyond extremes in 0.5 unit steps. Protein termini were not titratable, and were set to their respective charged forms. Replica sets began with identical protein structures but different ion positions and initial velocities. To further decorrelate the individual trajectories, each replica was equilibrated for 1 ns prior to the actual sampling run. All titrations were performed both in CHARMM36m and Amber99sb*-ILDN to evaluate force field dependence, as well as with and without Dynamic Barrier Optimization to assess its impact on convergence and accuracy. *Staphylococcus* nuclease was an exception, simulated only with Dynamic Barrier Optimization enabled.

Previous studies have described several finite size effects arising from periodic boundary condition electrostatics that could perturb the p*K*_a_ values of titrated residues.^18,21,45,49,91^ In our constant pH MD protocol, the discrete solvent effect emerges as the most relevant of these effects,^49^ whose influence varies with the solvent number density (number of water molecules per unit volume of the simulation box). Given both our single residue calibration and protein simulations took place at a solvent number density of ≈31 nm^–3^, no correction was necessary in this work. However, systems at significantly different densities require finite size effect corrections to their p*K*_a_ values, as detailed elsewhere.^18,21^ Alternatively, performing residue calibrations at the target density inherently accounts for this effect.

Comparing computational titration results to experimental Nuclear Magnetic Resonance (NMR) data presents challenges. Because directly measuring the resonance of the titrating proton is usually impossible due to the fast exchange kinetics, a nearby nontitrating atom (^1^H, ^13^C, or ^15^N) is used as a reporter instead. The choice of this reporter affects the measured p*K*_a_ values in the reference compound and in the protein. For instance, discrepancies of up to 1 pH unit have been observed in different titration studies of lysozyme, depending on the selected reporting atom.^92^ Direct comparison of p*K*_a_ values with experiment can therefore be problematic, as p*K*_a_ errors can arise from both the limitations of the constant pH method and this systematic measurement bias, making evaluation and improvement difficult. Noticing that λ-dynamics constant pH MD formally does not produce absolute p*K*_a_ values, but Δp*K_a_* to the reference compound, we mitigated this issue by setting our reference p*K*_a_ values to the p*K*_a_ for single residues in solution reported in the NMR titration work cited for pentapeptides and *Staphylococcus* nuclease. Such a reference partially compensates for these measurement effects, and ensures a more direct p*K*_a_ comparability with experimental results. We emphasize that we only used p*K*_a_ values from the single residue titrations and not from the protein, since the latter p*K_a_* values are the reference against which we compare our results. This approach could also have been used for cardiotoxin V and lysozyme, but we chose to use the same reference p*K*_a_ as Aho et al.^41^ for comparison purposes. Reference p*K*_a_ values are configurable in the input files (•).

### 2.10 p*K*_a_ Calculation

For each recorded frame of the constant pH trajectories, every titratable residue was classified as protonated (λ_p_ ≥ 0.5) or deprotonated (λ_p_ < 0.5) according to the respective value of the λ coordinate. We note that whereas frames in intermediate protonation states (0.2 < λ < 0.8) are often discarded by other λ-dynamics constant pH methods, our Partition Function Correction enables inclusion of these frames within the analysis, thereby slightly improving the sampling (See also section 2.3).

For each replica and for each pH value, the deprotonation fraction, which is the ratio *x* = *N*_*p*=0_/*N* between the number *N*_*p*=0_ of deprotonated and the total number *N* of frames, was computed for each pH point and each replica. The resulting titration curve (pH) was fitted to a Henderson–Hasselbalch (H–H) equation,  to determine the p*K*_a_, using SciPy’s nonlinear least-squares routine.^93^ For pentapeptide titrations, we fitted the fractions *x* and pH values to a Hill equation model, , yielding both a p*K*_a_ and a cooperativity parameter *n*. The Hill equation is here an approximation of the general two-protons titration model^15,94^ (eq 7), used to match the NMR titration study of the pentapeptides.^67^

The two microscopic p*K*_a_ values of His (p*K*_a,δ_ and p*K*_a,ϵ_) were determined similarly with a H–H curve, with ratios *x*_micro,δ_ = *N*_p=0,*t*=0_/(*N*_*p*=1_ + *N*_*p*=0, *t*=0_) and *x*_micro,ϵ_ = *N*_p=0,*t*=1_/(*N*_*p*=1_ + *N*_*p*=0, *t*=1_), respectively, with *N*_*p*=0,*t*=0_ the number of deprotonated frames for the δ tautomer, and *N*_*p*=0,*t*=1_ the number of deprotonated frames for the ϵ tautomer.

Error bars were estimated using bootstrapping. Specifically, for each pH point, we resampled with replacement *R* data points from the set of deprotonation fraction *x*, where *R* is the number of replicas for that particular run (see Table 1). The H–H curve fitting was done as described above, to obtain a p*K*_a_ and, where appropriate, a Hill coefficient *n*. This procedure was repeated 5000 times, with reported confidence intervals corresponding to a 5% significance level for both p*K*_a_ and *n*.

For protein residues whose protonation behavior is discussed in the Results section, plots of their respective deprotonated fractions as a function of time are shown in Supporting Information section 2.

### 2.11 Analysis of Direct Residue–Residue Coupling

The titration curves of monoprotic molecules follow the well-known H–H sigmoid curve, but macromolecular polyprotic acids and bases, including proteins, exhibit more complex titration behavior.^94,95^ This behavior arises from protonation coupling, which falls into several qualitative categories, like residue–residue coupling^96−98^ or protonation-conformation coupling^99−102^ (Section 2.12). In this section, we address this first category, coupling between several protonatable residues, typically mediated by electrostatic interaction between them, which we refer to as *direct residue–residue coupling*.

We first screen for interactions between titratable sites using Normalized Mutual Information (NMI), a metric based on the information theoretic quantity Mutual Information (MI),^63,64^ normalized to conveniently adopt values between 0 and 1. Here, NMI quantifies how much the protonation state of one site informs about the protonation state of the other, detecting correlations between the two in a symmetric, quantitative and model-free manner. Once clusters of interacting residues are identified, an effective model of this coupling can be fitted.

To calculate the NMI, we discretized the λ_*p*_ trajectories into binary trajectories. Each frame was classified as protonated or deprotonated based on whether its λ_p_ value exceeded 0.5. In our work, trajectory frames were saved every 0.5 ps, implicitly low-pass filtering the data to mitigate repeat boundary crossing effects. For the trajectories of each replica, pH point and protonatable residue, separately, NMI = 2·*I*(*X*;*Y*)/(*H*(*X*) + *H*(*Y*)) was computed, where *I* is the mutual information and *H* is the information theoretic entropy of the trajectory. This latter term is defined as *H* = −∑_*i*=1_^*N*^*p*_*i*_ ln *p*_*i*_, with *p*_*i*_ the probability of state *i* (among *N*) in each frame, in our case the two protonated and deprotonated states. Both *I* and *H* were computed with the C++ library InfoTheory.^103^ NMI values close to 1 correspond to a high correlation between protonation states, denoting coupling between residues, as opposed to values close to 0, which indicate statistical independence.

We found that the NMI metric can take on spuriously high values when both of the trajectories have very low, but not zero, entropy. This can occur when residues are almost always protonated except for a few frames, sometimes leading to false positives for coupling. To eliminate these, we considered the mean NMI and entropy *H* values over all replicas at a given pH point.

Specifically, we considered the conjunction of mean NMI and mean *H* values larger than an empirically determined threshold of 0.1, across replicas and at any pH point, to be indicative of coupling.

Apart from NMI, other methods to detect coupled pairs exist, typically based on covariance or Pearson correlation coefficient,^104−106^ or on the residue interaction energy in Poisson–Boltzmann-based methods. As NMI is model-free and detects both linear and nonlinear correlation, we expect it to be more sensitive that those methods, though this benefit has yet to be tested in practice. Compared to those methods, NMI also does not distinguish between cooperativity and anticooperativity. It should therefore be followed by the fitting of a titration model on the pairs detected as coupled.

Here, we will fit macroscopic titration curves^15,94^ for such NMI-detected interacting clusters. First, the macroscopic titration curve is extracted from the simulation by averaging the total number of protons bound to this cluster, *X*, with this average, ⟨*X*⟩, being computed for each pH value and each replica. Subsequently, a suitable macroscopic titration equation, e.g., for two interacting residues
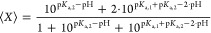
7is fitted. From this fit, the macroscopic p*K*_a_ values p*K*_a,1_ and p*K*_a,2_ are obtained, which describe the collective titration behavior. No clusters involving more than two residues were identified in this work, though the described approach generalizes to any number of protonatable sites.

### 2.12 Analysis of Protonation-Conformation Coupling

The second coupling, mentioned above, is *protonation-conformation coupling*(99−102) and can also be studied using constant pH simulations as follows. This coupling arises from changing protein conformations, which lead to varying local environments for titratable residues and ultimately to shifts in their p*K*_a_. Conversely, changes in protonation state can trigger conformational rearrangements.

This type of coupling can be detected and quantified via Partial Least Squares-based (PLS) Functional Mode Analysis^65,66^ (FMA). While conceptually similar to Principal Component Analysis (PCA) of protein trajectories, FMA identifies collective motions that correlate most with protonation state rather than structural variance.

For each titratable residue, we performed FMA using the protonation state (λ_p_) as the target observable and the heavy atom positions in the protein as the structural ensemble. To detect protonation-conformation coupling, representative structures of the protonated, deprotonated, and intermediate states should be observed in the input trajectory. We achieved this by selecting trajectories from replicas whose pH was closest to the p*K*_a_ of the residue of interest. We kept 20% of the replicas as a validation set to compute the explained variance in protonation. We combined the remaining 80% of trajectories into a structural ensemble, fitting them to the average protein structure’s backbone. The FMA was performed on this ensemble using the first 20 PLS components. The number of PLS components should be large enough to capture most of the variation in λ_p_, and is optimally determined by cross-validation for each residue individually. Across all analyzed residues, we found that the explained variance in protonation plateaued past 10 to 15 PLS components. For consistency and simplicity, we therefore used 20 PLS components for all residues.

**Figure 2:**
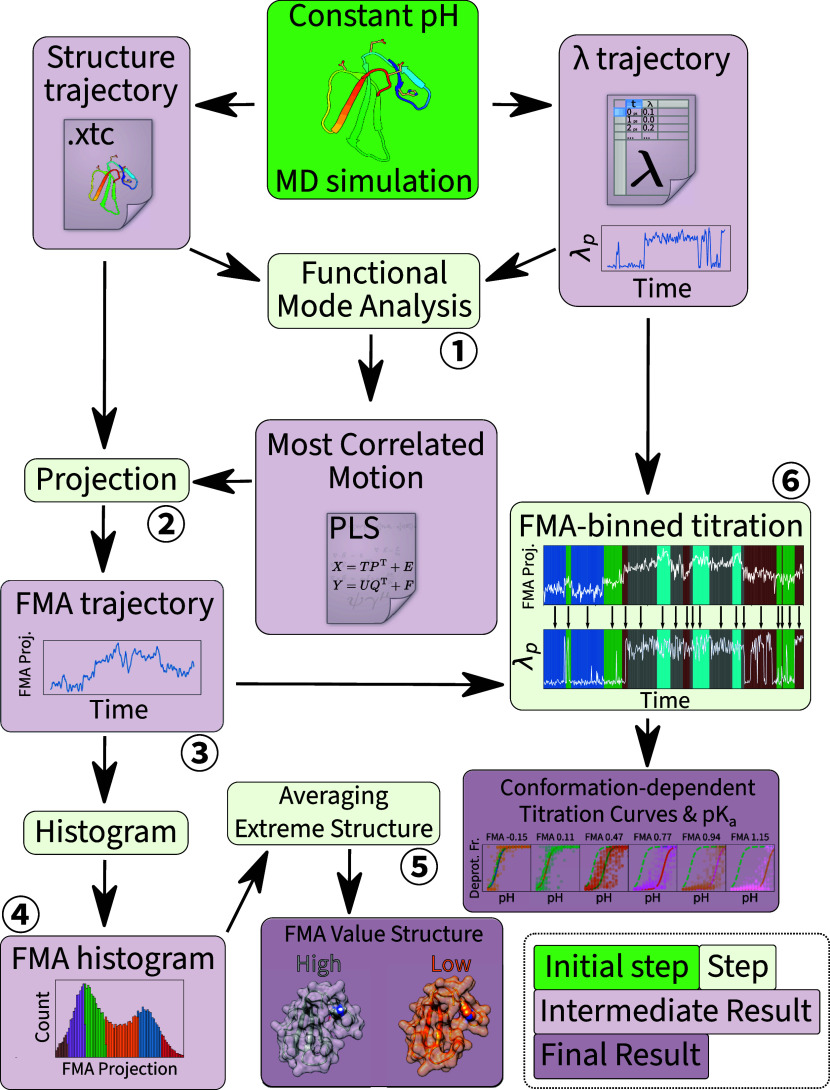
Flowchart of FMA-related analysis, starting with constant pH MD (green), followed by intermediate processing (light green), yielding intermediate (light purple) and final results (dark purple). Circled numbers are referenced in the main text. Icons, plots and data in this figures are for illustration purposes.

Figure 2 illustrates several interdependent analyses we carried out based on the FMA ①. First, we projected ② the structure trajectories of each replica at each pH value onto the most correlated motion. This yielded a one-dimensional projection of the high-dimensional conformational space onto the collective motion best predicting protonation/deprotonation, called an FMA trajectory ③. We then histogrammed these FMA trajectories ④ to create discrete bins based on percentiles (≤fifth percentile, fifth to 25th, 25th to median, median to 75th, 75th to 95th, and finally >95th).

Selecting and averaging ⑤ structures from the highest and lowest bins yielded representative protonated and deprotonated state structures, called *high* and *low FMA value structures*, respectively. These were used in subsequent analyses of the coupling mechanism, to explain the coupling in terms of physics rather than simple motion correlation. Although this is highly system-dependent, productive avenues of analysis include examination of solvent exposure, changes in residue orientation, or movement of charged residues interacting with the protonatable residue of interest.

In parallel to structure averaging, we quantified the p*K*_a_ shift associated with the conformation change. To this end, we grouped all λ trajectory frames according to their respective bins in the FMA histogram, whose boundaries were defined above. For each group, we fitted a H–H titration curve to obtain p*K*_a_ values, as described in Section 2.10. This process, called *FMA-binned titration* ⑥, yields a series of titration curves and p*K*_a_ values as a function of FMA values.

### 2.13 Practical Aspects

**Figure 3:**
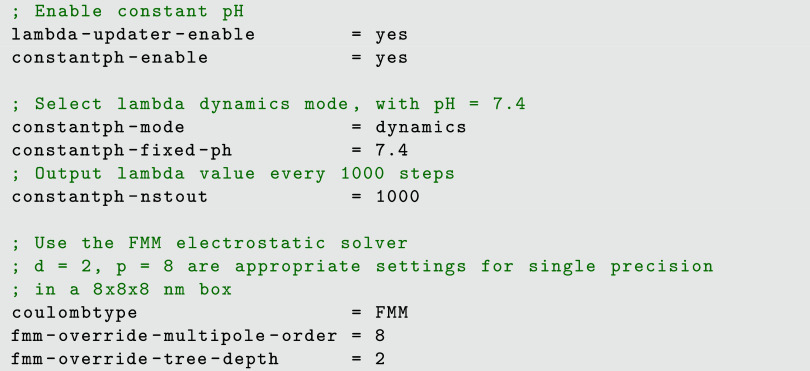
Minimal but fully functional .mdp parameters for constant pH simulations.

The constant pH functionality described above has been integrated into the GROMACS MD simulation software suite,^56,107^ so that users can perform constant pH MD simulations with minimal changes to their existing workflow. Specifically, routine constant pH MD simulations require only a few additions to the MD parameter file (.mdp extension), as exemplified in Figure 3.

The parameters denoted by (•) in the previous sections correspond either to additional directives that may be added to this .mdp file, to modify and adapt the default setup; or to modifications in the topology and force field files.

Starting from any PDB file suitable for non-CPH MD, the pdb2gmx tool now offers new command line switches (-ldglu, -ldhis, and -ldasp) to enable protonation for Glu, His, and Asp, respectively. This tool generates a topology file with the necessary CPH MD information, provided a suitably modified force field is used (see Supporting Information for the CHARMM36m and Amber99sb*-ILDN force field files used in this work)

After protein solvation, the GROMACS tool genion adds both the standard ions and the charge buffer sites for constant pH, automatically placing the latter at appropriate distances.

Following an unmodified energy minimization step, equilibration proceeds with new parameters to control the constant pH code. These additional parameters, parsed as usual by the GROMACS grompp command, are listed in Figure 3, for a constant pH simulation at pH 7.4. While numerous other constant pH parameters are available, those shown in Figure 3 will likely suffice for most scenarios, as we have carefully set appropriate general-purpose defaults for the others.

Simulations are run using the GROMACS command mdrun, with the additional argument -lambdaout specifying the filename for storing λ values for each residue. As in non-CPH MD simulations, the protein trajectory is stored in .xtc or .trr files.

For detailed information on all constant pH parameters, .mdp templates, analysis protocols, and Fast Multipole Method (FMM) settings, we direct readers to the tutorials and documentation available at https://www.mpinat.mpg.de/grubmueller/gromacs-fmm-constantph. The source code can be accessed at https://gitlab.mpcdf.mpg.de/grubmueller/fmm.

## 3 Results and Discussion

Our results are analyzed according to three goals: (1) assessing the accuracy of our CPH method; (2) providing sample applications for our analysis tools for studying protonation coupling; and (3) investigating and addressing sampling issues.

To these ends, we examine test systems of increasing complexity, from single residues for consistency checks, via pentapeptides, cardiotoxin V, lysozyme, and ending with staphylococcal nuclease, a challenging CPH benchmark system. For each system, we compare computed p*K*_a_ values with NMR titration results, then present the results of our analysis methods, focusing on certain protonation coupling relations with interesting properties. Where major differences occur between force fields, we comment on results for both CHARMM36m and Amber99sb*-ILDN; otherwise only for CHARMM36m for brevity. Thus, unless explicitly specified, figures illustrate the results of CHARMM36m simulations.

We conclude by addressing three general topics: (1) sampling improvement via Dynamic Barrier Optimization; (2) the impact of protonation coupling on convergence, and proposed solutions; and (3) overall accuracy of our CPH code, considering force field influence and residue-specific performance (His, Asp, Glu).

Computational performance and implementation details of the Fast Multipole Method underlying our CPH implementation will be presented in our companion publication.^43^

### 3.1 Single Residue Titration

We first performed computational titration on methyl-capped residues: His, Glu, and Asp. As these molecules serve as reference compounds for which calibration data were collected, our titration calculations should recover their reference p*K*_a_ values, providing a self-consistency check of our implementation.

**Figure 4:**
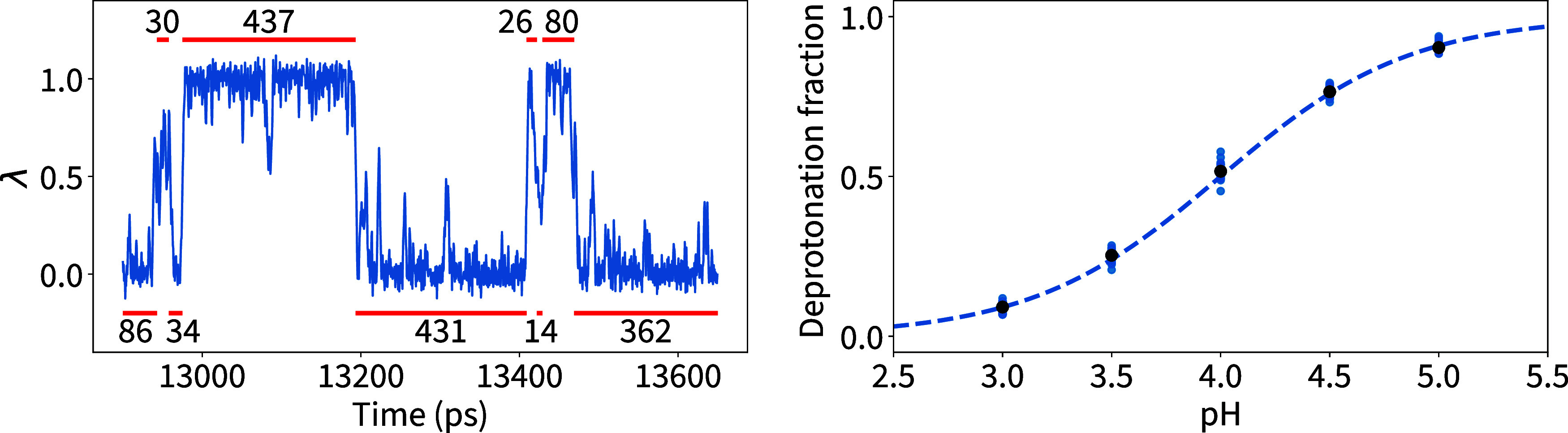
Titration of an Asp residue. Left: Excerpt of a trajectory of the λ_p_ value at pH = 4. Red intervals indicate the number of frames assigned to the protonated and deprotonated states. Right: Resulting titration curve, with blue dots showing the fraction of deprotonated frames for each replica at each pH point. Black dots show the average of all replicas. The dashed blue line is a Henderson–Hasselbalch (H–H) fit.

The computational titration process, described in Methods sections 2.9 and 2.10, is illustrated here in detail for the Asp residue in Figure 4. The left panel shows an excerpt of a λ_p_ trajectory, representing the time-dependent fractional protonation state. Frames are assigned to protonated or deprotonated states (red bars) based on their λ_p_ values, with the number of frames in each state recorded for each replica and pH value. The right panel shows the ratio of deprotonated frames to total frames (’deprotonation fraction’) as a function of the pH at which each simulation was run. Fitting a titration curve, such as the H–H sigmoidal curve (dashed line in the right panel), yields the p*K*_a_ value of the residue. The scatter of the deprotonation fraction for the individual trajectories allows assessment of convergence and p*K*_a_ uncertainty estimation. For these single residues, the small scatter indicates the absence of sampling issues.

Table 2 presents the p*K*_a_ values from our simulated titrations, which exhibit very small uncertainties and align closely with their reference values, constituting a successful self-consistency test for both force fields used. In particular, these results demonstrate that the calibration potential V_MM_ for each residue was correctly implemented, using a polynomial of sufficient degree to accurately fit the free energy surface and with enough sampling for convergence of the calibration simulations, as well as for the computational titrations.

**Table 2:** Summary of p*K*_a_ Values Obtained from Single Residue Titration for For His, Glu and Asp, Using Two Different Force Fields, with Respective Reference p*K*_a_ Values

residue	macro/micro p*K*_a_	CHARMM36m	Amber99sb*-ILDN	reference
His	macro	6.39 ± 0.01	6.36 ± 0.02	6.38
	micro δ	6.53 ± 0.01	6.51 ± 0.02	6.53
	micro ϵ	6.93 ± 0.02	6.88 ± 0.03	6.92
Glu	macro	4.38 ± 0.01	4.36 ± 0.02	4.40
Asp	macro	3.98 ± 0.01	3.98 ± 0.02	4.00

### 3.2 Pentapeptide Titration

Unlike protonatable residues in proteins, the above single residues lack both the influence of neighboring protonatable groups and conformation-linked effects — two phenomena of great interest to CPH MD practitioners. To assess these effects while avoiding sampling issues, we selected a test system of appropriate, small size.

We therefore revisited the pentapeptides from our previous work.^67^ These peptides, with sequence GEAEG, GEAHG, GHAEG and GHAHG, were designed with two protonatable residues (His or Glu) on the same side of the peptide, opening the possibility of electrostatic interactions and (anti)cooperativity behavior, a form of residue–residue coupling.

We first examined whether the simulation duration used, 50 ns per replica, was sufficient to achieve protonation convergence. As can be seen from Section 2.1 of Supporting Information, all replicas yields similar protonation fractions with small scatter, and all titration curves are fitted well by the sigmoidal Hill equation curves. We therefore concluded that the simulations were sufficiently converged.

**Figure 5:**
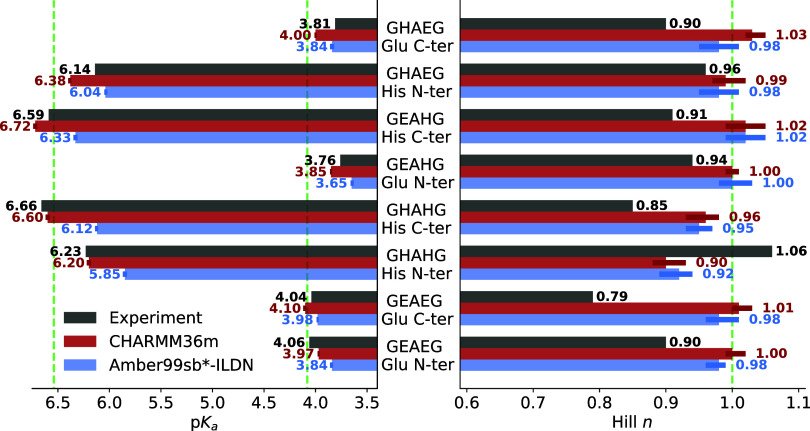
Computational vs experimental titration of short peptides. Shown are p*K*_a_ values (left) and Hill coefficients (right), calculated using CHARMM36m (red) and Amber99sb*-ILDN (blue), and measured by NMR (black). Error bars indicate bootstrapped 95% confidence intervals. The reference p*K*_a_ values for single residues (Glu 4.08, His 6.54) as well as the unity Hill coefficient *n* are shown as dashed green lines, representing the behavior of isolated solvated amino acids for comparison.

Figure 5 compares computational and NMR-measured p*K*_a_ values (left) and Hill coefficients *n* (right). Computational titration accurately reproduces all experimental p*K*_a_ values, capturing both the shift direction relative to isolated amino acids and the absolute values. The overall p*K*_a_ Root Mean Squared Error (RMSE) is 0.13 for CHARMM36m and 0.26 for Amber99sb*-ILDN, with the latter moderately underestimating His p*K*_a_.

In the simulations, all residue exhibited Hill coefficient close to *n* = 1.0 (Figure 5, right panel), meaning that the protonation state of each protonatable residue was largely independent of the protonation state of the second protonatable residue. We also tested this finding using mutual information analysis. Every protonatable residue pair has NMI values below 0.1, often even below 0.01. This confirms that the protonation states of the two titratable residues are uncorrelated.

The simulations thus rule out residue–residue coupling by two separate methods. However, experimental measurements (Figure 5, right panel) contrast with these results, showing Hill coefficients as low as 0.79 for GEAEG. We discussed this discrepancy in our previous work,^67^ which highlights that a straightforward interpretation of the measured Hill coefficient is challenging, complicating direct comparison to constant pH MD results. Notably, this issue does not affect p*K*_a_ value comparisons. Furthermore, including this study, a total of three simulation studies using two different constant pH MD implementations and three different force fields have now obtained similar unity Hill coefficients, in contradiction to experimental results. This apparent discrepancy could be resolved by another experimental titration, ideally including data on the conformational ensemble of the peptides.

### 3.3 Cardiotoxin Titration

Next, we studied the small, 62-residue protein cardiotoxin V, which contains four titratable residues. This protein is widely used as a benchmark system for CPH MD methods, as its small size allows for long simulations, keeping sampling issues under control.

#### 3.3.1 Aggregate accuracy and inter-replica spread

**Figure 6:**
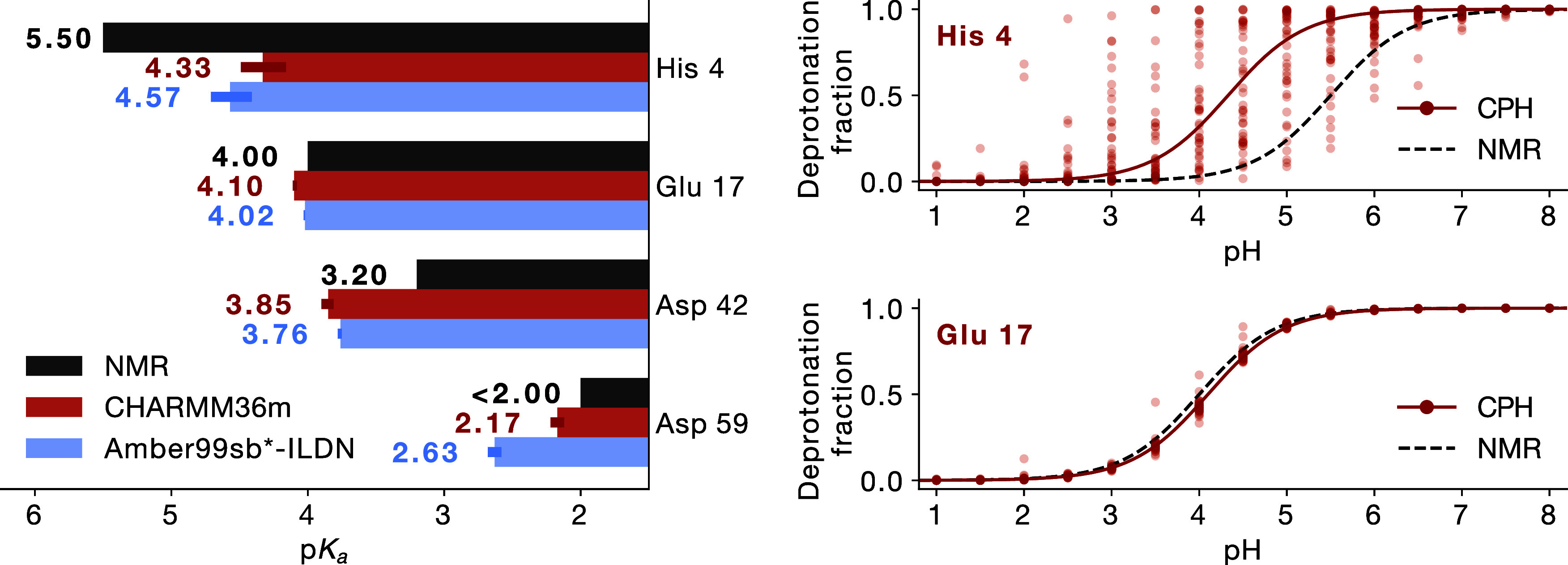
Computational vs experimental titration of Cardiotoxin V. Left: p*K*_a_ values calculated using CHARMM36m (red) and Amber99sb*-ILDN (blue), and measured by NMR (black).^89^ Error bars indicate bootstrapped 95% confidence intervals. Right: Titration curve for His 4 (top) and Glu 17 (bottom), with fraction of deprotonated frames for each replica (point) and the H–H fit to simulation data as solid line. The dashed line is a H–H curve based on the NMR p*K*_a_ values.

We first evaluated the overall accuracy of our constant pH simulations against p*K*_a_ values measured by NMR. To this end, we conducted constant pH MD simulations using 40 replicas of 100 ns duration per pH value, spanning a pH range of 1–8. The overall p*K*_a_ error in these simulations is small (RMSE < 0.7 for both force fields). At the residue level, quite different degrees of convergence and, hence, accuracies are achieved. The right panel of Figure 6 illustrates two contrasting examples. For Glu 17 (bottom), we observed a very small inter-replica spread. Every replica (transparent circle) at each pH point (*x*-axis) shows a similar deprotonation fraction (*y*-axis), indicating good overall convergence of this titration. Its p*K*_a_ is also close to the measured value. In contrast, His 4 (top) displays a wide spread in deprotonation fraction, indicating that each replica experienced a very different free energy of protonation Δ*G*_prot_, causing incomplete convergence of the overall titration. This lack of convergence is associated here with a marked p*K*_a_ error. We observed similar ”spread-replica titrations” for certain residues in all other test systems described below. Elucidating the underlying causes of this Δ*G*_prot_ heterogeneity would help enhance convergence, or reduce the required simulation time to reach a desired accuracy.

To this end, we speculated that this behavior stems from protonation coupling–either between protonation sites or between a titratable group and a slow process like conformational dynamics. To test this idea, we used our analysis tools to detect these couplings, and asked whether or not they correlate with observed spread-replica titration, as well as investigated the mechanism of these couplings.

#### 3.3.2 Analysis of Protonation Coupling

We first searched for direct residue–residue coupling, where the protonation of one titratable residue influences that of another residue, mainly through electrostatic interactions. To this end, we analyzed correlated protonation/deprotonation transitions for all pairs of residues in cardiotoxin V using NMI (Methods section 2.11). This analysis yielded very small NMI values, below 0.1 at all pH, indicating no significant direct residue–residue coupling, an expected outcome given the large separation (>8 Å) between all four titratable residues.

We therefore investigated a possible second coupling type, conformation–protonation coupling, where structural changes shift the protonation of a particular residue, or, vice versa, protonation changes induce structural changes. To examine this coupling, we applied FMA to each titratable residue (Methods section 2.12).

Indeed, for **His 4**, FMA reports that 53% of the protonation variance is explained by conformation coupling. Such strong coupling may thus explain the spread-replica titration seen for this residue.

**Figure 7:**
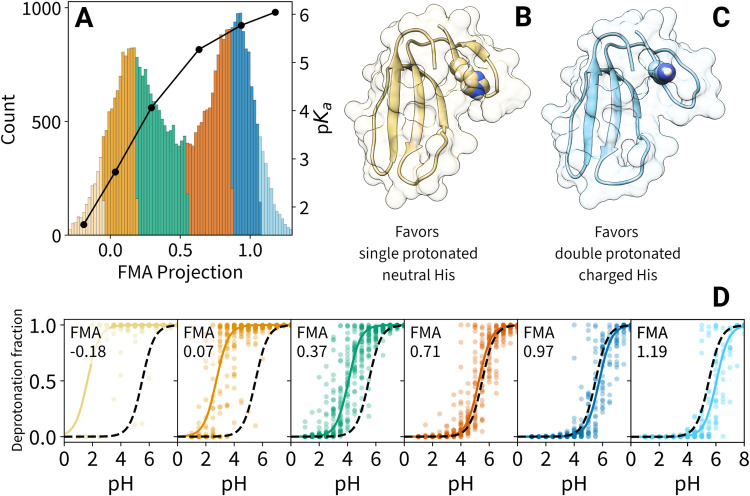
FMA analysis for the His 4 residue of cardiotoxin V. (A): Histogram of FMA projection values for all replicas at pH = 4.5 (left axis) and p*K*_a_ as a function of the projection value (black curve, right axis). (B), (C): Low and high FMA value structure, respectively. (D): Separate titration curves for the six colored bins shown in A (colors correspond), with fraction of deprotonated frames for each replica (points), H–H-fits to these fractions (solid lines), and H–H-curves (black dashed lines) based on the measured p*K*_a_ value.

To characterize this coupling, we examined the histogram of FMA projection values (Figure 7A), which exhibited two broad peaks around 0.2 and 0.8. These peaks represent two main conformations associated with predominantly single protonated and double protonated states of the residue, respectively (Figure 7B,C). To study the motion associated with the coupling, we contrasted protein structures representative of both ends of the FMA value range, obtained by averaging protein conformations within the two outermost FMA bins (Figure 7A, blue and beige sections), yielding what we call the low and high FMA value structures (Figure 7B,C). Key structural differences are evident in the Cys 3 to Tyr 12 loop and the His 4 side chain orientation. At low FMA values (Figure 7B), His 4 is partially buried within the more hydrophobic loop backbone environment, while at high FMA values (Figure 7C), it becomes solvent-exposed. In both conformations, side chains of the loop adapt to accommodate His 4, resulting in additional local structural changes.

To elucidate the relationship between conformational changes and observed protonation states, we computed the p*K*_a_ of His 4 separately for several conformations along the FMA coordinate. These conformations are indicated by different colors in Figure 7A. The resulting titration curves, with corresponding colors, are shown in Figure 7D. We call this process FMA-binned titration (Methods section 2.12). The resulting p*K*_a_ values as a function of the FMA coordinate are shown as a black line in Figure 7A. As can be seen, the solvent-exposed conformation (Figure 7C) shows a p*K*_a_ of 5.7, closer to the solution p*K*_a_ of histidine (6.4), whereas the burying of the histidine within the more hydrophobic environment (Figure 7B) drastically reduces the p*K*_a_ by more than 4 units.

**Figure 8:**
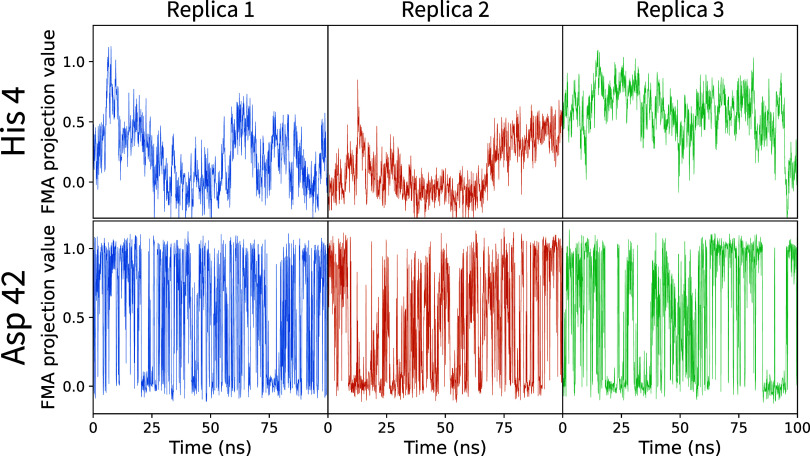
Sample FMA trajectories for the His 4 and Asp 42 residues of cardiotoxin V, for the three first replicas, at pH = 4.0.

These results establish that the conformational state is a major determinant of the protonation of His 4. Examination of the time evolution of the FMA coordinate (Figure 8, top row) shows that the transition between these conformational states occurs very slowly, observed only once or twice within each 100 ns replica simulation. Although we have not established strict causality, it is likely that the protonation fraction follows the conformational dynamics, explaining the slow convergence. Each replica samples a partially distinct subset of conformations associated with this coupling (Figure 8, top row), resulting in varying fractions of deprotonated states, thus explaining the observed large inter-replica spread.

It is instructive to also examine the other three protonatable residues of cardiotoxin V along similar lines. As these residues do not exhibit spread-replica titration to the extent of His 4, they nicely illustrate other possible coupling behaviors.

**Figure 9:**
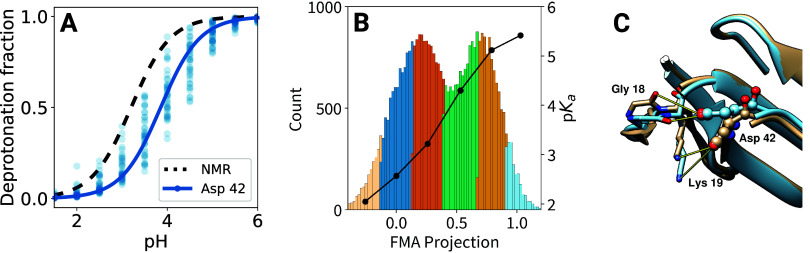
FMA analysis for the Asp 42 residue of cardiotoxin V. (A) Computational titration of Asp 42, with fraction of deprotonated frames for each replica (point) and the H–H fit to simulation data as solid line. The dashed line is a H–H curve based on the NMR p*K_a_* values. (B) Histogram of FMA projection values for all replicas at pH = 4.0 (left axis) and p*K*_*a*_ as a function of the projection value (black curve, right axis). (C) Low and high FMA value structure in beige and cyan, respectively, with pseudobond for relevant stabilizing interactions with Asp 42.

For **Asp 42**, FMA explains 58% of protonation variance, indicating substantial conformation–protonation coupling despite small inter-replica spread (Figure 9A), which warrants closer inspection. Similar to His 4, the FMA analysis (Figure 9B) reveals two main conformations, differing primarily in the Pro 16 – Asn 20 loop facing Asp 42 (Figure 9C). In the high FMA value conformation, Asp 42 forms a hydrogen bond with the Gly 18 backbone carbonyl (distance 2.7 Å), thereby favoring protonation and shifting its p*K*_a_ to 5.8. In contrast, in the low FMA value conformation, Asp 42 forms a salt-bridge to Lys 19 (distance 3.3 Å), thus stabilizing the deprotonated form and lowering its p*K*_a_ to 3.0.

Compared to the rather large conformational change seen for His 4, the side chain reorientation correlating with Asp 42 protonation (Figure 9C) is small and local, suggesting faster kinetics for the Asp 42 coupling. Indeed, more than 35 transitions are seen in the respective FMA trajectories (Figure 8, bottom row), which in turn explains the faster convergence of individual replicas, leading to a smaller spread visible in Figure 9A. These findings demonstrate that protonation-conformation coupling does not necessarily impede convergence by slowing protonation/deprotonation rates.

We note that otherwise identical constant pH simulations with Amber99sb*-ILDN show much smaller coupling for Asp 42 (17% variance explained), and more distant interactions with Gly 18 and Lys 19, underscoring considerable force field dependence.

In contrast to His 4 and Asp 42, FMA analysis of **Asp 59**, located near the C-terminus, explains 27% of its protonation variance, indicating only weak coupling. Correspondingly, the histogram of FMA values shows only one conformation, corresponding to a broad peak near 0.5. The structural dynamics most correlated with the protonation state of Asp 59 are motions involving the C-terminal residues spanning Thr 58 to Asn 62, exhibiting an amplitude under 2.5 Å. There are also no marked changes of interactions to adjacent residues. Despite these rather small changes in the Asp 59 environment, we observed an unexpectedly large p*K*_a_ change with increasing FMA projection value, between 1 and 3 p*K*_a_ points (data not shown). This exemplifies that conformation–protonation coupling does not generally require two distinct conformations. In this case, we similarly observed fast convergence and only very small inter-replica spread.

For the fourth titratable residue, **Glu 17**, the FMA projection explains less than 1% of the protonation variance, thus ruling out any conformation–protonation coupling. Similarly, very fast convergence results (Figure 6).

We emphasize that, although suggestive, we have not yet established here causal connections between conformational changes and protonation states, only correlations. Within the present constant pH framework, this could be achieved by enforcing changes of the respective FMA coordinate, e.g., via Essential Dynamics^108^ using FMA projection as a restrained variable, to see if the expected protonation changes follow. Conversely, enforced protonation changes resulting in corresponding structural changes would establish the reverse causality. Due to microscopic reversibility, both causality directions would be observed at equilibrium. While this makes the concept of causality less meaningful in such conditions, it could still be particularly relevant when studying nonequilibrium processes, such as conformational responses to pH changes, though such investigations lie beyond the scope of this paper.

### 3.4 Lysozyme Titration

Next, we studied Hen Egg-White Lysozyme (HEWL), which is a widely used prototypical benchmark system for constant pH simulations, with several independent NMR titration measurements of its protonatable residues.^92,109^ With ten such residues, HEWL enables us to explore more complex protonation couplings than the four-residue cardiotoxin V system.

#### 3.4.1 Aggregate Accuracy and Outliers

**Figure 10:**
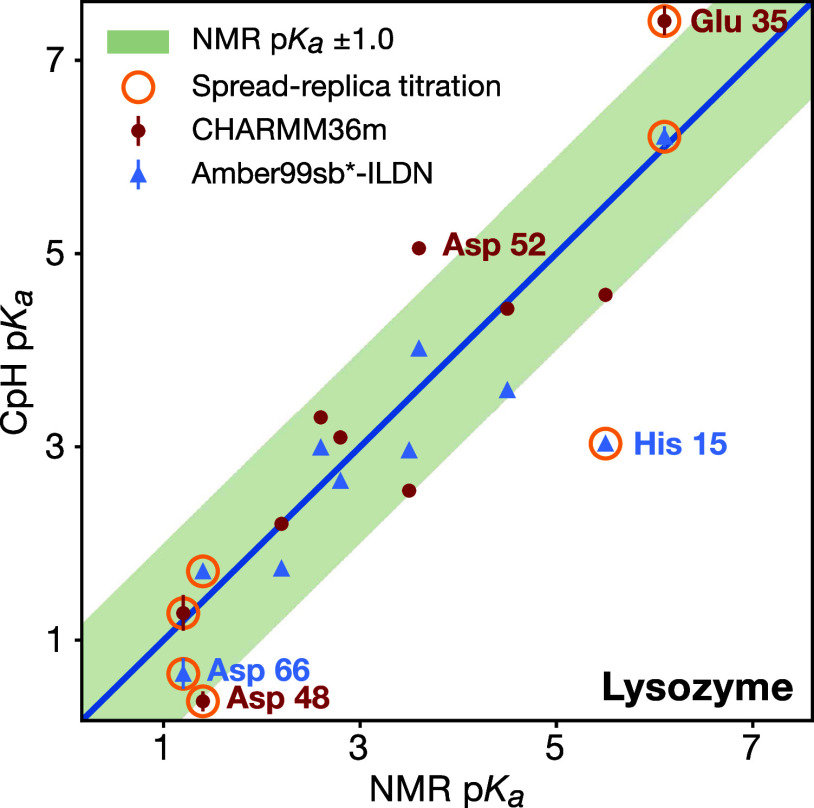
Comparison of residue p*K*_a_ values from NMR titration (*x* axis)^92^ and CPH-MD simulation (*y* axis) in HEWL for CHARMM36m (red circles) and Amber99sb*-ILDN (blue triangles), with bootstrapped 95% confidence intervals as error bars. The green region indicates ≤1 pH point of difference between NMR and simulation. Residues with spread-replica titration are highlighted by orange circles. (p*K*_a_ table available in Supporting Information section 2.3).

We first evaluated the overall accuracy of our implementation for this test system. To this end, we carried out constant pH MD simulations of HEWL, with 40 replicas of 75 ns for each pH point over a range from −1 to 9. Figure 10 compares our constant pH simulation results to measured p*K*_a_ values for all titratable groups, resulting in a p*K*_a_ RMSE of 0.85 and 0.90 for CHARMM36m and Amber99sb*-ILDN, respectively. Most p*K*_a_ values fall within 1 unit of their NMR reference (green region). Our results align with other state-of-the-art CPH implementations, including GROMACS,^41^ Amber,^21^ and CHARMM^18^ simulation codes (RMSE: 0.98, 0.83, and 0.92, respectively).

**Figure 11:**
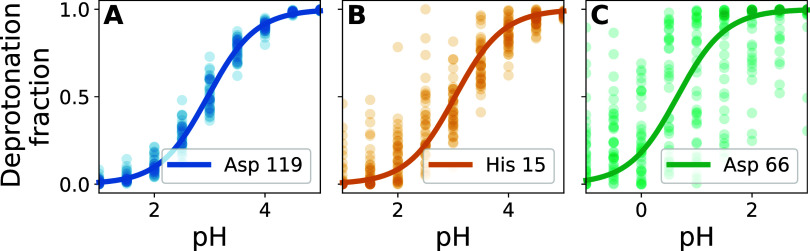
**Computational titration curves of Asp 119, His 15 and Asp 66** for HEWL, using Amber99sb*-ILDN. Transparent points show the deprotonation fraction computed for each replica. The solid line corresponds to the H–H sigmoid fitted to all replicas.

Residues for which less accurate p*K*_a_ values are predicted by our simulations fall into two main categories. The first group comprises those with large shifts from solution p*K*_a_, such as Glu 35 and Asp 48. These shifts are known to be generally challenging for CPH MD codes, because the strong electric fields involved are not accurately described by fixed point charge, nonpolarizable force fields.^110,111^ The second category consists of residues with spread-replica titrations. This effect, already detected for His 4 of cardiotoxin, is also seen to varying degrees in HEWL. In contrast to relatively well-converged residues such as Asp 119 (Figure 11A), spread-replica titrations show mild (Figure 11B), to rather pronounced (Figure 11C) spread in deprotonation fraction across replicas (transparent points), leading to less accurate H–H fits (solid line) and, hence, generally larger p*K*_a_ deviations. In total, seven out of 20 titrations show substantial spread-replica effect (Figure 10, orange circles), including His 15 and Asp 48.

#### 3.4.2 Analysis of Protonation Coupling

We examined residue–residue coupling using NMI analysis (Methods section 2.11) to further study the link between spread-replica titrations and protonation coupling. Only the Asp 48–Asp 66 residue pair showed positive NMI results, with values up to 0.25 at pH = 0, in line with the above-mentioned link.

Given these results, the protonation fractions of this pair are best described by a macroscopic titration curve, which accounts for the coupling (Methods section 2.11). Unfortunately, in the respective titration NMR study, the protonation fraction of the pair is only described by two independent H–H curves, such that the resulting p*K*_a_ values cannot be directly compared. For a fair comparison, we therefore also used two independent H–H curves. We refer the reader to the next system, staphylococcal nuclease, for two examples of macroscopic titration curve modeling.

To test whether the second type of coupling, conformation–protonation coupling, also occurs, we used FMA (Methods section 2.12), focusing on residues which show so far unseen effects. The FMA analysis explains 77 and 51% of protonation variance for Asp 48 and Asp 66, respectively, demonstrating strong protonation coupling for both residues. Together with their relatively large distance of more than 10 Å, this result suggests that conformation–protonation coupling — rather than direct electrostatic interactions — mediate the previously detected residue–residue coupling.

**Figure 12:**
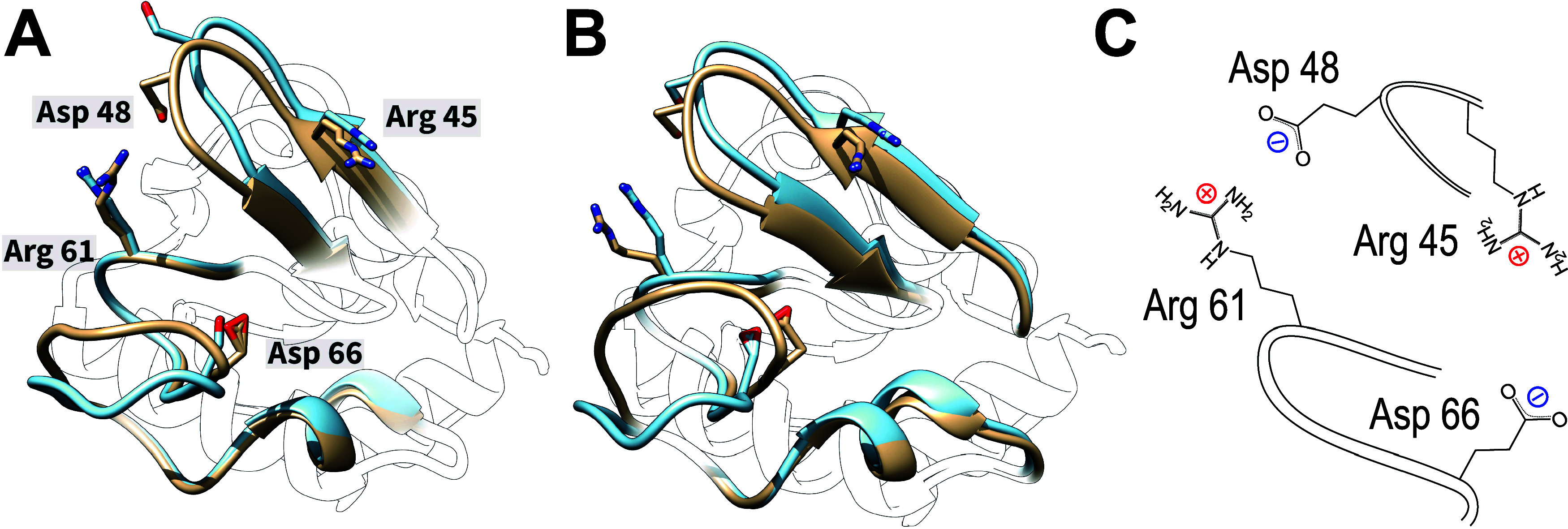
Conformation–protonation coupling in HEWL. Superimposed are the structures corresponding to the FMA projection low (beige) and high (cyan) values for Asp 48 (A) and Asp 66 (B), respectively. Highlighted by color are only those regions which show marked structural differences. (C) Sketch of the proposed coupling mechanism discussed in the text..

Closer inspection of the involved structural changes (Figure 12A,B, beige and blue) revealed by FMA provides further evidence for this scenario. Indeed, the protonation of both residues Asp 48 (Panel A) and Asp 66 (Panel B) correlates with conformational changes of the same regions, involving a loop between Arg 45 and Thr 61, and another loop between Trp 63 and Leu 75. As can be seen, the conformational changes primarily involve a concerted motion of these two loops either toward each other (low FMA values, beige), or apart (high FMA values, blue). This motion suggests a coupling mechanism, sketched in Figure 12C. When Asp 48 and Arg 61 are close, the stronger electrostatic interaction with the positively charged Arg 61 favors the deprotonated form of Asp 48. Similarly, when Asp 66 and Arg 45 are close, Arg 45 may fulfill a comparable role, though weaker due to its greater distance. Because both Asp–Arg pairs are located on the two loops, the concerted motion of the loops changes the distance within each pair cooperatively, such that their respective protonation/deprotonation dynamics occurs jointly. This provides a nice example of protonation coupling mediated via concerted conformational motions. A straightforward generalization of the FMA-binned titration analysis, used previously for single residues, to the binning of protonation states along more than one FMA coordinate would provide a more quantitative picture, but is not implemented so far.

The remaining spread-replica titrations, Glu 35 and His 15, exhibited strong conformation–protonation coupling, akin to His 4 in cardiotoxin V (Section 3.3), further corroborating our hypothesis linking coupling and spread-replica titration. Of note, the His 15 coupling manifests solely in Amber99sb*-ILDN simulations, similarly to Asp 42 in cardiotoxin V, where only CHARMM36m showed a coupling, again underscoring that force field choice and comparisons are critical.

### 3.5 Staphylococcal Nuclease Titration

Next, we studied staphylococcal nuclease ΔPHS (**pH S**tabilized mutant), our final protein system. This enzyme is, like HEWL, a widely used constant pH simulation benchmark.^18,21,112^ It constitutes a challenging test of our implementation, owing to its high density of titratable residues (twice that of lysozyme), many of which are buried and/or adjacent to other protonatable sites.

#### 3.5.1 Aggregate Accuracy

**Figure 13:**
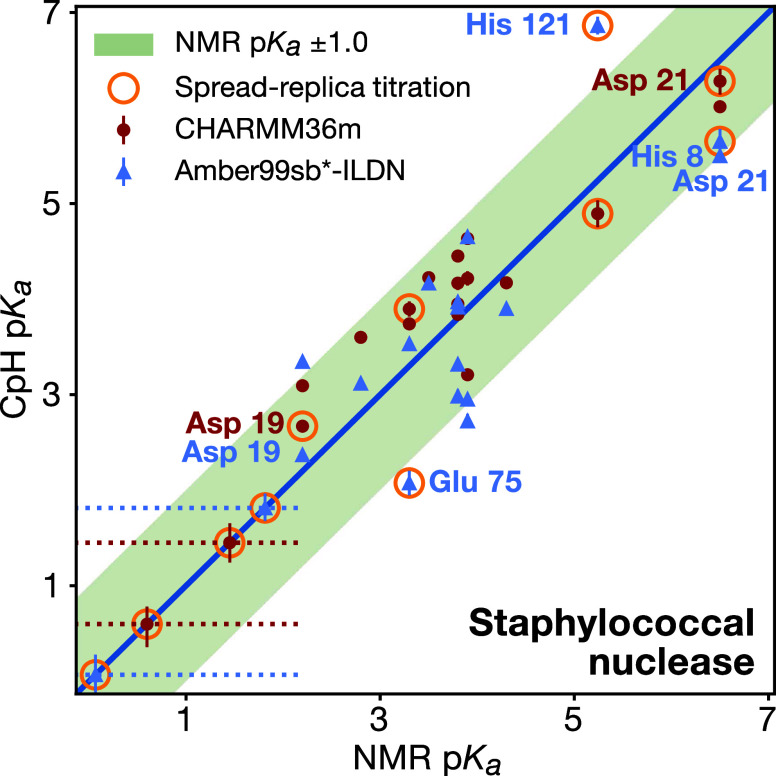
Comparison of residue p*K*_a_ values from NMR titration (*x* axis)^88^ and CPH-MD simulation (*y* axis). Same as Figure 10, but for staphylococcal nuclease. Dashed horizontal lines mark residues for which only a measured upper bound of 2.2 was reported, compatible with the corresponding p*K*_a_ values calculated from CPH simulations. (p*K*_a_ table available in Supporting Information section 2.4.)

We first examine the overall p*K*_a_ accuracy of our constant pH implementation for this test system. To this end, we carried out constant pH MD simulations, split in 40 replicas of 75 ns for each pH value within a range from 1 to 8. Figure 13 compares our simulation results to measured p*K*_a_ values for all titratable groups, demonstrating that virtually all titrations fall within 1 pH unit of the NMR reference values (green region). Our simulations yield p*K*_a_ RMSEs of 0.53 and 0.83 for CHARMM36m and Amber99sb*-ILDN, respectively. Notably, the CHARMM36m simulations demonstrate excellent accuracy for this challenging system, outperforming other state-of-the-art constant pH codes, including the Amber code^21^ (RMSE 0.76) and the CHARMM code^18^ (RMSE 0.80), both of which use the CHARMM22 force field. Our Amber99sb*-ILDN simulations, while less accurate than our CHARMM36m simulations, remain competitive with these other codes.

We attribute the improved accuracy of our CHARMM36m simulations to two factors: (1) the use of the more recent CHARMM36m force field (versus CHARMM22) and, (2) much longer simulation times and, hence, better convergence. We gathered a total of 3 μs of simulation per pH point across all replicas, compared to the 10 to 40 ns of previous studies reported here for comparison,^18,21^ corresponding to a hundredfold increase in sampling. This enhancement was facilitated by FMM-driven performance increases,^43,59,60^ and our use of 40 replicas (versus 3–5 previously). This approach allows for parallel execution of relatively short simulations, markedly enhancing sampling at affordable wallclock time. As discussed in section 3.7, some residues exhibit slow, coupled titration dynamics, a particularly salient issue in the staphylococcal nuclease system, which is ameliorated by increased sampling.

**Figure 14:**
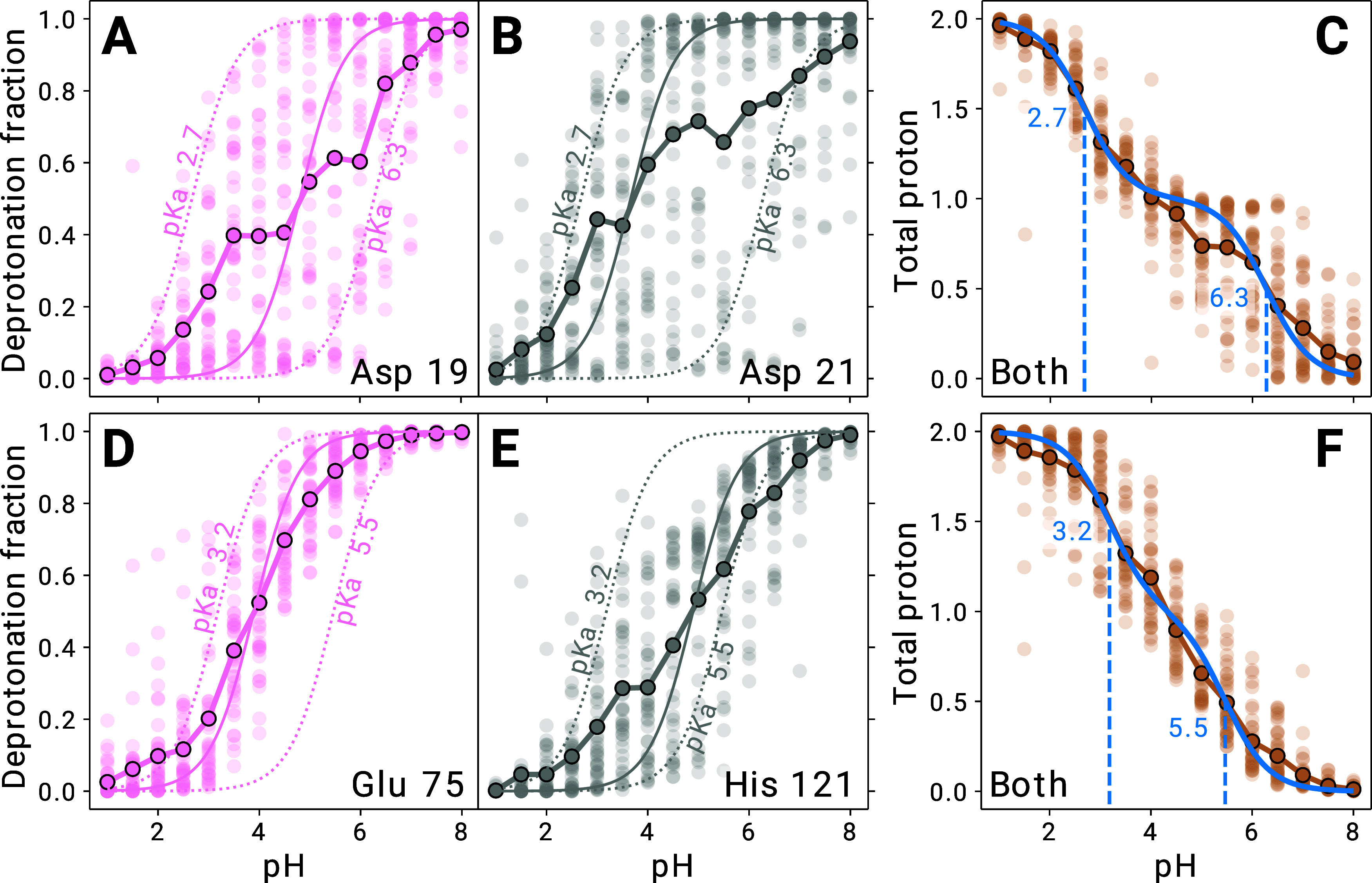
Residue–residue coupling in the Asp 19 (A) – Asp 21 (B) and Glu 75 (D) – His 121 (E) pairs of staphylococcal nuclease. (A,B,D,E): Computational titration curves for individual residues. (C,F): Macroscopic titration curves for the pairs, i.e., number of protons bound to both residues. Deprotonation fraction (A, B, D, E) or number of proton bound (C, F) shown for each replica (transparent circle) and averaged across replicas (black outlined circles). Fit to both per-residue and macroscopic titration curves shown as solid lines. Inflection points in the macroscopic titration curve (dashed vertical lines) mark the macroscopic p*K*_a_ values, with the corresponding H–H curves shown as dashed lines.

As observed in the HEWL system, errors in p*K_a_* values primarily stem from inaccurate electrostatics for residues with large p*K*_a_ shifts, such as Asp 21 and Asp 77, as well as spread-replica titrations (Figure 13 orange circles). Following our earlier approach, we investigate couplings in this system to test the hypothesized correlation between protonation coupling and spread-replica titration. In particular, the Asp 19 – Asp 21 pair exhibits notable inter-replica spread in deprotonation fraction in our simulations (Figure 14A,B). Castaneda et al. identified this pair as highly coupled in their NMR titration study.^88^ We therefore explored this pair in more detail to test whether such strong coupling is also seen in our simulations, and whether quantitative agreement with experiment is achieved for this complex case.

#### 3.5.2 Asp 19 – Asp 21 Pair

To this end, we first examined the titration curves obtained from the simulations (Figure 14, top row). As can be seen, the titration curves for Asp 19 and Asp 21 exhibit substantial inter-replica spread (transparent circles). Interestingly, the average deprotonation fraction across all replicas (black outlined circles) shows marked deviation from a sigmoid titration curve, indicating pronounced residue–residue coupling.

Indeed, an NMI analysis yielded values of 0.20 and 0.17 for CHARMM36m and Amber99sb*-ILDN, respectively, underscoring strong residue–residue coupling. In contrast to the Asp 48 – Asp 66 pair in HEWL, the close proximity of Asp 19 and Asp 21 suggests that their coupling arises from direct electrostatic interactions, i.e., that the negative charge of a deprotonated Asp disfavors the deprotonation of its neighbor, resulting in protonation anticooperativity.

In further contrast to the Asp 48 – Asp 66 pair, an NMR study using the appropriate macroscopic titration curve is available for Asp 19 – Asp 21 (Methods section 2.11). This enabled a more direct comparison between simulation and experiment. Indeed, the macroscopic titration curve provided a better fit to the data, as shown in Figure 14 (A and B versus C), thus demonstrating quantitative agreement. With this model, the two residues Asp 19 and Asp 21 show macroscopic p*K*_a_ values of 2.67 ± 0.05 (±0.05: bootstrapped 95% confidence interval) and 6.28 ± 0.14, respectively. Our secondary force field Amber99sb*-ILDN results in macroscopic p*K*_a_ values of 2.37 ± 0.05 and 5.65 ± 0.13 for these two residues. Both p*K_a_* value sets closely align with the NMR reference values of 2.21 and 6.54. In contrast, the microscopic p*K*_a_ values 4.8 and 3.7 for Asp 19 and Asp 21, corresponding to a simple H–H fit, do not match the NMR reference, which indicates that a coupled modeling is mandatory for the pair. Our constant pH implementation thus accurately reproduces the Asp 19 – Asp 21 coupling in simulations, behaving remarkably close to the experimental data.

#### 3.5.3 Glu 75 – His 121 Pair

Given these encouraging results, we systematically applied NMI analysis to all residue pairs of the test system to identify other coupled titrations. This analysis revealed an additional coupled pair, Glu 75 – His 121, exhibiting maximum NMI values of 0.14 and 0.18 for the CHARMM and Amber simulations, respectively — comparable in strength to the above Asp 19 – Asp 21 pair. Consequently, we employed the same modeling approach, transitioning from individual titration curves (Figure 14D,E) to a two-proton macroscopic titration (Figure 14F).

The macroscopic p*K*_a_ values for Glu 75 and His 121 are 3.18 ± 0.09 and 5.46 ± 0.08 for the CHARMM simulation, and 2.04 ± 0.13 and 6.90 ± 0.09 for the Amber one. Unlike our approach, the NMR titration study^88^ reported individual p*K*_a_ values (3.30 and 5.24) rather than using a macroscopic titration curve. Therefore, we also used the H–H p*K*_a_ (microscopic p*K*_a_) values for comparison with the NMR data, namely 3.90 ± 0.08 and 4.89 ± 0.14 for CHARMM; and 2.08 ± 0.12 and 6.86 ± 0.09 for Amber, respectively.

Interestingly, the titration curve for Glu 75 is nearly sigmoid, despite the coupling (Figure 14D), which likely explains why this coupling was not detected by NMR. In fact, it might also have been overlooked by a qualitative examination of simulation titration curve, underscoring the value of our NMI analysis.

Given that Asp 19, Asp 21, Glu 75 and His 121 were all found to be coupled, as well as being spread-replica titration, our idea of a link between these two characteristics seems to hold for the staphylococcal nuclease system too.

#### 3.5.4 Analysis of Conformation–Protonation Coupling

Two residues with spread-replica titration remain, Asp 77 and Asp 83, which did not exhibit residue–residue coupling. Thus, we investigated all residues for a second possible kind of coupling, conformation–protonation coupling, using FMA.

Indeed, the residue pairs exhibiting direct residue–residue coupling (Asp 19 – Asp 21, Glu 75 – His 121) also display conformation–protonation coupling. These residues show minor conformational changes between low and high FMA value structures, primarily involving side chain motions without significant backbone rearrangements (Supporting Information section 3.2). Though the FMA projections explain a relatively large proportion of their respective protonation variances (56, 69, 47 and 38%, respectively), these variance figures include some contribution from residue–residue coupling, as the coupled residues are involved in the FMA-derived motions. This mixing of influence is precisely what makes our approach unable to quantify the relative strength of both types of coupling if they co-occur. However, the small motion amplitudes suggest residue–residue coupling predominates.

Beyond these pairs, Asp 77 and Asp 83 also demonstrate conformation–protonation coupling in staphylococcal nuclease (Figure 13, indicated by the four points with dashed *x*-axis error bars), with FMA explaining 56 and 72% of their variance in protonation, respectively. The p*K*_a_ values of these residues are exceptionally low in both NMR and our simulations (1.35 and 0.50), falling below the global acid unfolding transition at pH 2 for this protein,^88^ thus precluding exact p*K*_a_ measurement. The structural changes associated with the FMA projections are broad changes in conformation of loops near Asp 77 and 83 (cf. Supporting Information Figure S48 and S49). We therefore speculate that the observed conformational coupling might also involve primary unfolding steps.

The conformational coupling affecting Asp 77 and Asp 83 further corroborates the system-wide relationship between coupling and spread-replica titrations. This phenomenon, now observed across cardiotoxin V, HEWL, and staphylococcal nuclease, is robustly supported by our data. Beyond identifying coupling, this pattern offers a foundation for exploring potential solutions, which we will address in subsequent sections.

### 3.6 Dynamic Barrier Optimization

We have seen above that convergence, e.g., for titration simulations, critically depends on the transition rates between the protonated and deprotonated state. To improve the accuracy of such calculations, it is therefore beneficial to artificially increase these rates. To this end, we have implemented Dynamic Barrier Optimization (DBO), which tunes the double well potential *V*_dw_ to bring these transition rates to a desired value, for each residue during the simulation (Methods section 2.4). DBO has been used in all simulations presented so far. Here we characterize its effectiveness by comparing simulations with and without it.

**Figure 15:**
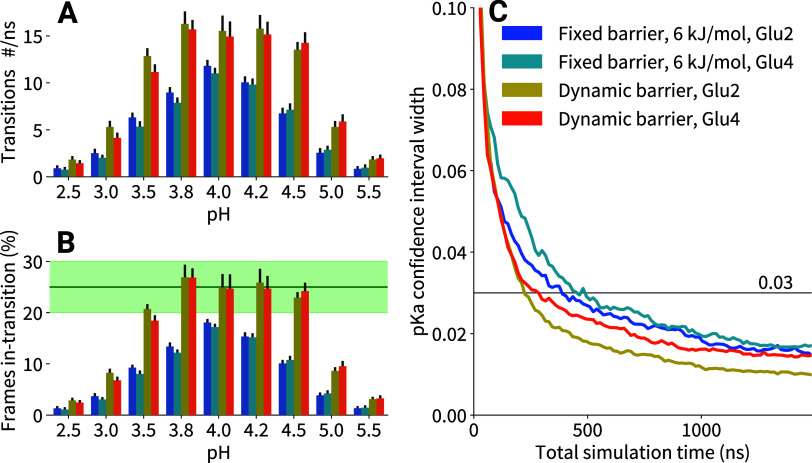
Effect of Dynamic Barrier Optimization (DBO) on protonation transition rates and p*K*_a_ convergence for the GEAEG pentapeptide. Transition rates (A) and fraction of unphysical lambda values (B) observed in titration simulations for selected pH values, (C) precision in terms of p*K*_a_ confidence intervals widths. The colors indicate fixed barrier (blue, green) vs DBO (yellow, red) for residues Glu2 (blue, yellow) and Glu4 (green, red), respectively. The maximum tolerated unphysical fraction of frames (25%) with 5% threshold is indicated as a green bar in panel B; the 0.03 confidence interval (black line) in panel C indicates our convergence criterion. Black bars show standard deviations.

As an illustration, Figure 15 compares simulations of the pentapeptide GEAEG with and without DBO. DBO results in markedly increased transition rates (panel A) as well as percentages of frame in-transition, defined by 0.2 < λ_p_ < 0.8 (panel B). The latter is directly monitored by DBO, which adjusts the height of the inter-well barrier to reach its target value, here 25% with a 5% tolerance. While these results mean DBO is effective at adjusting rates, convergence remains the primary goal. In the absence of a direct observable reporting on convergence, we rely on proxy measurements, here the p*K*_a_ uncertainty and degree of homogeneity between replicas. Panel C thus plots the width of the confidence interval (CI) on p*K*_a_ as a function of total simulation time, with lower CI width indicating less uncertainty, greater homogeneity, and thus more converged simulations. For both Glu in GEAEG, DBO markedly accelerates the decrease in CI width over time, reaching our target width for fully converged simulations (0.03 pH points) 40% faster — a 200 ns reduction in total simulation time. The improvement in transition rates brought about by DBO thus also translates into improved convergence.

Here, we test our DBO implementation to (a) verify that similar p*K*_a_ values are obtained, and (b) if and to what extent convergence is actually enhanced. We conducted titration simulations for both cardiotoxin V and HEWL, with and without DBO, employing 40 replicas each. Each replica ran for 100 ns for cardiotoxin V and 75 ns for HEWL. Consistent with our previous example (Figure 15B), the target value for the percentage of frame in-transition (0.2 < λ_p_ < 0.8) was 25% with a tolerance of 5%.

**Figure 16:**
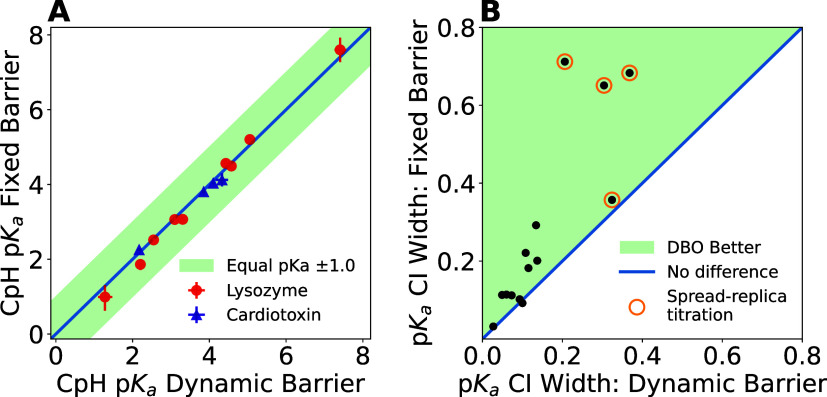
Impact of DBO on the titration of cardiotoxin V (blue) and HEWL (red). (A) Comparison of p*K*_a_ values with and without DBO, with the green region indicating difference ≤1 p*K*_a_ point. Error bars are 95% confidence intervals. (B) Comparison of precision in terms of p*K*_a_ confidence interval width. Residues for which the confidence interval is narrower when DBO is used lie in the green region, and residues with spread-replica titration are highlighted by orange circles.

DBO introduces a time-dependence in the double well potential *V*_dw_ that could produce artifactual changes in residue p*K*_a_, for instance if the protonation state of a residue affected its protonation transition rate. We hypothesized this effect to be negligible, as DBO adjusts barrier height at most every nanosecond, much slower than changes in the λ degree of freedom, and only rarely past the first five to ten nanoseconds of the simulation. We nonetheless investigated this possibility by comparing the p*K*_a_ values of all residues in both titrations (Figure 16A), revealing an average agreement within 0.15 p*K*_a_ units and a Pearson’s correlation coefficient of 0.99, with no outliers. We conclude that DBO does not introduce any such unwanted artifacts.

We next examined the impact of DBO on convergence, using the same proxy as in the illustrative example above (Figure 15C), namely p*K*_a_ uncertainty as measured through CI width. Figure 16B compares the CI width with (*x*-axis) and without (*y*-axis) DBO for each residue, at the end of the simulation. As can be seen, DBO reduces p*K*_a_ uncertainty for essentially all residues, demonstrating that the artificially lowered energy barrier indeed increases the protonation/deprotonation rates and, hence, the statistical precision of the resulting protonation fractions. As should be expected, the most substantial precision increases occur for those residues with the largest inter-replica spread (orange circles), resulting in p*K*_a_ CI width improving from ±0.7 to ±0.3 (95% interval). Residues with already high transition rates showed no further improvement from DBO, but importantly, experienced no adverse effects.

Although DBO substantially improved sampling and reduced p*K*_a_ uncertainty for most residues, some (e.g., His 4 in cardiotoxin V, Glu 35 in HEWL) still exhibit slow convergence. DBO acts on the barrier of the double-well potential *V*_dw_, which is only one of the factors affecting protonation transitions. Other barriers exist, such as (anti)cooperativity from residue–residue coupling and conformational rearrangements in protonation-conformation coupling. These barriers can only be partially compensated through DBO. We thus analyze these additional barriers in detail in the following subsection.

### 3.7 Protonation Coupling and Convergence

We have already seen for our test systems above that the protonation/deprotonation dynamics of a given residue can be coupled to the protonation state of other residues and also, to conformational dynamics. In such cases, the protonation/deprotonation dynamics may be markedly slowed down, e.g., by slow conformational dynamics. Such situation can be detected by examining the lack of convergence of the deprotonated fraction as a function of time (cf. Supporting Information Section 2), or simply through the spread in deprotonated fraction shown in the titration curve (spread replica titration). This section outlines additional techniques to monitor and accelerate convergence in these situations, specific to the underlying coupling.

#### 3.7.1 Conformation–Protonation Coupling

In fact, conformational dynamics is very often far slower than DBO-enhanced protonation dynamics. In such cases, the convergence of protonation fractions and, hence, p*K*_a_ values is limited by conformational sampling. This is a challenge unrelated to constant pH simulations, of course, that has prompted the development of many methods to address it. Among these techniques, some are untargeted, accelerating all protein degrees of freedom, such as parallel tempering,^113,114^ or replica exchange with solute tempering (REST).^115^ Other techniques require the definition of a collective variable, like weighted ensemble path sampling,^116^ essential dynamics,^117,118^ flooding,^119^ or metadynamics.^120^ These latter methods require knowledge of the precise nature of the conformational motion coupled to the protonation dynamics.

As an example, Figure 8 shows the projection of the trajectory of the cardiotoxin V protein (simulated with CPH MD) onto the collective conformational motion that exhibits the largest correlation with the protonation states of His 4 and Asp 42, respectively, as determined from Functional Mode Analysis. Strikingly different behavior is seen in these two cases. His 4 (top row) shows very slow conformational dynamics, with long plateaus and infrequent transitions between them, such that each replica samples a different region of conformational space, resulting in slow convergence. Conversely, Asp 42 (bottom row) displays frequent transitions, indicating well-overlapping conformational ensembles. This results in well-converged protonation fractions and p*K*_a_ values.

#### 3.7.2 Residue–Residue Coupling

We now turn to residue–residue coupling. This coupling manifests either directly through electrostatic interactions between adjacent residues, or indirectly via conformational changes. Both mechanisms can be analyzed in terms of protonation microstates, with the latter also amenable to the conformational dynamics methods described earlier.

For a pair of coupled residues, a microstate describes the joint protonation state of both residues, e.g., residue 1 protonated and residue 2 deprotonated (written A_1_H/A_2_^–^). The fraction of time spent in each possible microstate is determined by individual p*K*_a_ values, pH, as well as by the energetics of the coupling. In the absence of any coupling, *P*(A_1_ ∧ A_2_) = P(A_1_)·P(A_2_) (where A_1_ and A_2_ denote either protonation state), i.e., the protonation of each residue is independent of that of the other. Any deviation points to a coupling free energy of
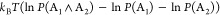


**Figure 17:**
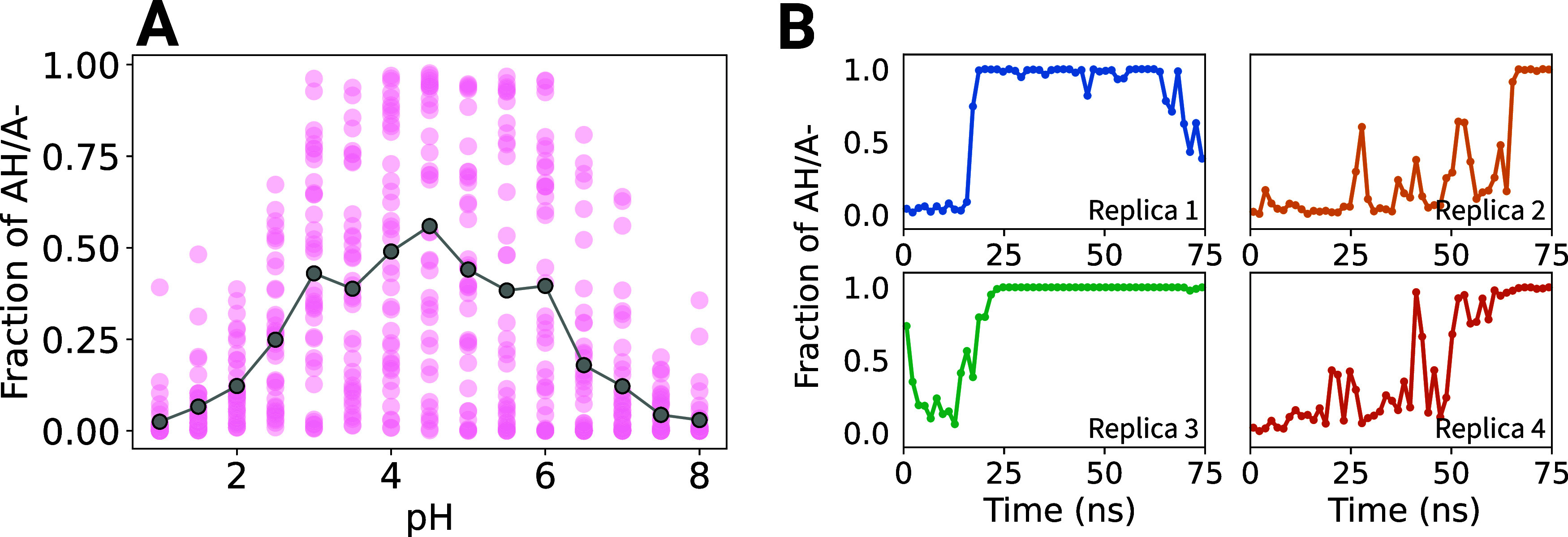
Residue–residue coupling and microstate fraction heterogeneity in staphylococcal nuclease. Shown is the fraction of time during which Asp 19 is protonated and, simultaneously, Asp 21 is deprotonated (A_1_H/A_2_^–^ microstate). (A): as a function of pH, for each replica (transparent circle) and averaged across replicas (black outlined circle). (B): as a function of time for four replicas at pH 4.0, computed in 1.5 ns windows.

Generalizing the single residue treatment above, the scatter of microstate fractions across replicas indicates the level of convergence. To illustrate this convergence evaluation method, we examine the Asp 19 – Asp 21 coupled residue pair. Figure 17A depicts the fraction of the A_1_H/A_2_^–^ microstate (protonated Asp 19, deprotonated Asp 21) as a function of pH, revealing a substantial spread between replicas in the pH range of 2 to 7 and indicating incomplete convergence.

Figure 17B shows examples of the time-dependent behavior of the A_1_H/A_2_^–^ microstate fraction, characterized by long plateaus with infrequent transitions. These rare transitions indicate high barriers between microstates, accounting for the observed spread in microstate fractions across replicas. While our analysis readily detects this incomplete convergence, our current implementation does not provide automated solutions. In these exceptional cases, it may help to fix the protonation state of one of the coupled residues while allowing the other to titrate freely, and *vice versa*. Possible future implementations may extend DBO to multiple residues and/or incorporate biasing potentials akin to conformational flooding^119^ to accelerate microstate transitions.

Similar slow convergence of coupled residues has been observed across constant pH MD formulations, including His 161 in β-Lactoglobulin dimers with the stochastic titration method,^106^ His 142 in BBL 1 protein with all-atom PME λ-dynamics in CHARMM,^18^ and His 4 in cardiotoxin V using another, independently developed, GROMACS λ-dynamics implementation.^41^ These results underline that slow convergence is not specific to a particular technique or implementation.

To further enhance sampling, several other methods, not yet implemented in our code, could be employed: pH replica exchange,^20^ as in the CHARMM^18^ and Amber^21^ constant pH codes; adaptive landscape flattening, developed for the Brooks group’s λ-dynamics code;^70^ or integration of λ degrees of freedom into collective variable suites like Colvars^121^ or PLUMED.^122^

### 3.8 Overall p*K*_a_ Accuracy

**Figure 18:**
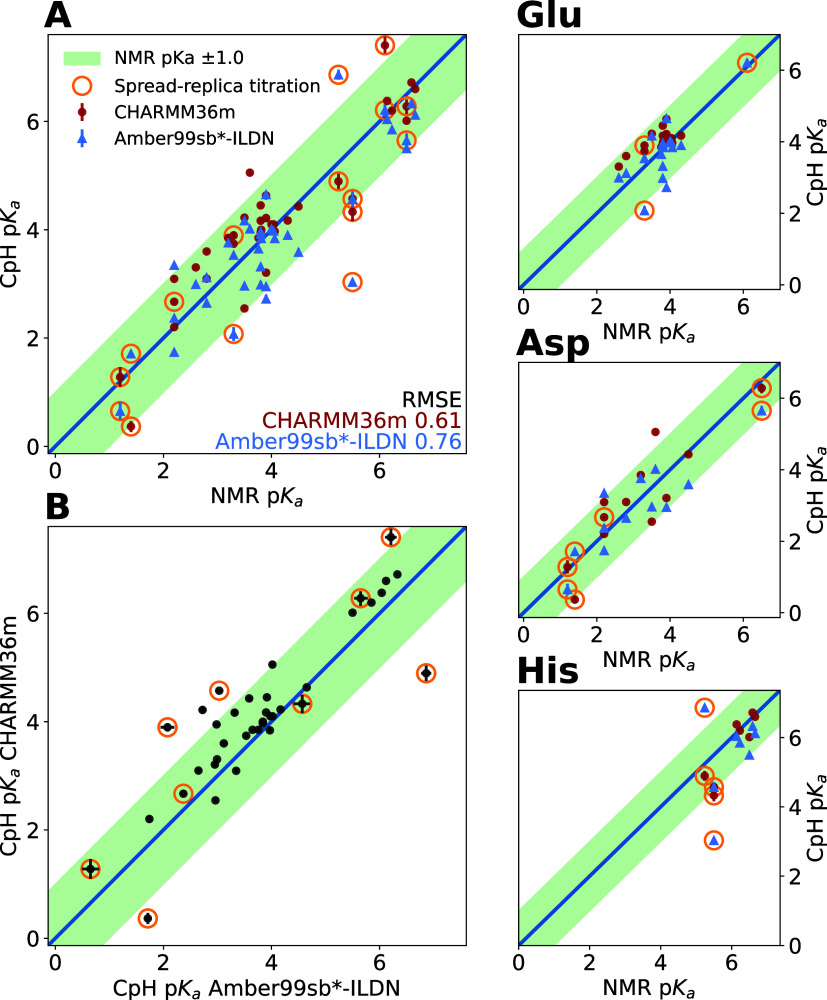
Aggregate comparison between calculated and measured p*K*_a_ values for CHARMM36m (red circles) and Amber99sb*-ILDN (blue triangles), with bootstrapped 95% confidence intervals as error bars. The green region indicates ≤1 p*K_a_* point deviation. Residues with spread-replica titration are highlighted by orange circles. (A): Comparison between NMR and constant pH p*K*_a_ values for all residues. (Glu), (Asp), (His): for specific residue types. (B): between CHARMM36m and Amber99sb*-ILDN.

Figure 18 finally summarizes the overall accuracy of our CPH implementation across all benchmark systems (excluding single residues). For the 38 titrated residues with measured p*K*_a_ values, our implementation achieves p*K*_a_ RMSE values of 0.61 and 0.76 with CHARMM36m and Amber99sb*-ILDN, respectively (Figure 18A). These RMSE values are similar to those from other explicit solvent constant pH methods,^18,21,41,42,90^ indicating that our FMM-based λ-dynamics approach achieves state-of-the-art p*K*_a_ accuracy.

When the p*K*_a_ values and their accuracy are grouped according to amino acid type, we find that the most abundant residue, Glu, with 18 occurrences (Figure 18, Glu panel), exhibits the best accuracy, with RMSE values of 0.54 and 0.55 for CHARMM and Amber, respectively, significantly better than the overall p*K*_a_ RMSE of 0.61 and 0.76. In our test systems, Glu typically exhibits p*K*_a_ shifts below 1.5 units, posing less challenge for constant pH methods. More demanding are the 12 Asp residues (Figure 18, Asp panel), with a markedly broader range of p*K*_a_ shifts up to ±2.5 p*K*_a_ units. Nevertheless, very good agreement with experiments is achieved, with an RMSE of 0.72 and 0.66 for CHARMM and Amber, respectively, and only one p*K*_a_ value deviating by more than one unit. Interestingly, for the eight histidines (Figure 18, His panel), CHARMM36m predicts comparably accurate p*K*_a_ values (RMSE 0.58), while Amber99sb*-ILDN achieves lower accuracy (RMSE 1.18). Our implementation thus demonstrates a robust reproduction of the p*K*_a_ of Asp and His across the p*K*_a_ spectrum (the latter only with CHARMM36m).

Regarding convergence, we noted a clear correlation between spread-replica titrations (Figure 18, orange circles) and large p*K*_a_ shifts with respect to the solution p*K*_a_ of each respective residue type, most evidently for Asp and His. As expected, the correlation between spread-replica titration and less accurate p*K*_a_ reproduction, observed for individual systems, also holds at the aggregate level. Indeed, all but two residues with >1 p*K*_a_ units difference between calculated and experimental p*K*_a_ exhibit this property (Figure 18A). Notably, excluding these unconverged residues yields a p*K*_a_ RMSE of 0.55 for both force fields, highlighting the potential gains from improving the titration of these residues.

To assess the force field dependence of our calculated p*K*_a_ values, Figure 18B compares the p*K*_a_ values obtained with CHARMM36m and Amber99sb*-ILDN. These values are in good agreement, with a Pearson correlation of 0.89. A minority of residues show p*K*_a_ differences exceeding 1 pH point between the two force fields. Across all test systems, the p*K*_a_ RMSE improves to an excellent 0.57 when averaging p*K*_a_ values per residue between both force fields, outperforming both our own individual force field simulations, and other constant pH studies^18,21,41,42,90^ (using a single force field). This improved accuracy could stem from either cancellation of errors between force fields, or increased sampling from having effectively combined both titration series. To differentiate these effects, we recalculated the averaged p*K*_a_ RMSE using half the sampling per titration series, matching the total sampling of single force field simulations. This calculation yielded a p*K*_a_ RMSE of 0.61, identical to the CHARMM36m simulations, suggesting that increased sampling was the primary factor for the improved accuracy. Spread-replica titrations further support this hypothesis, demonstrating that unconverged titrations, which benefit from additional sampling, are present in our results. Although force field biases likely contribute to p*K*_a_ error — for instance His in the Amber ff19sb and ff14sb force field being shown to be systematically worse than CHARMM c22^123^—we identify insufficient sampling as the primary issue to address in future work, given that (1) the root issue–protonation coupling–remains a significant challenge in current CPH MD codes, and (2) well-converged titrations are a prerequisite to force field tuning for p*K*_a_ reproduction.

### 3.9 Conclusions and Outlook

This study presented design decisions and applications of a new implementation of Hamiltonian interpolation λ-dynamics constant pH MD within the GROMACS MD suite,^54,56,107^ supporting both CHARMM36m and Amber99sb*-ILDN force fields, which we tested on a variety of systems from simple pentapeptides to proteins. We demonstrated the feasibility of using Hamiltonian interpolation for constant pH protein simulations, previously deemed impractical due to computational costs stemming from Particle Mesh Ewald (PME)^51^ electrostatics calculations. To circumvent this problem, our implementation therefore incorporated a Fast Multipole Method implementation, whose role is detailed in our companion publication.^43^

We validated our approach through computational titrations of constant pH benchmark systems (cardiotoxin V, lysozyme, and staphylococcal nuclease), yielding p*K*_a_ values comparable to NMR measurements, with overall p*K*_a_ RMSEs of 0.61 and 0.76 for CHARMM36m and Amber99sb*-ILDN force fields, respectively. Notably, averaging p*K*_a_ values on a per-residue basis across both force fields yielded an improved RMSE of 0.57. However, we also observed marked correlations between p*K*_a_ errors, large inter-replica spread in titrations, and residues subject to protonation coupling. Based on these findings, we posit that enhancing sampling for coupled residues offers the most promising avenue for improving CPH MD accuracy.

Across our test systems, our implementation achieved similar or better accuracy compared to recent constant pH studies,^18,21,41,42,90^ with the aforementioned averaged p*K*_a_ values across force fields reaching a remarkable 0.57 p*K*_a_ units RMSE. Notably, for the challenging staphylococcal nuclease system, known for its pronounced inter-residue and protonation-conformation coupling, we attained an RMSE of 0.53 using CHARMM36m, surpassing previous reports.

To accelerate and control protonation/deprotonation transitions during CPH simulations, we implemented Dynamic Barrier Optimization (DBO), which dynamically adjusts the heights of barriers separating protonated from deprotonated states, thereby enhancing sampling and computational titration accuracy. Our results demonstrated that DBO significantly improves convergence without compromising p*K*_a_ accuracy, particularly for notoriously slow-converging coupled residue pairs.

Furthermore, we developed postsimulation tools to analyze protonation couplings in proteins, encompassing both protonation-conformation coupling and inter-residue protonation state coupling, which provided considerable insight when applied to our benchmark systems. Our FMA-based analysis identified substantial p*K*_a_ shifts related to conformational changes, such as one exceeding 2.5 pH units for His 4 in cardiotoxin V. Using mutual information analysis, we detected coupling in both well-known interacting residue pairs (e.g., Asp 19 – Asp 21 in staphylococcal nuclease) and previously unidentified pairs (e.g., Glu 75 – His 121). These tools also characterized the degree of convergence for individual residues, highlighting that coupled residues converge much more slowly than isolated ones. Consequently, our analysis tools not only detected couplings but also revealed causes of slow convergence, supporting the development of future convergence enhancement methods. Future development of our code will thus focus on implementing sampling improvement techniques such as pH replica exchange.^21^

## Appendix

An enhanced version of GROMACS that enables constant pH simulations is available for download; please follow the instructions at

https://www.mpinat.mpg.de/grubmueller/gromacs-fmm-constantph.

Development can be followed at our git repository

https://gitlab.mpcdf.mpg.de/grubmueller/fmm, and documentation can be found at

https://grubmueller.pages.mpcdf.de/docs-gromacs-fmm-constantph

## Supporting Information Available

Compressed archive file containing the force field files for our constant pH enabled version of CHARMM36m (ZIP)
Compressed archive file containing the force field files for our constant pH enabled version of Amber99sb*-ILDN (ZIP)
Compressed archive file containing a GROMACS .mdp input parameter file template to be used to run the titrations as presented here (ZIP)
Residue calibration, titration results, titration coupling analysis results, and the effect of pressure equilibration (PDF)
