# Supplementary material for: Constant pH Simulation with FMM Electrostatics in GROMACS. (A) Design and Applications
Source: J Chem Theory Comput. 2025 Feb 7;21(4):1762–86. doi: 10.1021/acs.jctc.4c01318 (PMC11866755; doi:10.1021/acs.jctc.4c01318)
Supplement: Supplementary file 4 — ct4c01318_si_004.pdf [file ct4c01318_si_004.pdf]

— Supplementary Information —

Constant pH Simulation with FMM Electrostatics in GROMACS. (A) Design and Applications

Eliane Briand, Bartosz Kohnke, Carsten Kutzner, and Helmut Grubmüller*

Theoretical and Computational Biophysics, Max Planck Institute for Multidisciplinary Sciences, Am Fassberg 11, 37077 Göttingen, Germany

E-mail: hgrubmu@mpinat.mpg.de

Contents

1 Details of the TI protocol **2**

2 Titration Details **4**

2.1 Pentapeptide Titration 4

2.2 Cardiotoxin V 14

2.3 Hen Egg White Lysozyme (HEWL) 23

2.4 Staphylococcal Nuclease 34

3 FMA Projections **51**

3.1 Cardiotoxin Asp 59 51

3.2 Staphylococcal Nuclease 52

4 Simulations Setup Details **56**

4.1 NVT Simulations 56

5 Alternative references compounds for calibration **58**

6 Double well potential definition **59**

References **61**

1 Details of the TI protocol

We collected the derivatives $\langle \partial \mathcal{H}_{\text{ref}} / \partial \lambda \rangle_{\lambda}$ at discrete points along the λ coordinate. Tautomerism was included by considering a two-dimensional free energy landscape $V_{\text{MM}}(\lambda) = V_{\text{MM}}(\lambda_p, \lambda_t)$ (see eq 2 of the main text). Accordingly, the derivatives were collected on a 2D grid, with either $\lambda := \lambda_p$ or $\lambda := \lambda_t$, computed using

$$\frac{\partial V_{\text{MM}}}{\partial \lambda} = \left\langle \frac{\partial \mathcal{H}_{\text{ref}}(\lambda)}{\partial \lambda} \right\rangle_{\lambda_p, \lambda_t} = \left\langle \frac{\partial V_{\text{ref}}^{\text{Coul}}}{\partial \lambda} \right\rangle_{\lambda_p, \lambda_t} \tag{1}$$

for combination of values of λ_p and λ_t. The last relation, equating $\mathcal{H}_{\text{ref}}$ (the full Hamiltonian) to $V_{\text{ref}}^{\text{Coul}}$ (the electrostatic potential energy) holds here because the protonated and deprotonated form of a residue differ only by charge.

In our implementation, a polynomial is fitted to $\partial V_{\text{MM}} / \partial \lambda$ as collected on the (λ_p, λ_t) grid, with each λ from the following set of value: -0.1, -0.05, 0.0, 0.05, 0.1, 0.2, 0.4, 0.6, 0.8, 0.9, 0.95, 1.0, 1.05, 1.1 (step of 0.2, and a finer step of 0.05 around 0 and 1), which we empirically found to be sufficient granularity for protein residues. For example, the

point ($\lambda_p = 0.1$, $\lambda_t = 0.6$) was used. At every grid point, $\partial V/\partial\lambda_p$ and $\partial V/\partial\lambda_t$ were collected for 60 ns to thoroughly average over all conformations of the residue.

Calibration is specific to each residue and to each supported force field (here, CHARMM36m and Amber99sb*-ILDN), as well as to the distance between the individual residue and its charge buffer site (though the distance is fixed to 3 nm here, it is possible to calibrate for any distance), and the ionic strength of the solution. The 2D grids are fitted to polynomials of degree 5 in both dimensions (•), including all mixing terms (e.g., $c_{2,3} \cdot \lambda_p^2 \cdot \lambda_t^3$), which we empirically found in test calculations to provide sufficient accuracy for proteins.

The time required for convergence was determined by repeating the TI and the fit procedure for three fully independent replicas and comparing the resulting fit surfaces. For the final calibration data provided as an archive in the Supplementary Information, all three replicas were used to fit the polynomial.

Note that, as a consequence of calibrating against a reference which is a single amino acid in solution (eq 4 of the main text) constant pH simulations produce ΔpK_a to the reference compound, not absolute pK_a. We nonetheless report absolute pK_a for ease of interpretation, in line with practices in the field.

To allow for a flexible control of the height of the central barrier and the position, shape, and depth of the two wells independently, we represent the potential $V_{\mathrm{dw}}(\lambda)$ using cubic Hermite splines (c.f. Supplementary Information Section 6).

2 Titration Details

2.1 Pentapeptide Titration

Total period removed from analysis due to dynamic barrier/well adjustment less than 3 ns per simulation, typically less than 1 ns.

Peptide	pH points
GEAEG	2.5, 3.0, 3.5, 3.75, 4.0, 4.25, 4.5, 5.0, 5.5
GHAHG	5.0, 5.5, 6.0, 6.25, 6.5, 6.75, 7.0, 7.5, 8.0
GEAHG	2.0, 2.5, 3.0, 3.25, 3.5, 3.75, 4.0, 4.5, 5.0, 5.5, 6.0, 6.25, 6.5, 6.75, 7.0, 7.5, 8.0
GHAEG	2.5, 3.0, 3.5, 3.75, 4.0, 4.25, 4.5, 5.0, 5.5, 5.75, 6.0, 6.25, 6.5, 7.0, 7.5

In the following plots, the transparent points correspond to the deprotonated fraction for individual replicas of constant pH MD simulation, the black cross to the mean of all replicas at a given pH point, and the solid line to the Hill equation fit based on these CPH data points. The dashed black line is the Hill equation curve for the pK_a and Hill n measured in NMR.

Table 1: Computational titration of pentapeptides: pK_as and Hill coefficients n for CHARMM36m compared to NMR-measure pK_a values. CI95 = 95% confidence interval obtained by bootstrapping. NMR single residue pK_a: Glu 4.081, His 6.54. mean absolute error = 0.11, RMSE = 0.13, Max abs. deviation = 0.24.

		Constant pH simulation				Experiment[1]	
Peptide	Residue	pK_a	CI95	n	CI95	pK_a	n
GEAEG	Glu N-ter	3.97	3.96 – 3.97	1.00	0.99 – 1.02	4.06	0.90
	Glu C-ter	4.10	4.09 – 4.11	1.01	1.00 – 1.03	4.04	0.79
GHAHG	His N-ter	6.20	6.18 – 6.22	0.90	0.88 – 0.93	6.23	1.06
	His C-ter	6.60	6.58 – 6.62	0.96	0.93 – 0.98	6.66	0.85
GEAHG	Glu N-ter	3.85	3.84 – 3.86	1.00	0.99 – 1.03	3.76	0.94
	His C-ter	6.72	6.70 – 6.74	1.02	0.99 – 1.05	6.59	0.91
GHAEG	His N-ter	6.38	6.37 – 6.40	0.99	0.97 – 1.02	6.14	0.96
	Glu C-ter	4.00	3.99 – 4.01	1.03	1.02 – 1.05	3.81	0.90

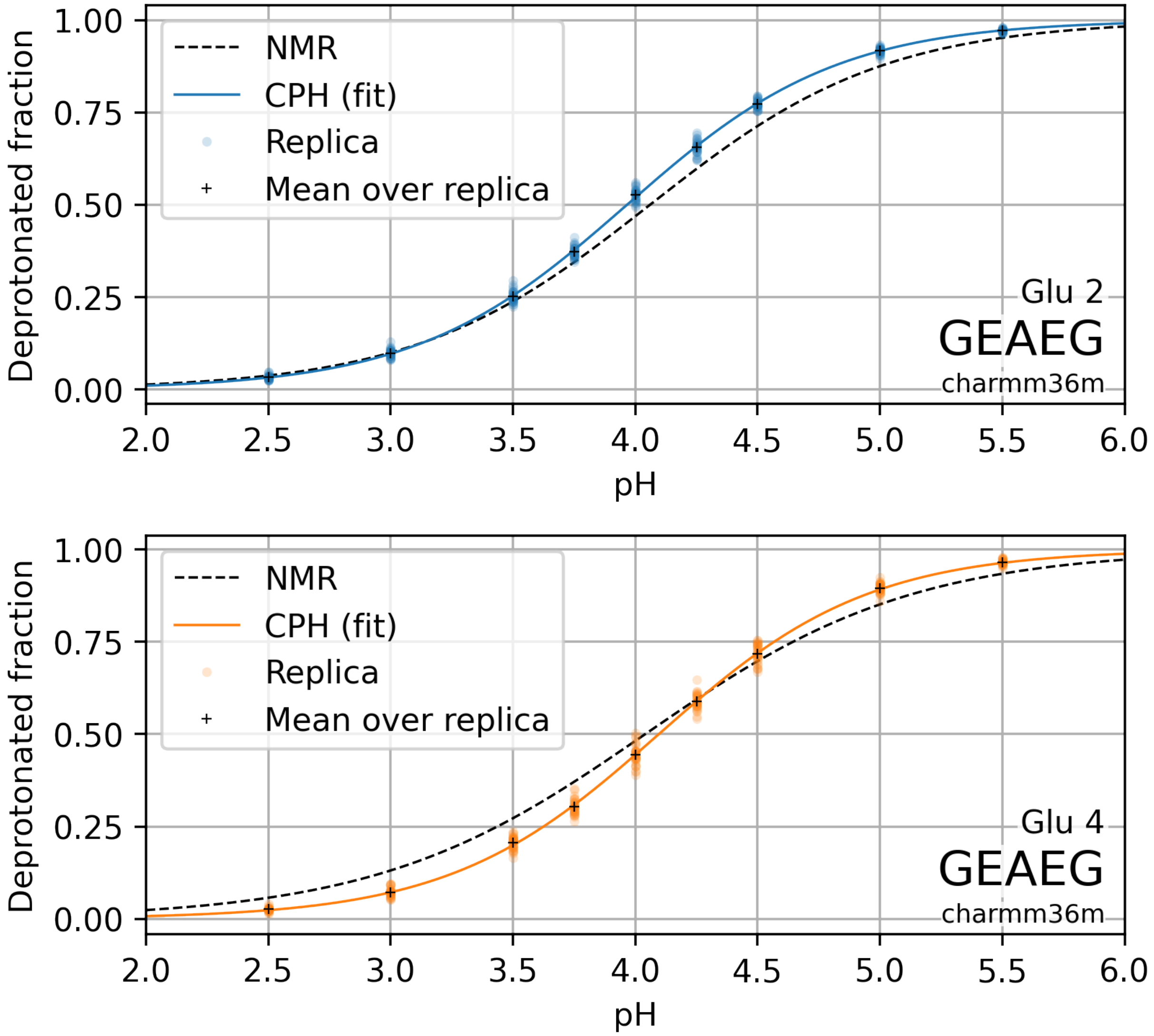

Figure S1: GEAEG titration, CHARMM36m

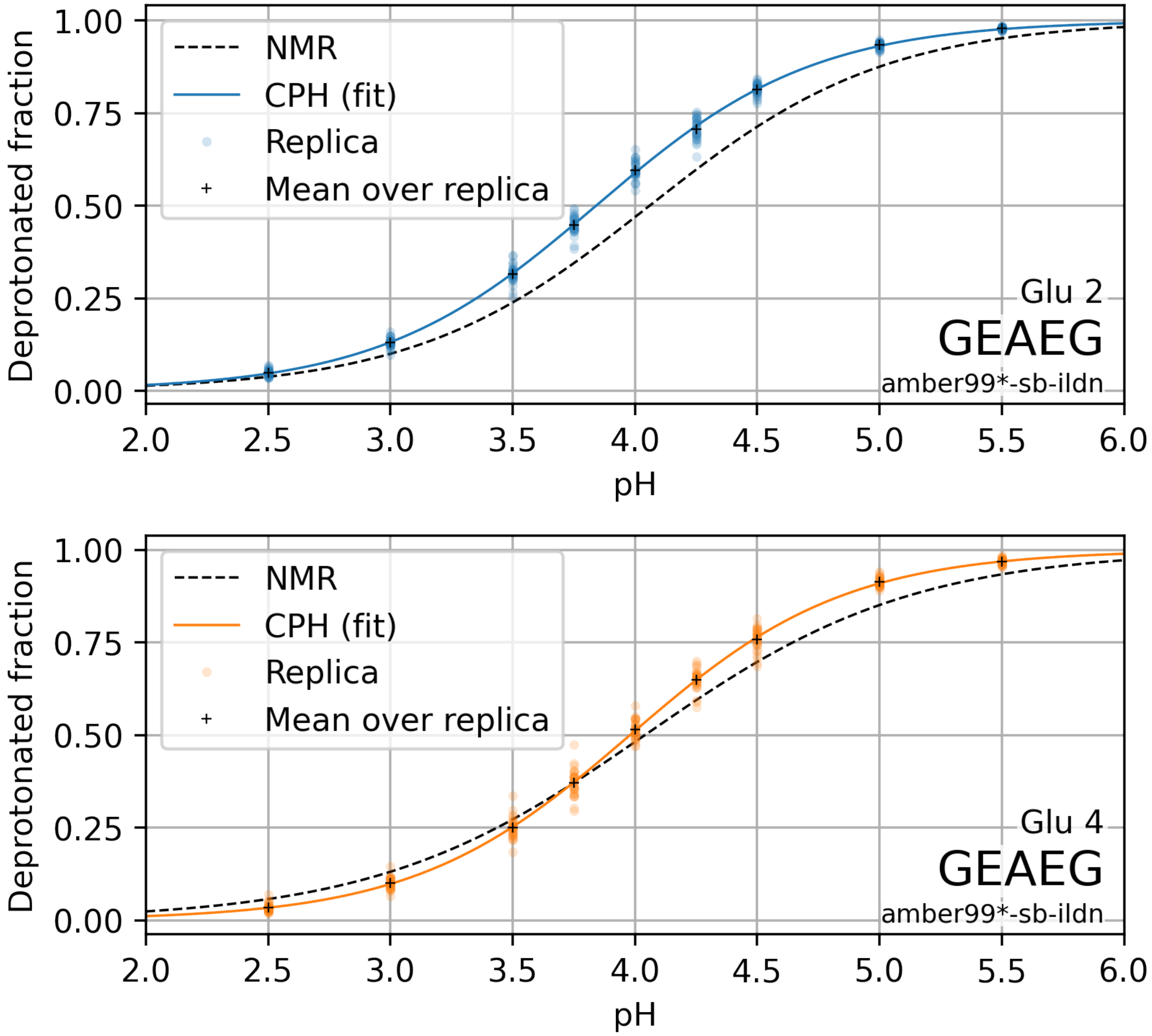

Figure S2: GEAEG titration, Amber99sb*-ILDN

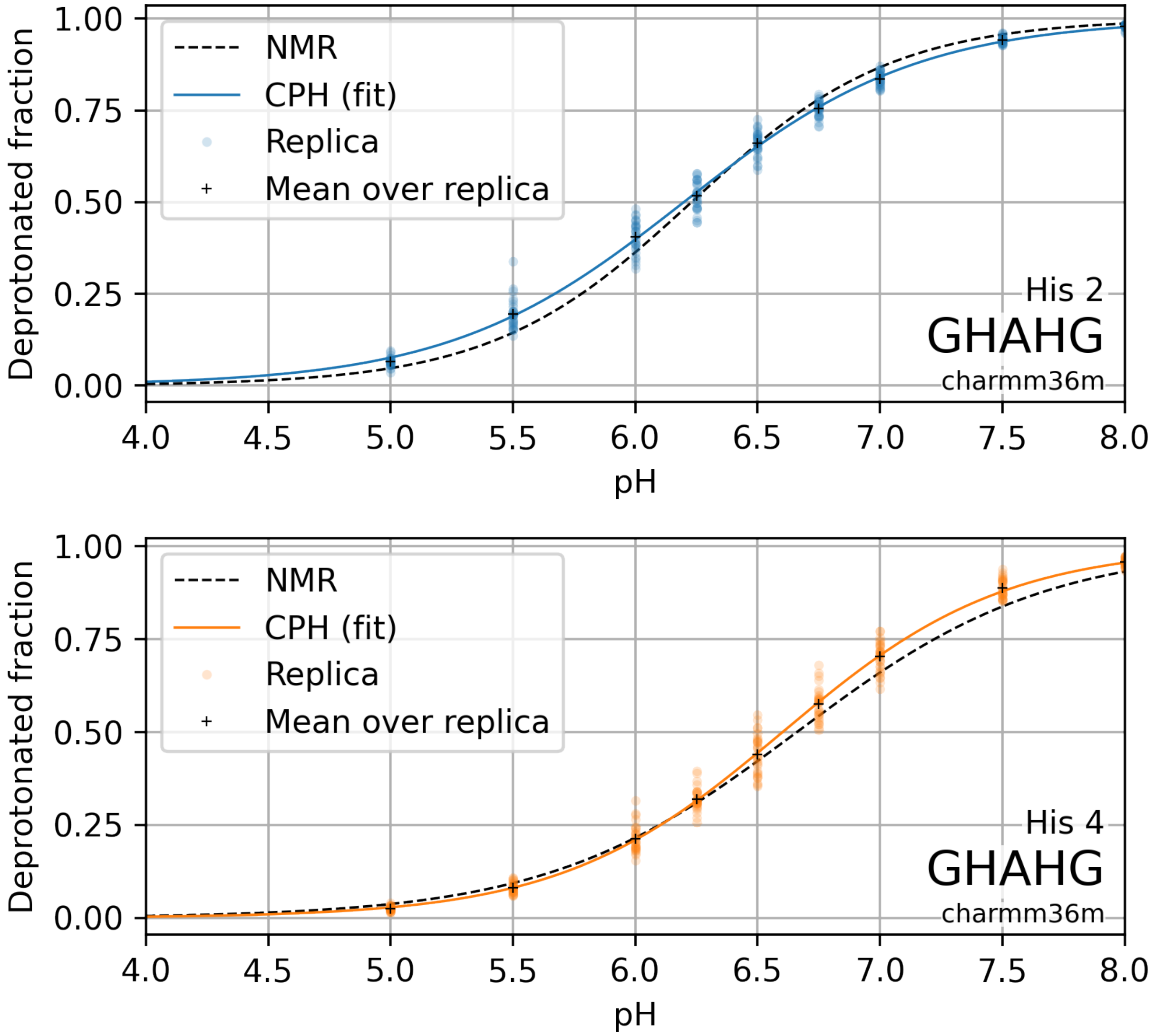

Figure S3: GHAHG titration, CHARMM36m

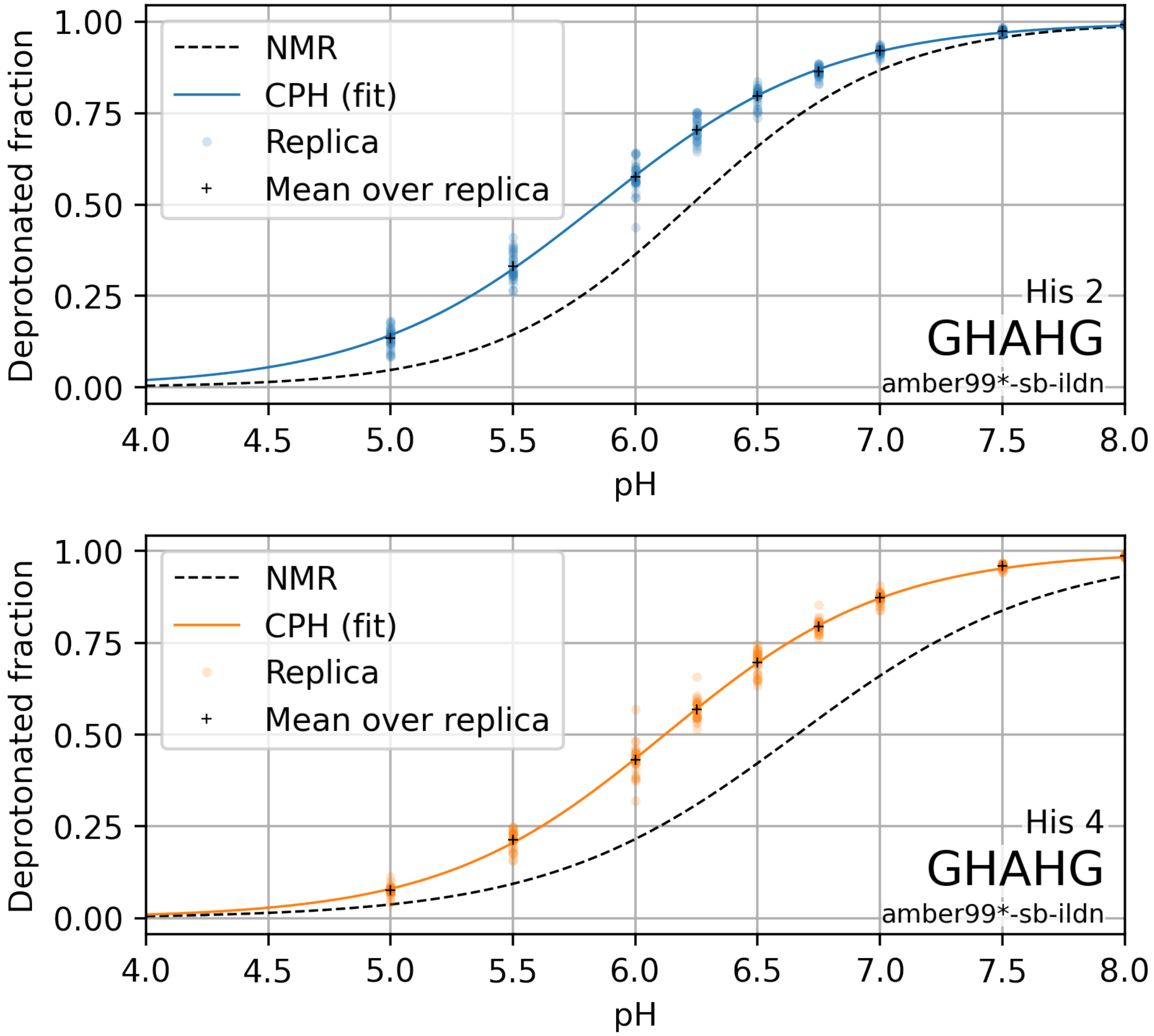

Figure S4: GHAHG titration,Amber99sb*-ILDN

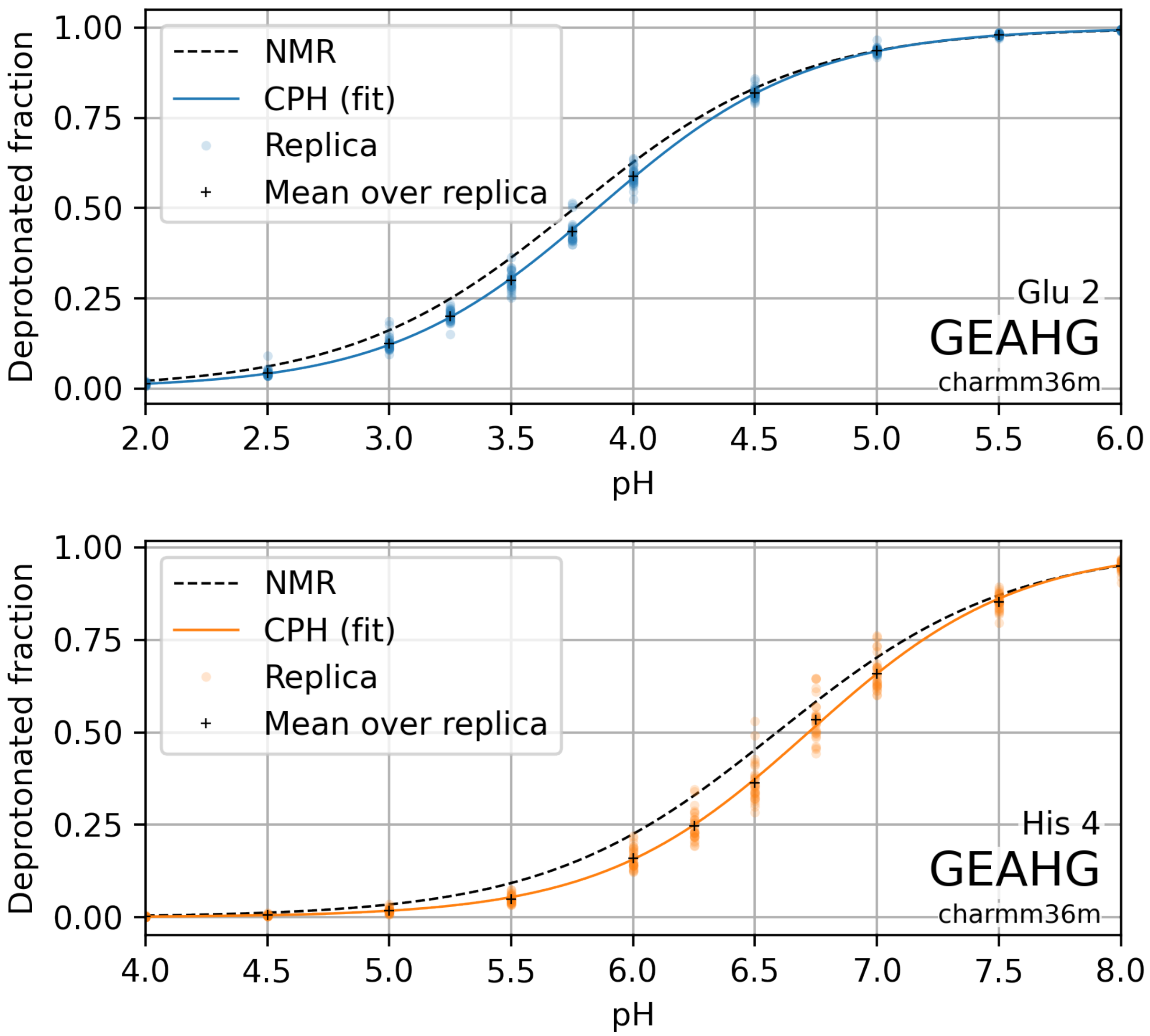

Figure S5: GEAHG titration, CHARMM36m

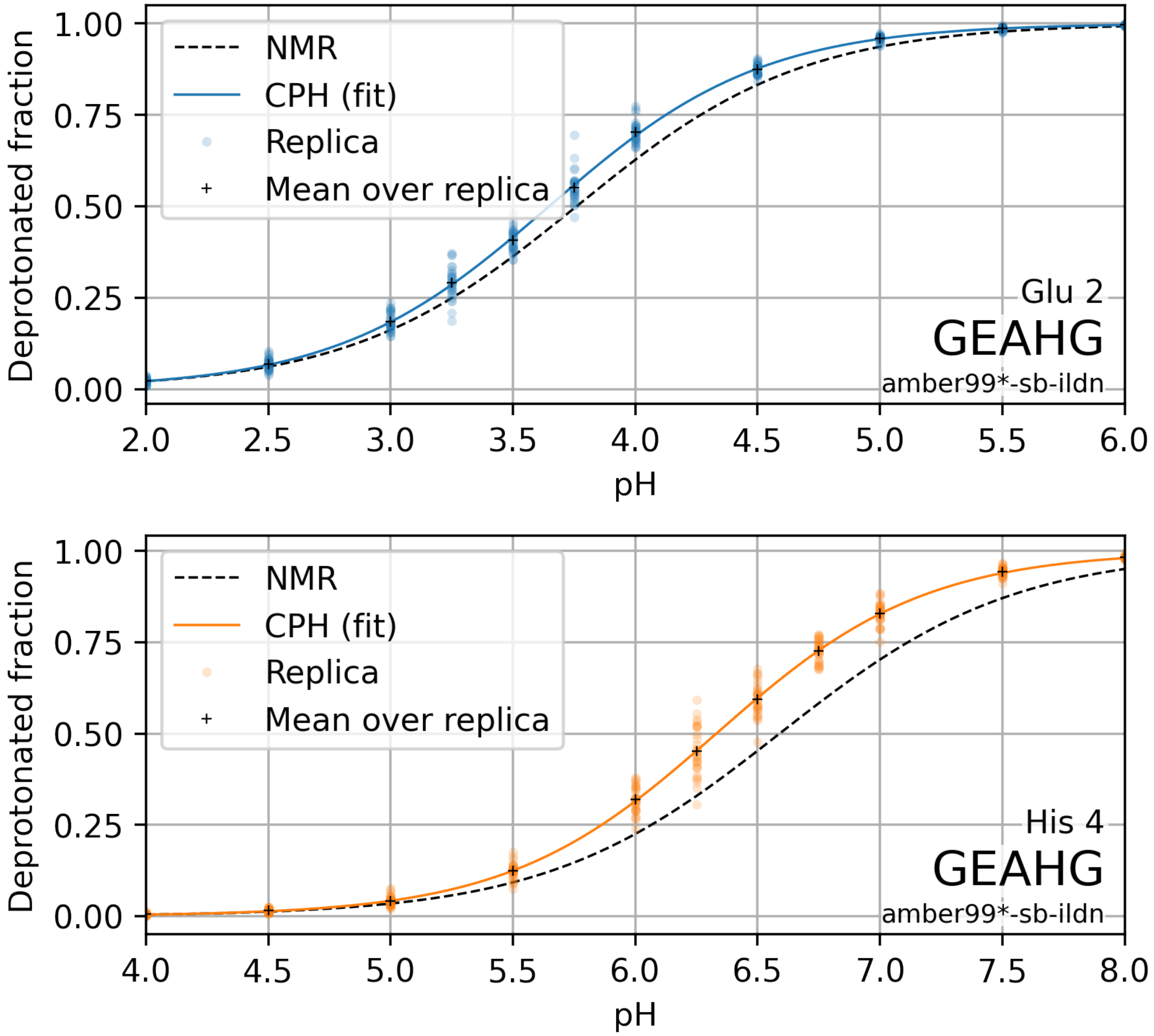

Figure S6: GEAHG titration, Amber99sb*-ILDN

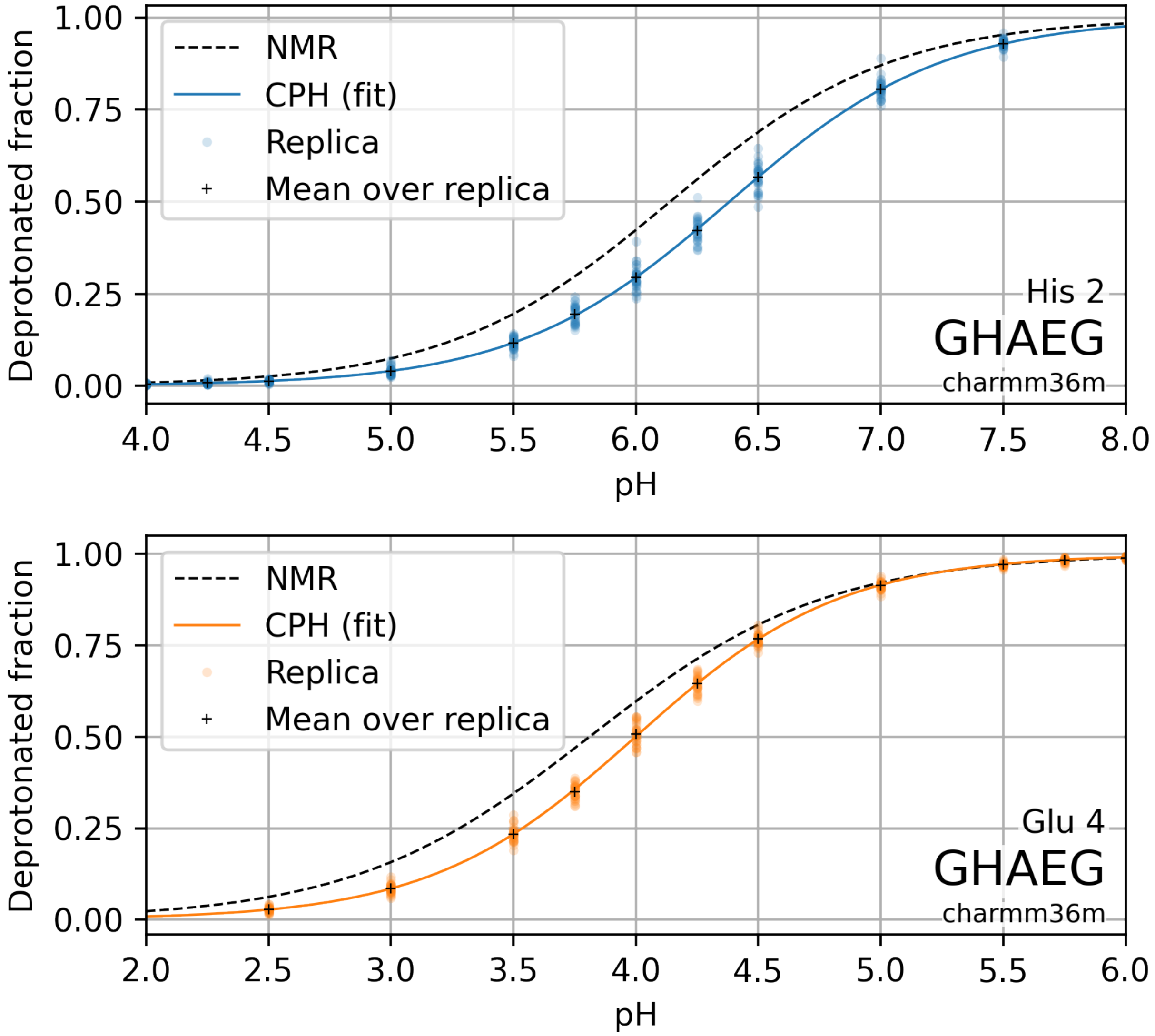

Figure S7: GHAEG titration, CHARMM36m

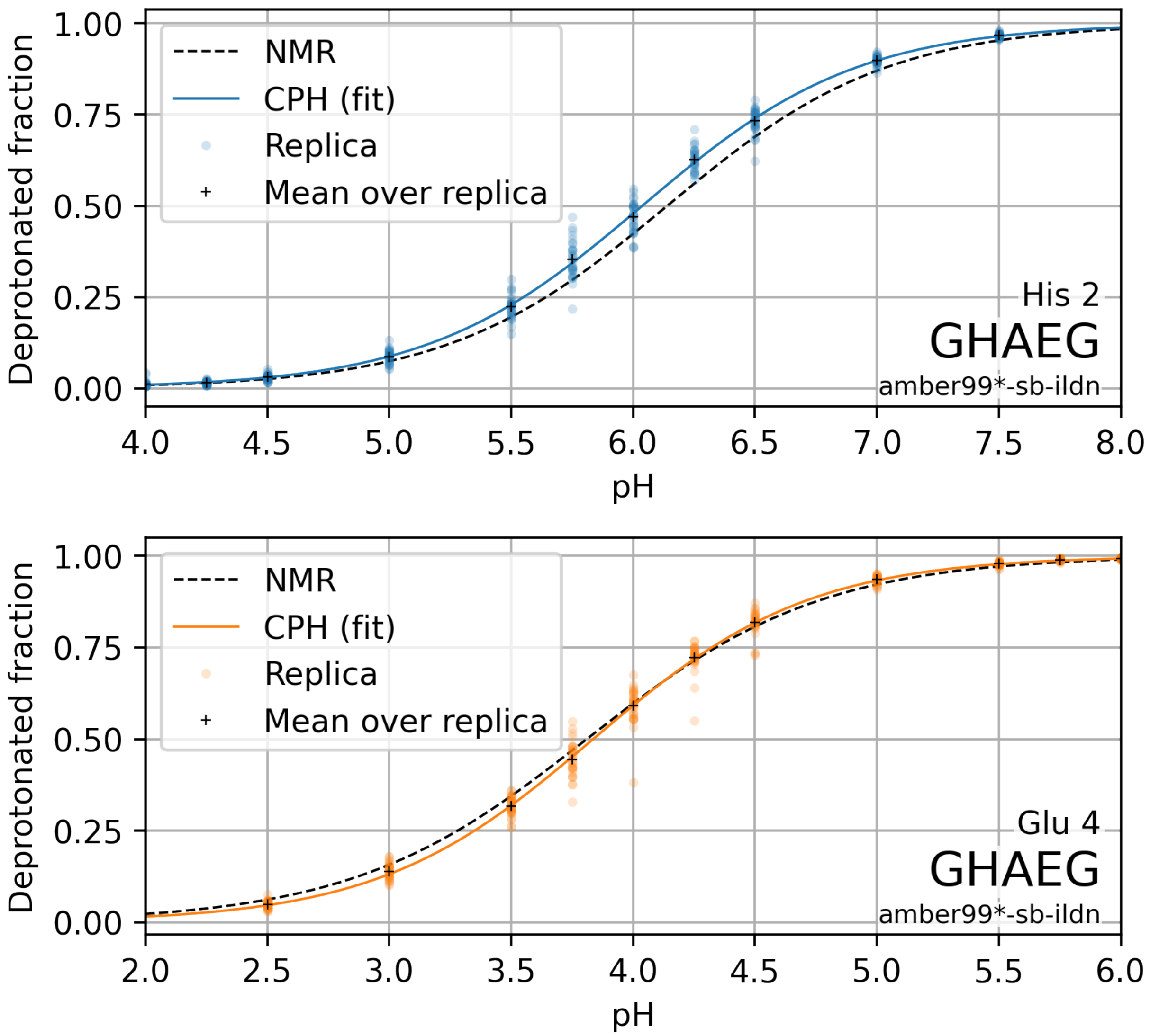

Figure S8: GHAEG titration, Amber99sb*-ILDN

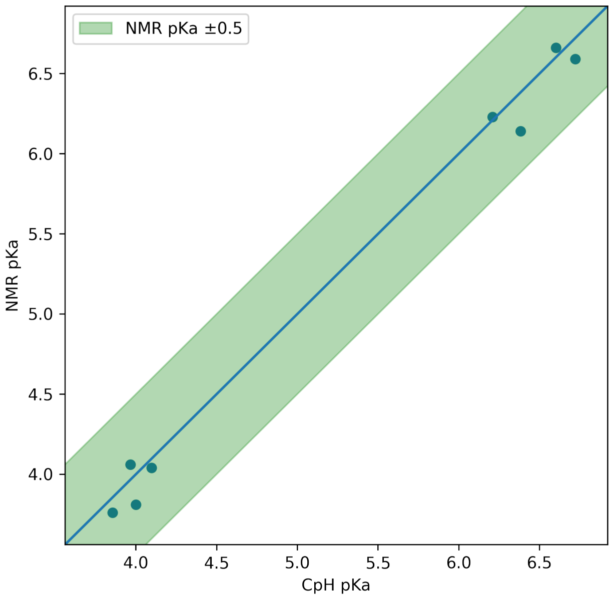

Figure S9: Overall accuracy for pentapeptide titration, CHARMM36m

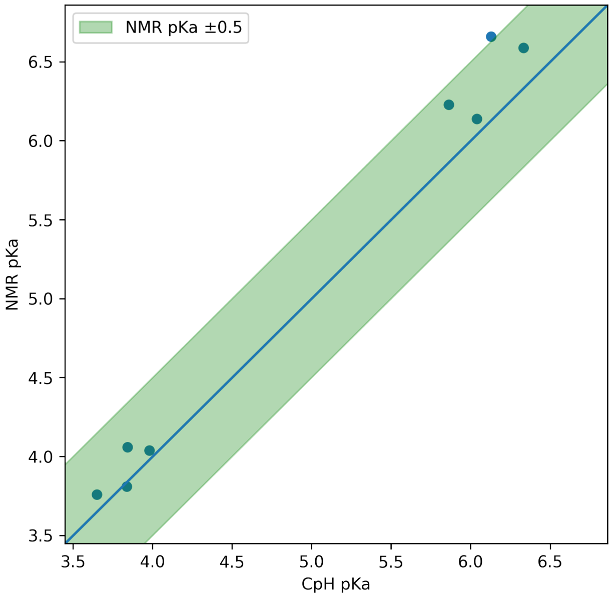

Figure S10: Overall accuracy for pentapeptide titration, Amber99sb*-ILDN

Table 2: Computational titration of pentapeptides: pK_as and Hill coefficients n for Amber99sb*-ILDN compared to NMR-measure pK_a values. CI95 = 95% confidence interval obtained by bootstrapping. NMR single residue pK_a: Glu 4.081, His 6.54. mean absolute error = 0.21, RMSE = 0.26, Max abs. deviation = 0.53.

		Constant pH simulation				Experiment[1]	
Peptide	Residue	pK_a	CI95	n	CI95	pK_a	n
GEAEG	Glu N-ter	3.84	3.83 – 3.85	0.98	0.96 – 0.99	4.06	0.90
	Glu C-ter	3.98	3.97 – 3.99	0.98	0.96 – 1.01	4.04	0.79
GHAHG	His N-ter	5.85	5.83 – 5.87	0.92	0.89 – 0.94	6.23	1.06
	His C-ter	6.12	6.11 – 6.14	0.95	0.93 – 0.97	6.66	0.85
GEAHG	Glu N-ter	3.65	3.63 – 3.66	1.00	0.98 – 1.03	3.76	0.94
	His C-ter	6.33	6.31 – 3.35	1.02	0.99 – 1.05	6.59	0.91
GHAEG	His N-ter	6.04	6.02 – 6.05	0.98	0.95 – 1.01	6.14	0.96
	Glu C-ter	3.84	3.82 – 3.86	0.98	0.95 – 1.01	3.81	0.90

2.2 Cardiotoxin V

The pH point used for the titration were: 1.0, 1.5, 2.0, 2.5, 3.0, 3.5, 4.0, 4.5, 5.0, 5.5, 6.0, 6.5, 7.0, 7.5, 8.0.

Table 3: Computational titration results for cardiotoxin V simulated using CHARMM36m and Amber99sb*-ILDN, with measured NMR pK_a as reference. (x.xx - x.xx): bootstrapped 95th percentile confidence interval. RMSE excluding Asp59 for which only a ceiling value is known. **!**: spread-replica titration

Residue	CHARMM36m	Amber99sb*-ILDN	Exp.[2,3]
His 4	4.33 (4.16 - 4.49) **!**	4.57 (4.41 - 4.71) **!**	5.5
Glu 17	4.10 (4.08 - 4.11)	4.02 (4.02 - 4.03)	4.0
Asp 42	3.85 (3.81 - 3.90)	3.76 (3.75 - 3.78)	3.2
Asp 59	2.17 (2.12 - 2.22)	2.63 (2.58 - 2.68)	$<$2.0
RMSE	0.77	0.63	

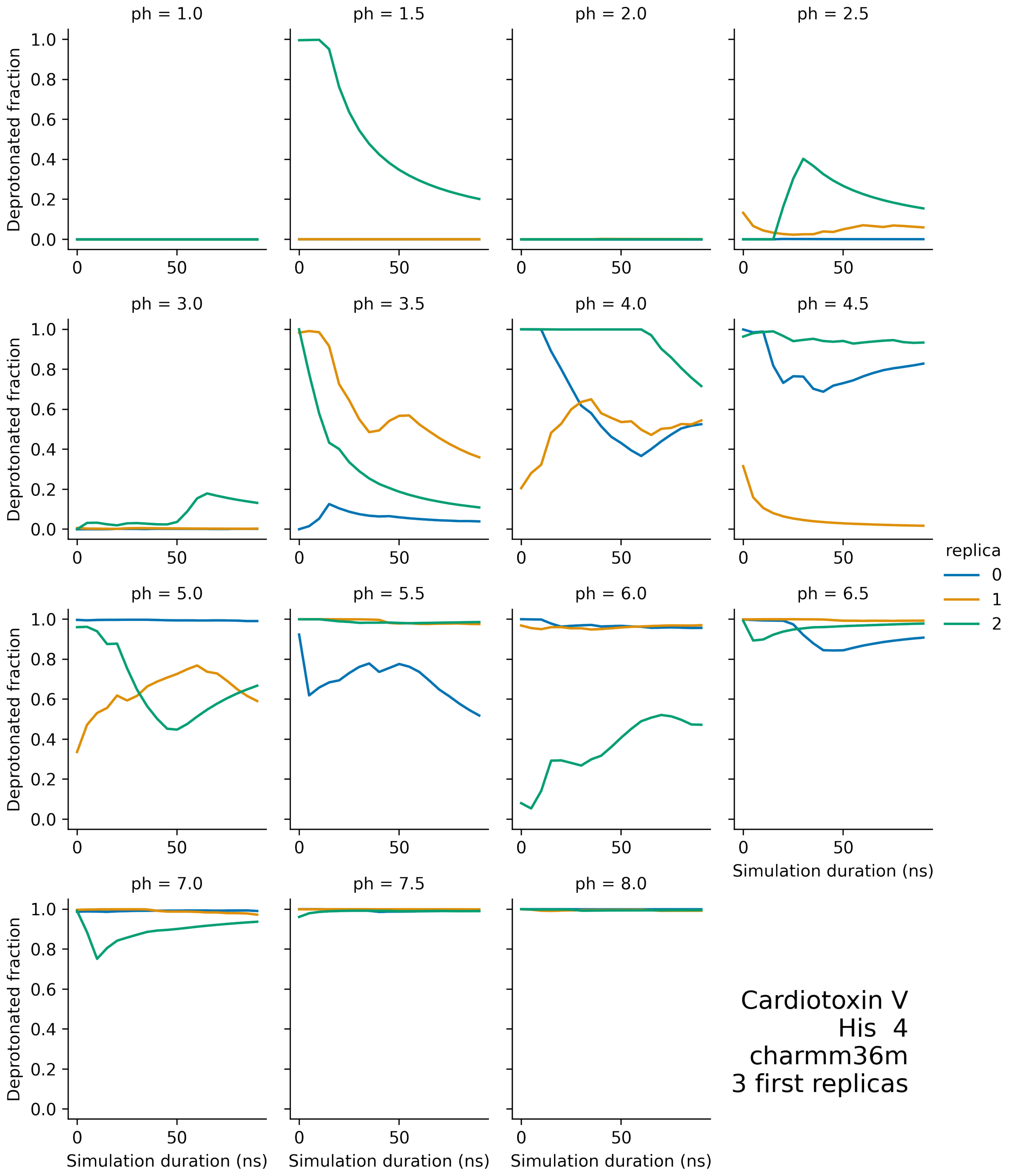

Figure S11: **Titration of His 4**: Deprotonated fraction as a function of simulation duration (ns) over which it is computed, for the three first replica. Computed for duration multiple of 5 ns.

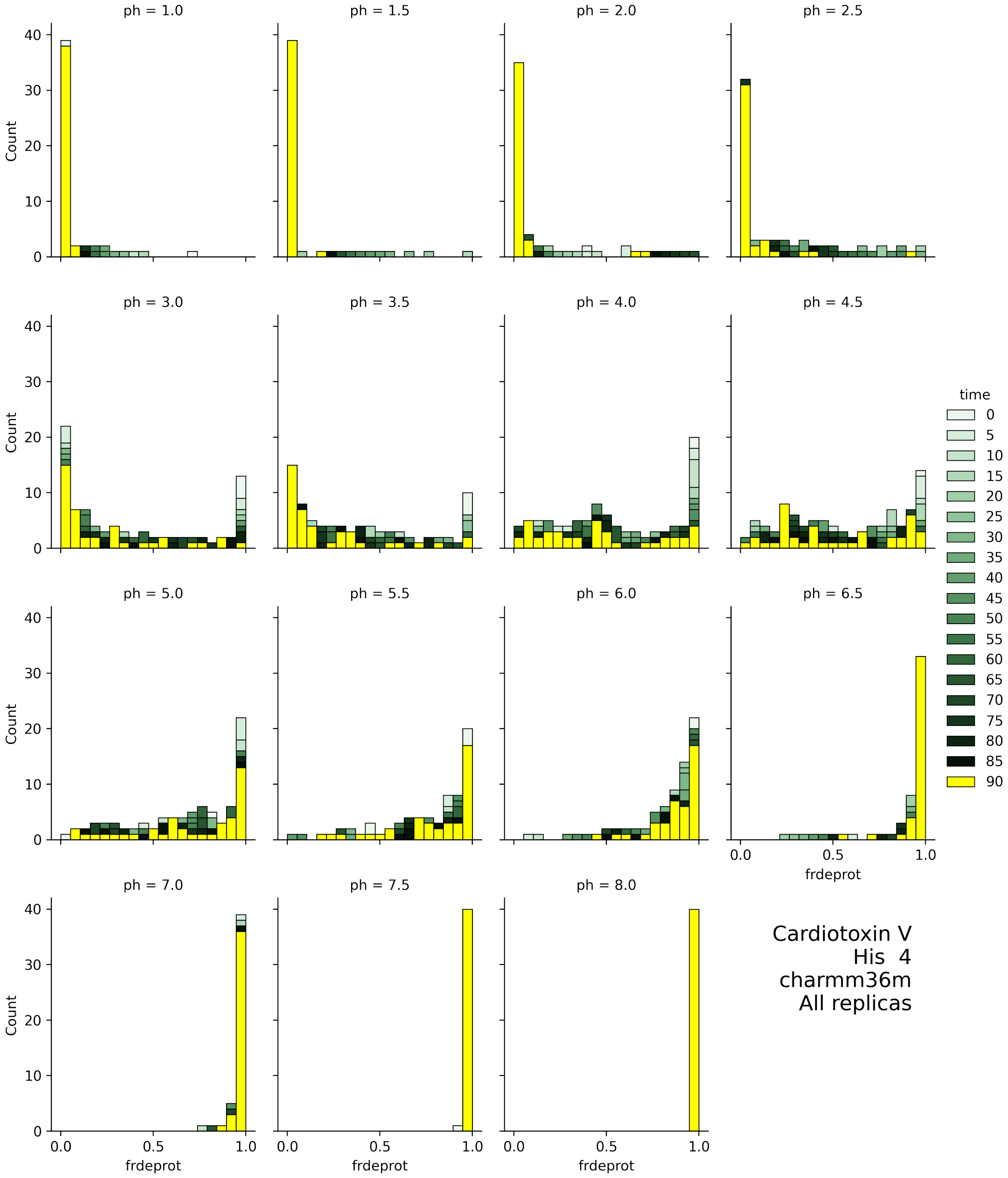

Figure S12: **Titration of His 4**: Histogram of the deprotonated fraction as a function of simulation duration (color coded, ns) over which it is computed, for all replicas. Computed for duration multiple of 5 ns.

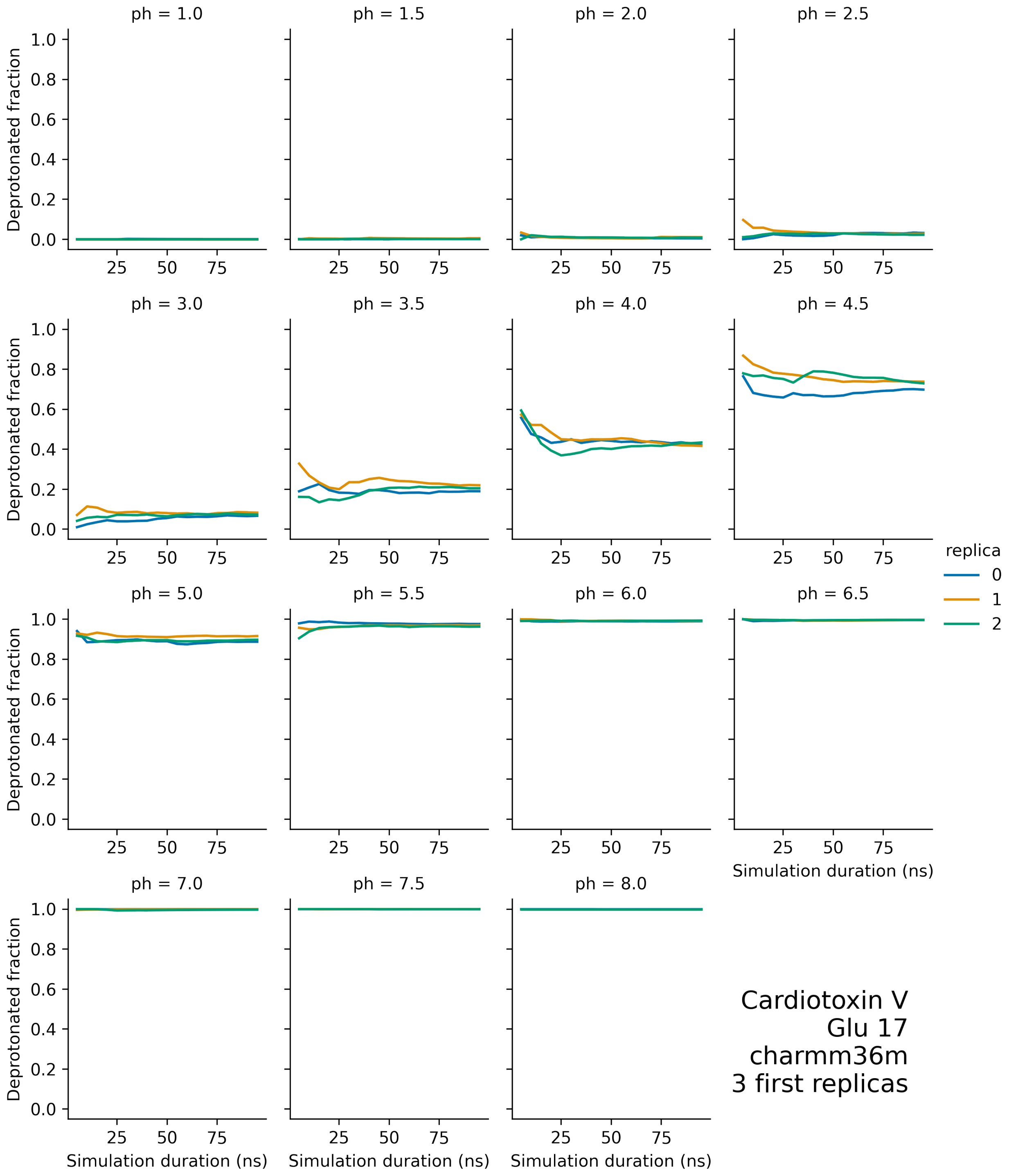

Figure S13: **Titration of Glu 17**: Deprotonated fraction as a function of simulation duration (ns) over which it is computed, for the three first replica. Computed for duration multiple of 5 ns.

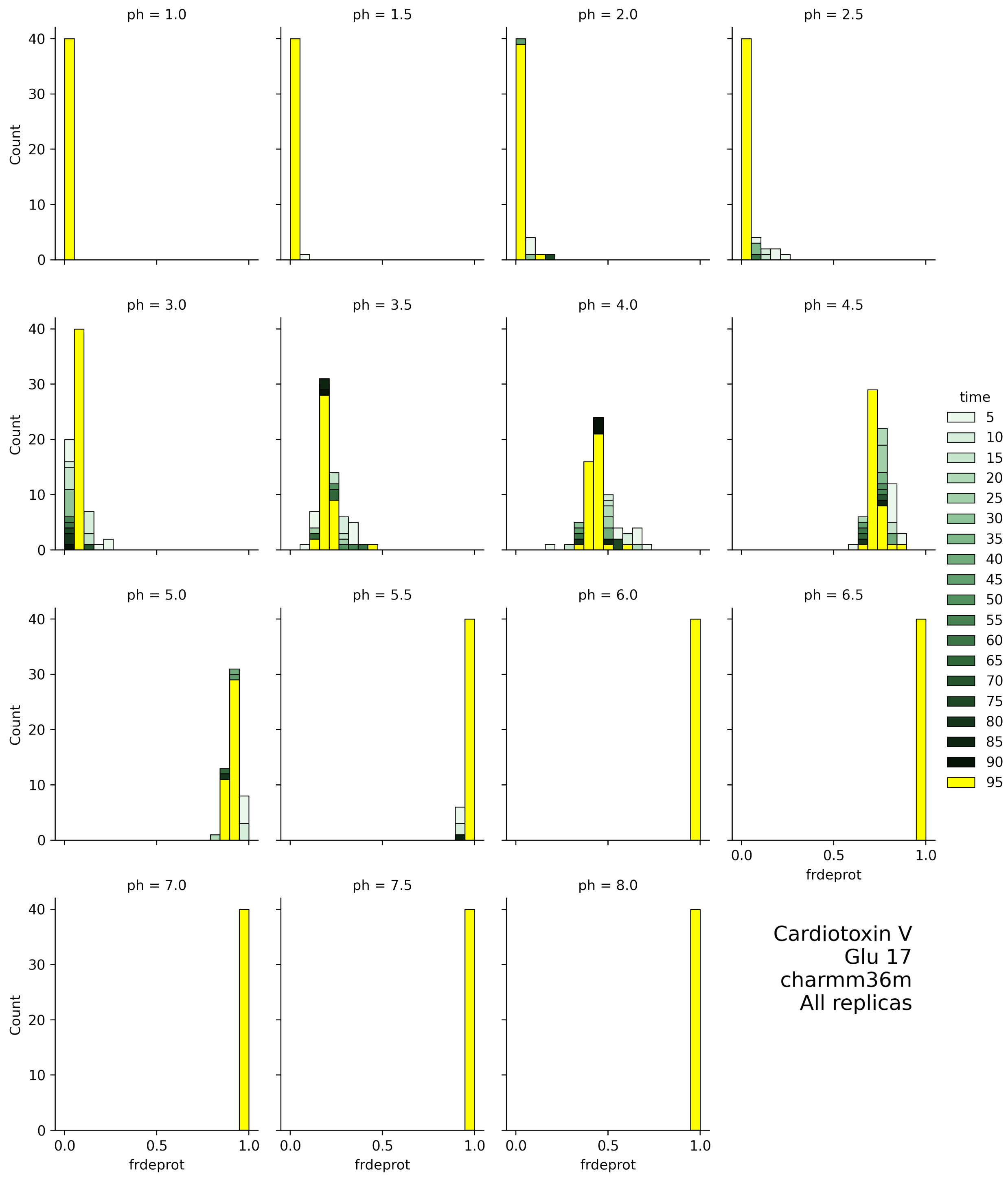

Figure S14: **Titration of Glu 17**: Histogram of the deprotonated fraction as a function of simulation duration (color coded, ns) over which it is computed, for all replicas. Computed for duration multiple of 5 ns.

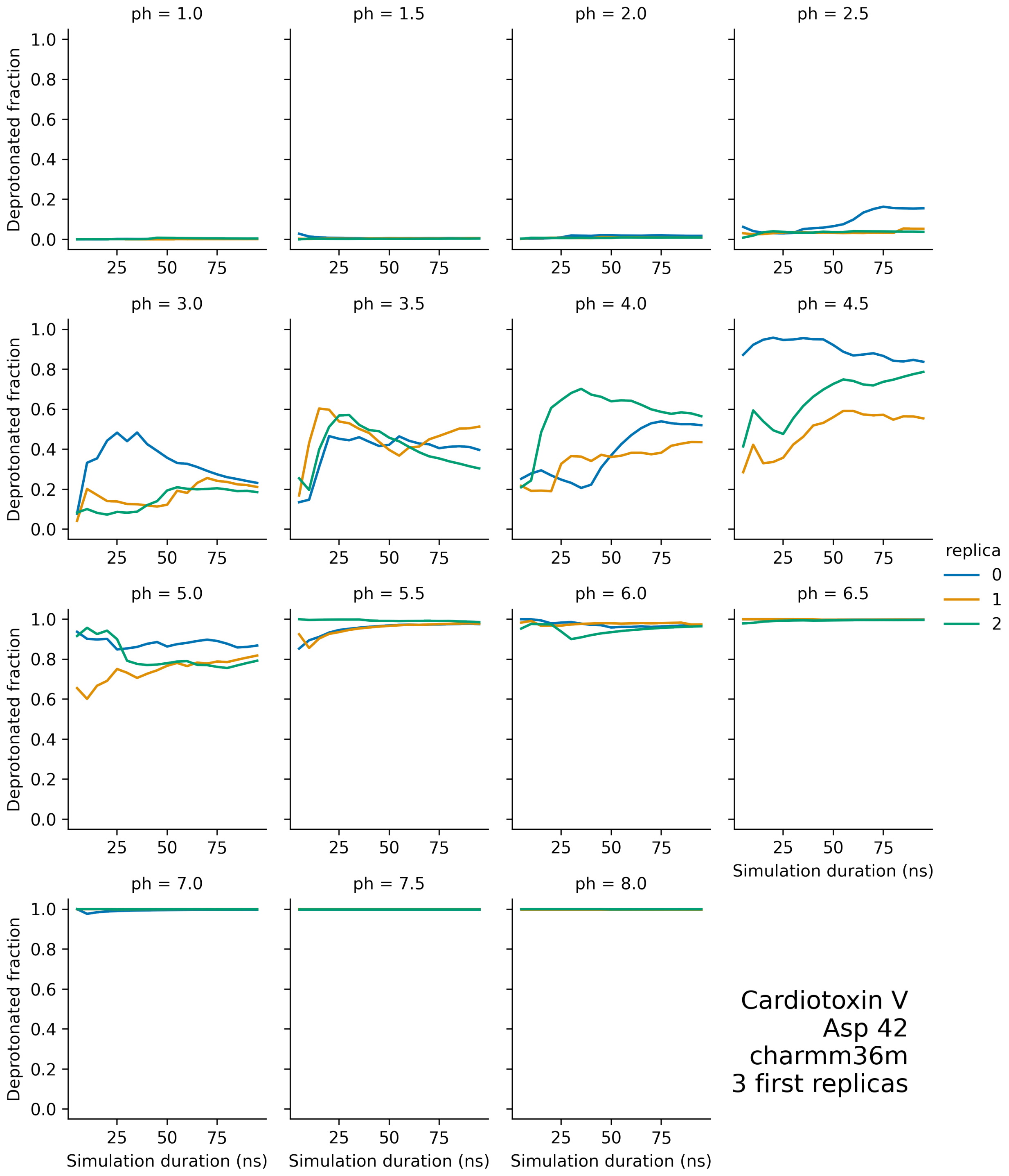

Figure S15: **Titration of Asp 42**: Deprotonated fraction as a function of simulation duration (ns) over which it is computed, for the three first replica. Computed for duration multiple of 5 ns.

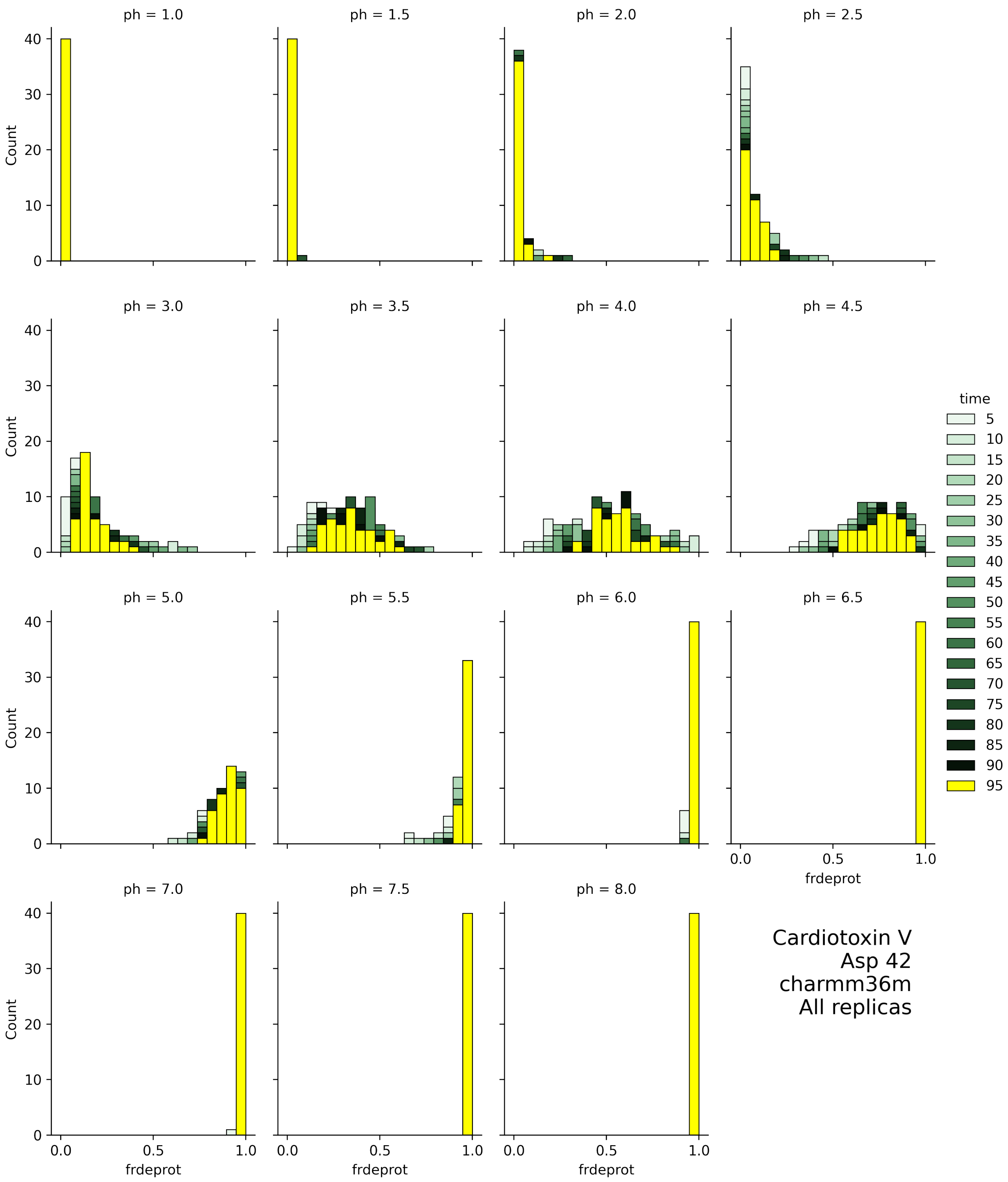

Figure S16: **Titration of Asp 42**: Histogram of the deprotonated fraction as a function of simulation duration (color coded, ns) over which it is computed, for all replicas. Computed for duration multiple of 5 ns.

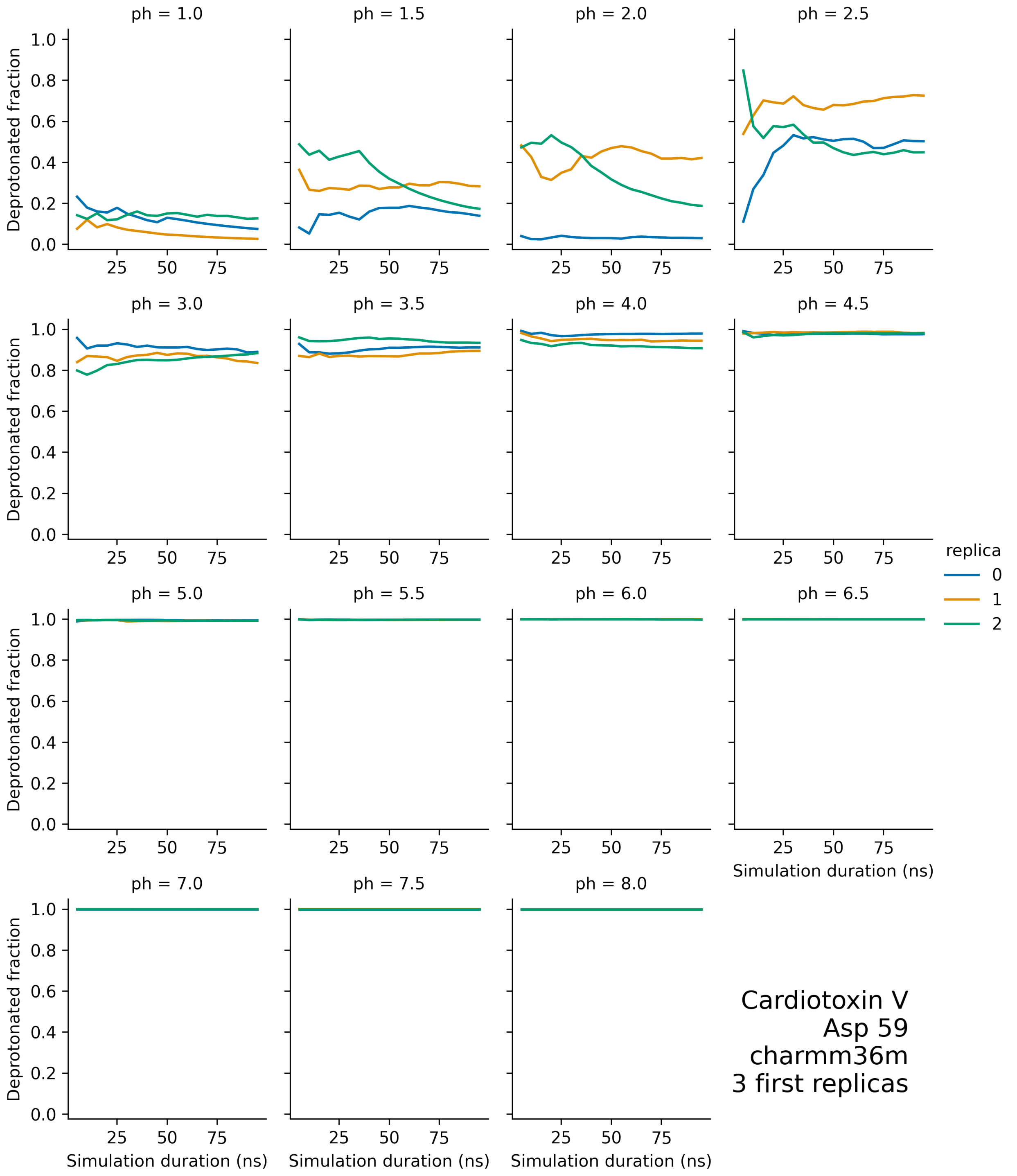

Figure S17: **Titration of Asp 59**: Deprotonated fraction as a function of simulation duration (ns) over which it is computed, for the three first replica. Computed for duration multiple of 5 ns.

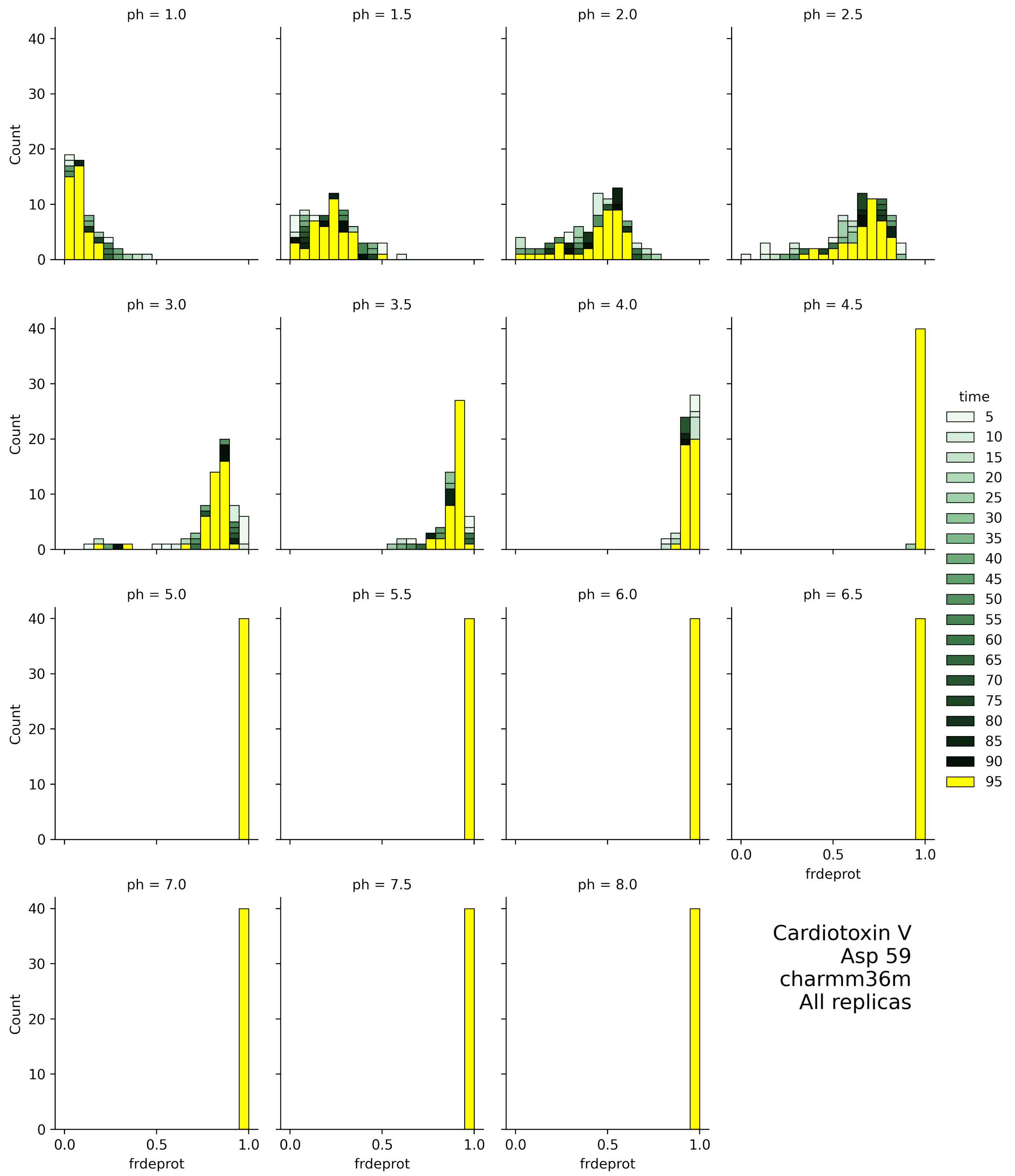

Figure S18: **Titration of Asp 59**: Histogram of the deprotonated fraction as a function of simulation duration (color coded, ns) over which it is computed, for all replicas. Computed for duration multiple of 5 ns.

2.3 Hen Egg White Lysozyme (HEWL)

The following pH value were used for the titration of this protein: -1.0, -0.5, 0.0, 0.5, 1.0, 1.5, 2.0, 2.5, 3.0, 3.5, 4.0, 4.5, 5.0, 5.5, 6.0, 6.5, 7.0, 7.5, 8.0, 8.5, 9.0.

Table 4: Computational titration results for lysozyme (40 replicas per pH point, 75 ns per replica) (x.xx - x.xx): Bootstrapped 95th percentile confidence intervals. **!**: spread-replica titration

Residue	CPH CHARMM36m	CPH Amber99sb*-ILDN	Exp. NMR[4]
Glu 7	3.31 (3.28 - 3.33)	3.00 (2.97 - 3.02)	2.6 $\pm$0.2
His 15	4.57 (4.54 - 4.60)	3.03 (2.97 - 3.10) **!**	5.5 $\pm$0.2
Asp 18	3.10 (3.03 - 3.17)	2.65 (2.61 - 2.69)	2.8 $\pm$0.3
Glu 35	7.41 (7.26 - 7.56) **!**	6.21 (6.10 - 6.32) **!**	6.1 $\pm$0.4
Asp 48	0.37 (0.27 - 0.48) **!**	1.71 (1.65 - 1.77) **!**	1.4 $\pm$0.2
Asp 52	5.05 (5.00 - 5.12)	4.02 (3.98 - 4.06)	3.6 $\pm$0.3
Asp 66	1.28 (1.10 - 1.46) **!**	0.65 (0.50 - 0.81) **!**	1.2 $\pm$0.2
Asp 87	2.20 (2.14 - 2.27)	1.74 (1.70 - 1.79)	2.2 $\pm$0.1
Asp 101	4.42 (4.38 - 4.48)	3.59 (3.52 - 3.66)	4.5 $\pm$0.1
Asp 119	2.55 (2.51 - 2.59)	2.96 (2.94 - 2.99)	3.5 $\pm$0.3
RMSE	0.85	0.90	

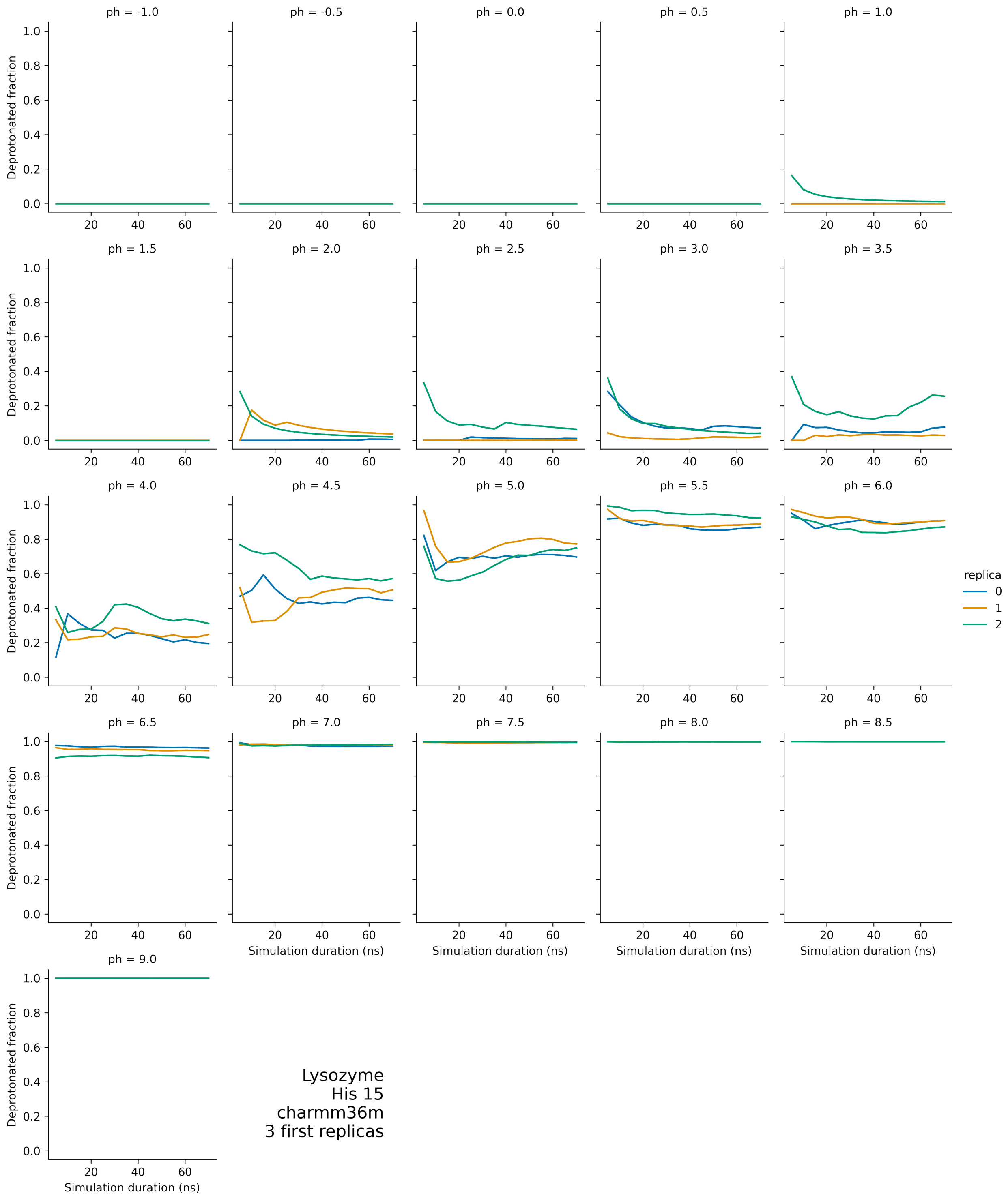

Figure S19: **Titration of His 15**: Deprotonated fraction as a function of simulation duration (ns) over which it is computed, for the three first replica. Computed for duration multiple of 5 ns.

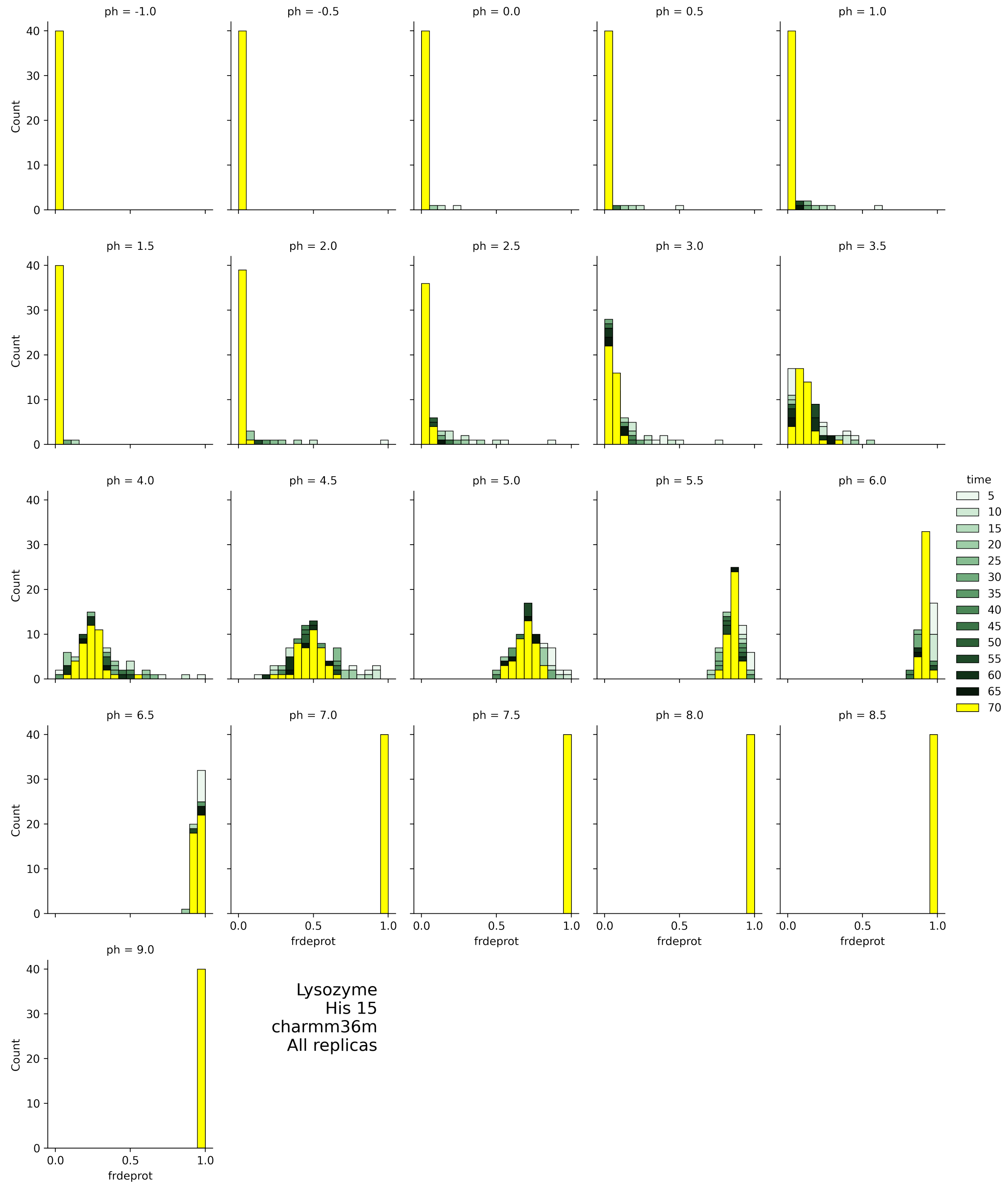

Figure S20: **Titration of His 15**: Histogram of the deprotonated fraction as a function of simulation duration (color coded, ns) over which it is computed, for all replicas. Computed for duration multiple of 5 ns.

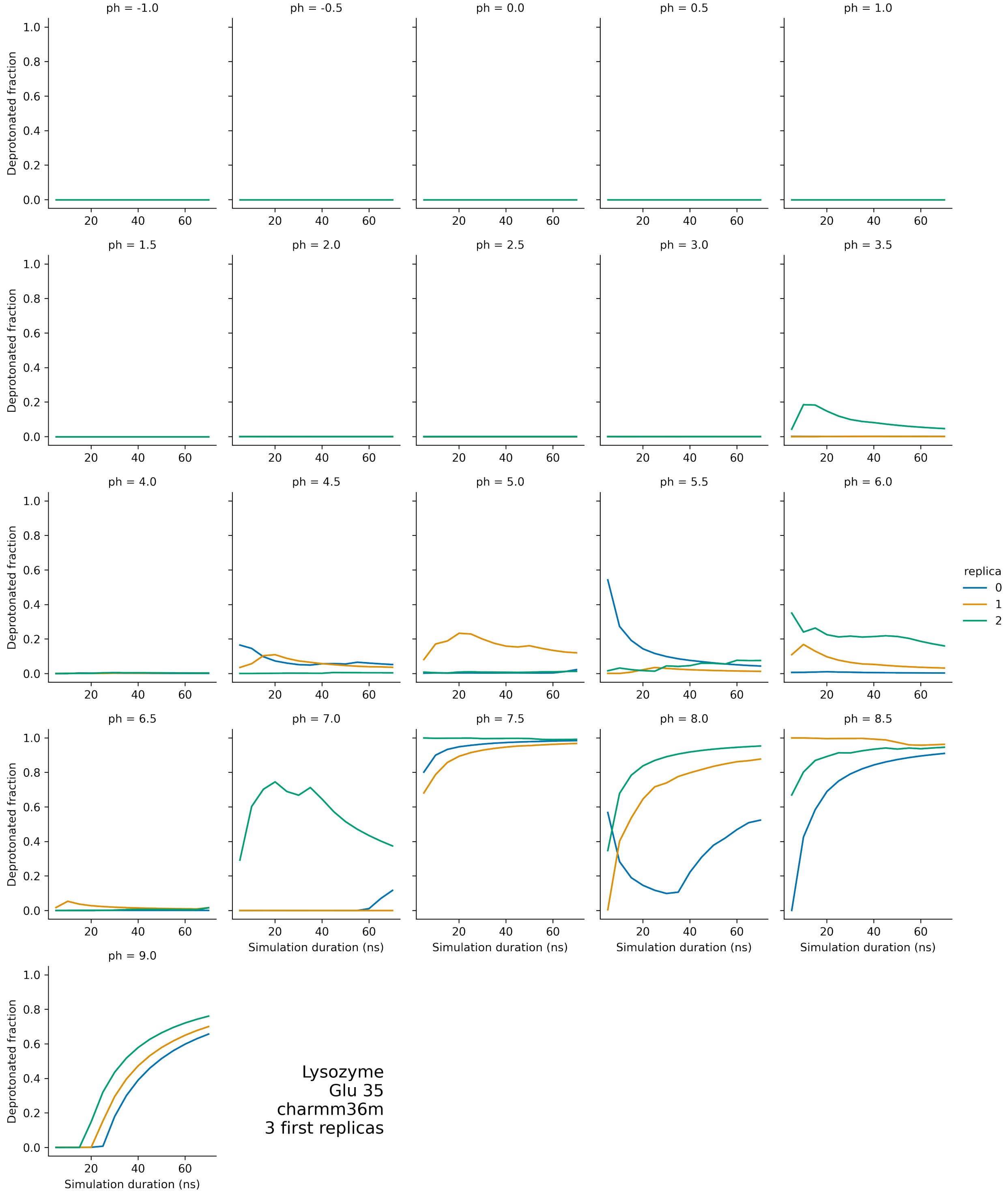

Figure S21: **Titration of Glu 35**: Deprotonated fraction as a function of simulation duration (ns) over which it is computed, for the three first replica. Computed for duration multiple of 5 ns.

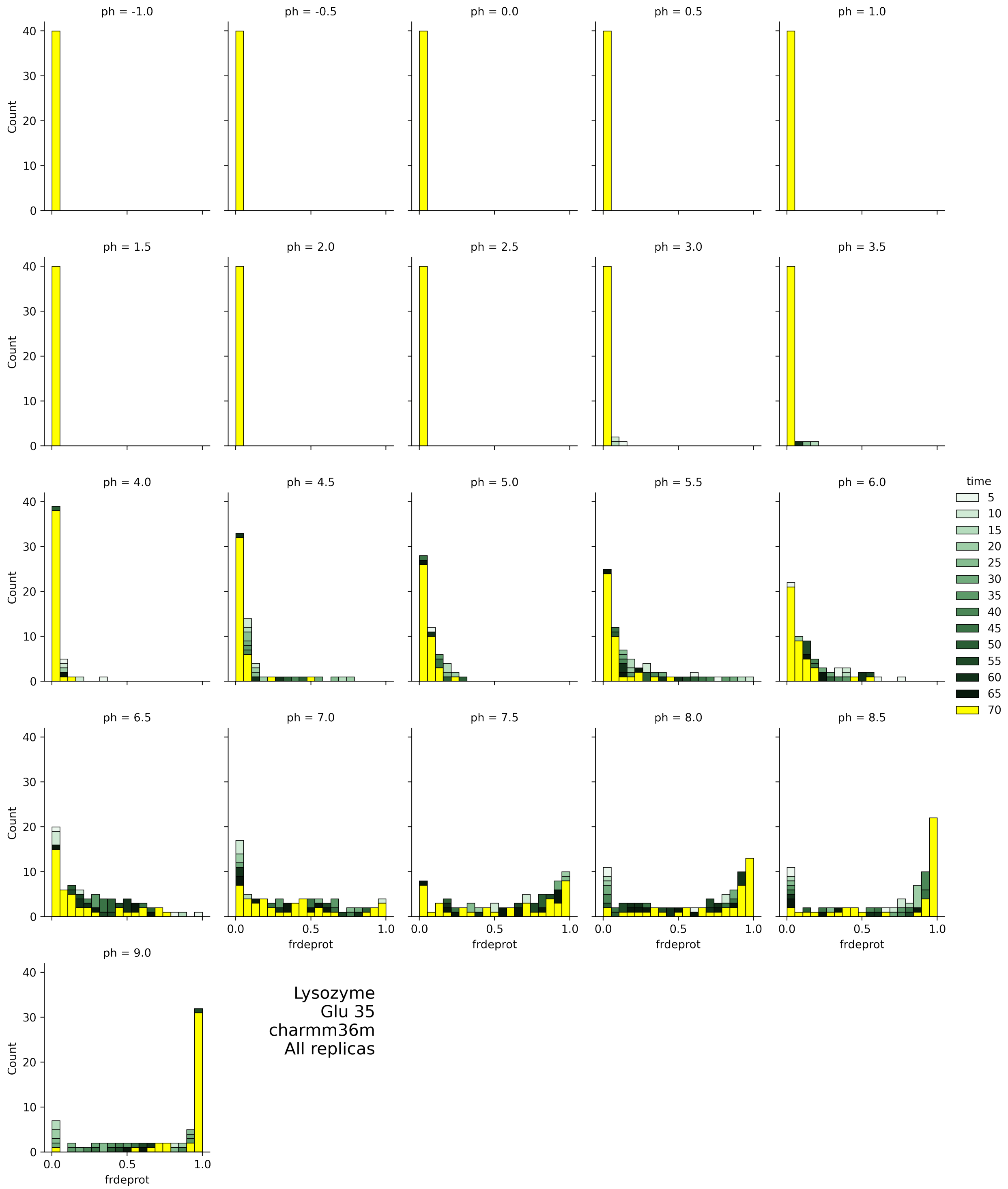

Figure S22: **Titration of Glu 35**: Histogram of the deprotonated fraction as a function of simulation duration (color coded, ns) over which it is computed, for all replicas. Computed for duration multiple of 5 ns.

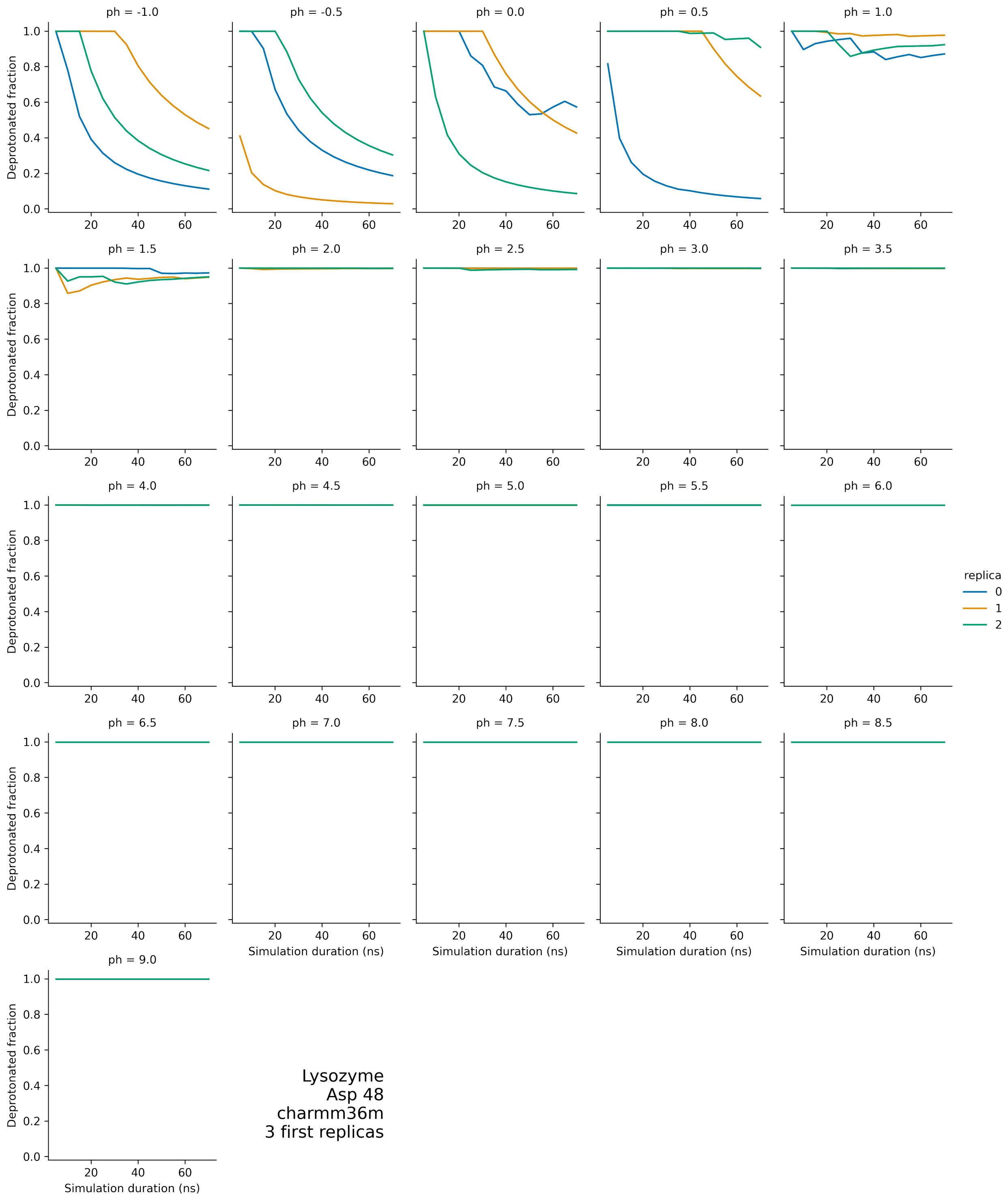

Figure S23: **Titration of Asp 48**: Deprotonated fraction as a function of simulation duration (ns) over which it is computed, for the three first replica. Computed for duration multiple of 5 ns.

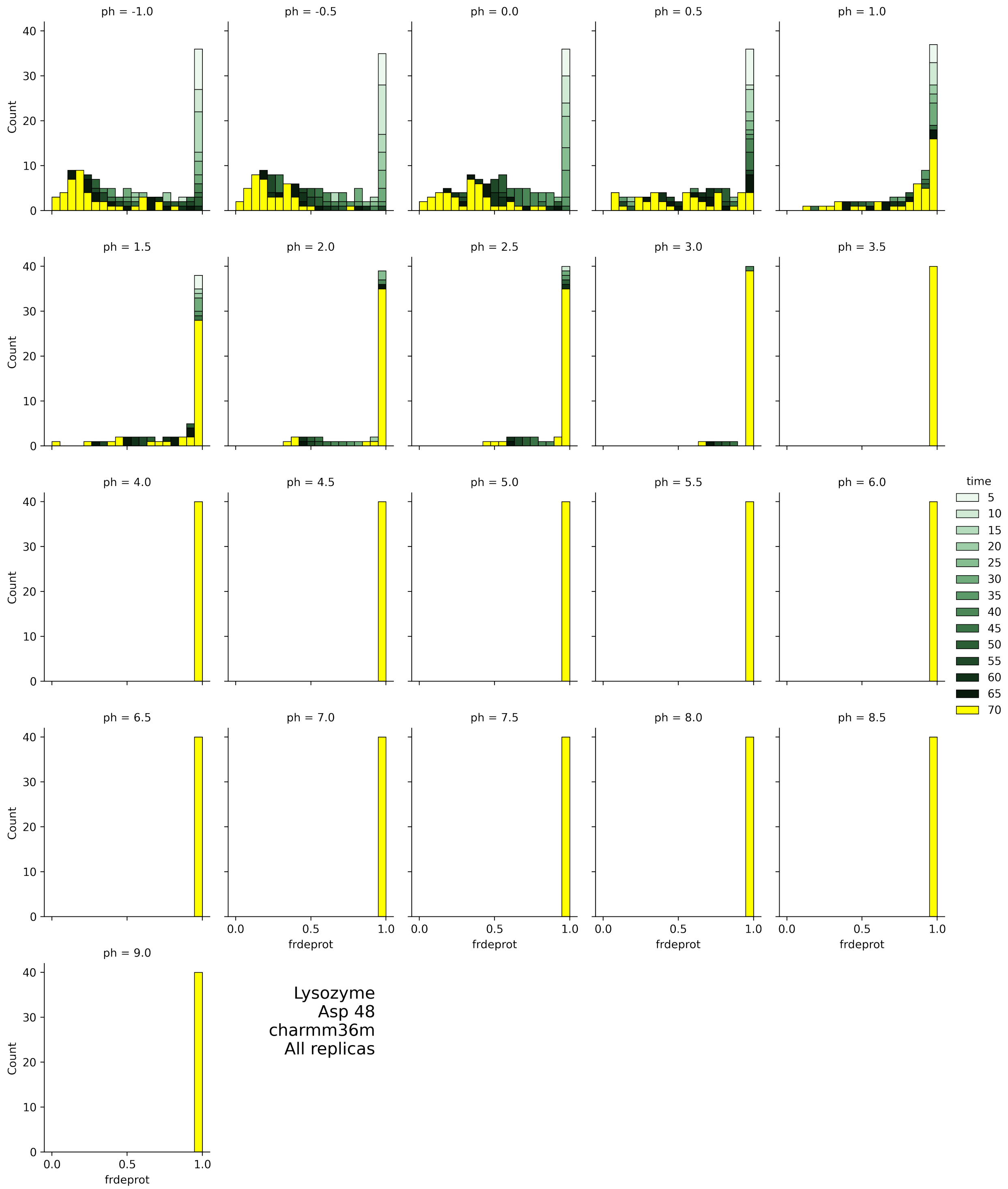

Figure S24: **Titration of Asp 48**: Histogram of the deprotonated fraction as a function of simulation duration (color coded, ns) over which it is computed, for all replicas. Computed for duration multiple of 5 ns.

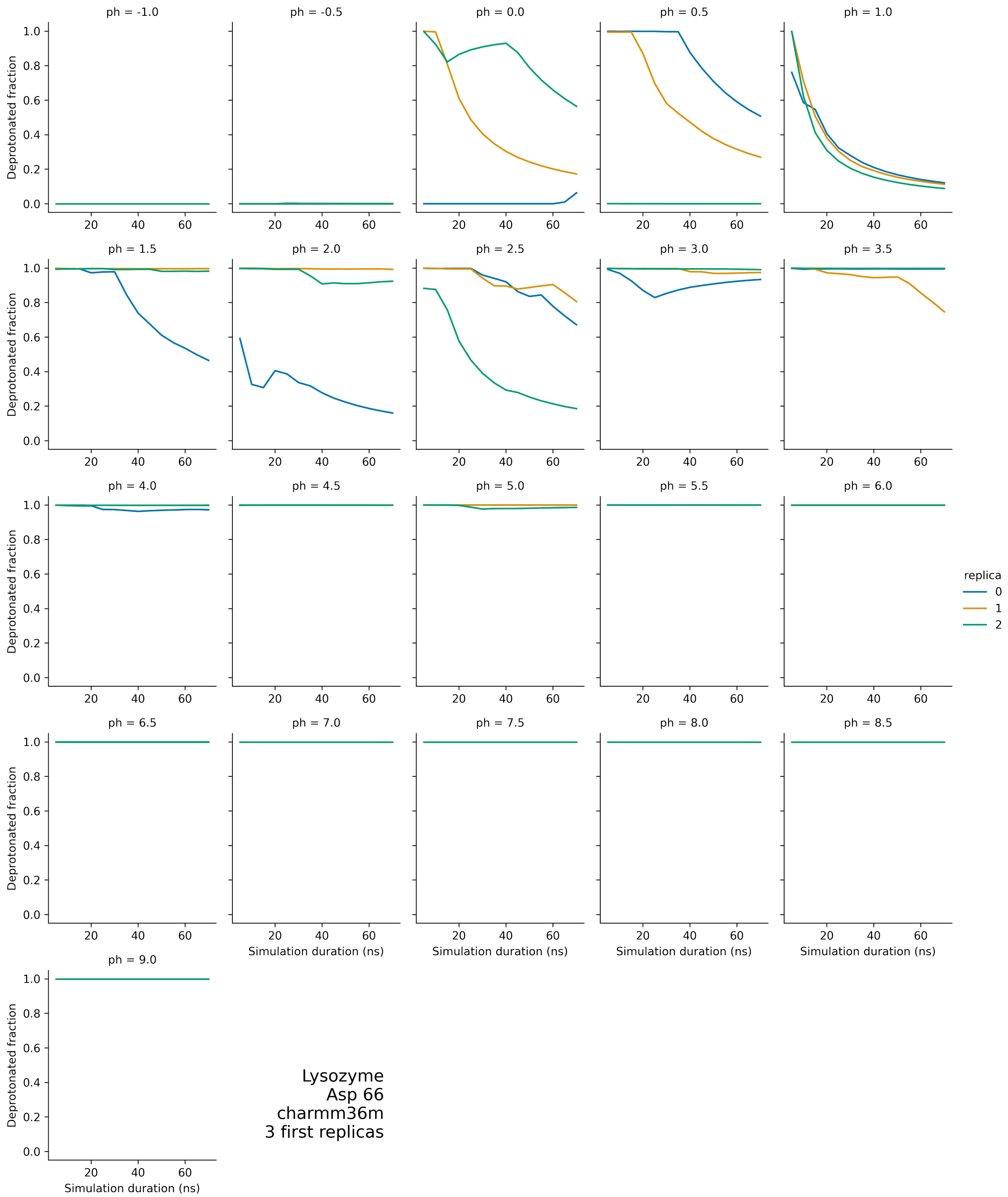

Figure S25: **Titration of Asp 66**: Deprotonated fraction as a function of simulation duration (ns) over which it is computed, for the three first replica. Computed for duration multiple of 5 ns.

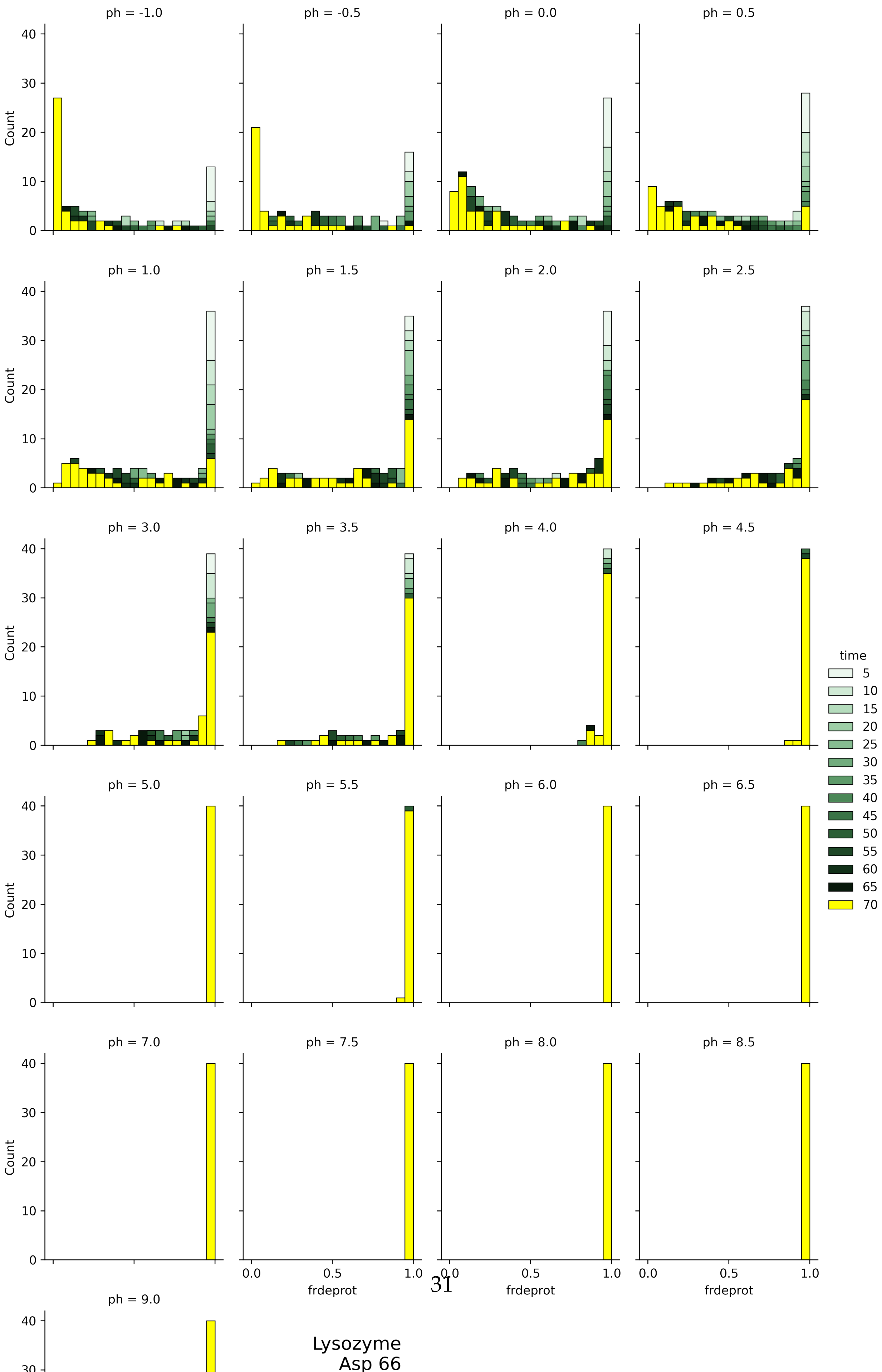
ph = -1.0
ph = -0.5
ph = 0.0
ph = 0.5
ph = 1.0
ph = 1.5
ph = 2.0
ph = 2.5
ph = 3.0
ph = 3.5
ph = 4.0
ph = 4.5
ph = 5.0
ph = 5.5
ph = 6.0
ph = 6.5
ph = 7.0
ph = 7.5
ph = 8.0
ph = 8.5
ph = 9.0
Count
frdeprot
0.0
0.5
1.0
0
10
20
30
40
time
5
10
15
20
25
30
35
40
45
50
55
60
65
70
Lysozyme
Asp 66

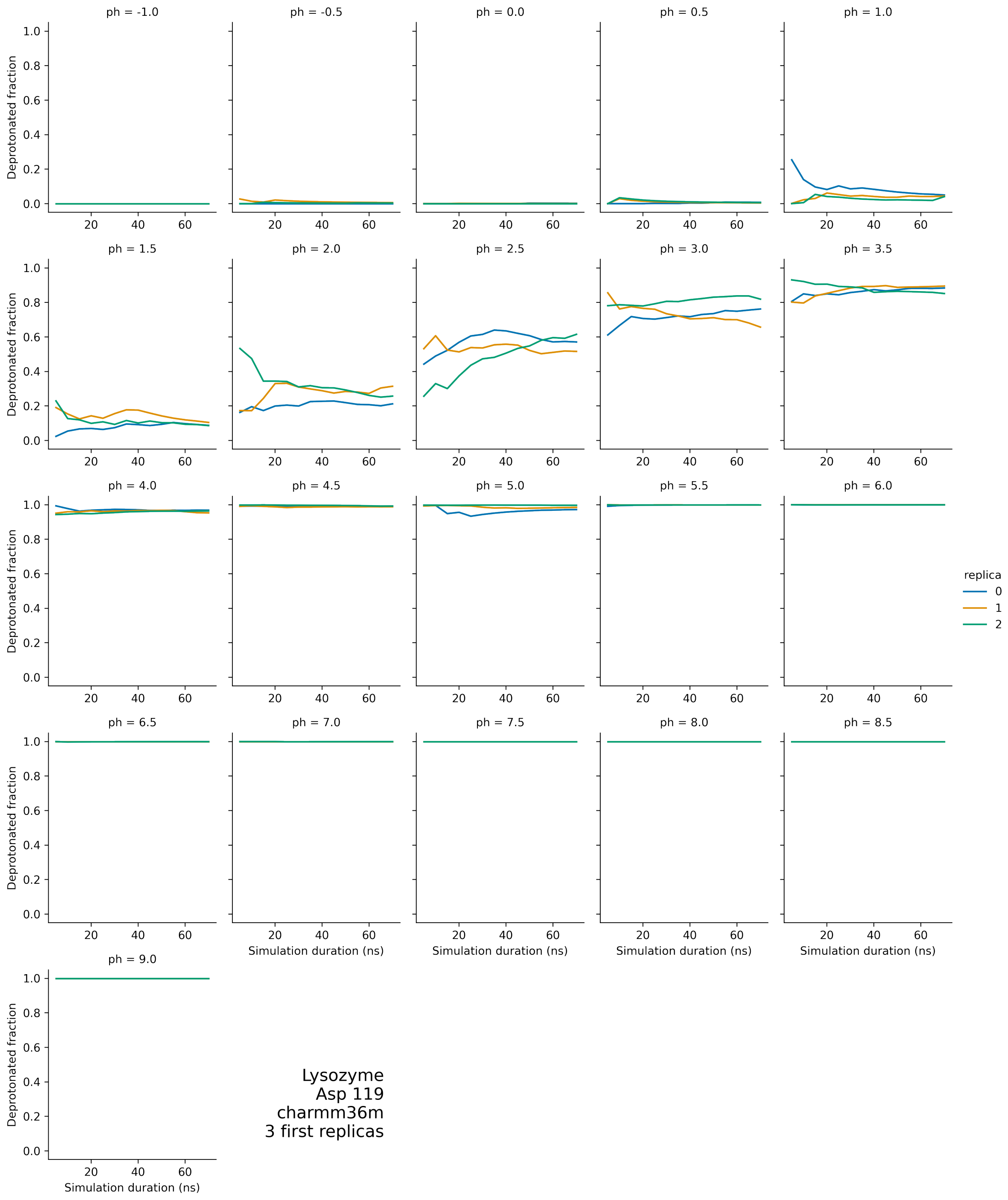

Figure S27: **Titration of Asp 119**: Deprotonated fraction as a function of simulation duration (ns) over which it is computed, for the three first replica. Computed for duration multiple of 5 ns.

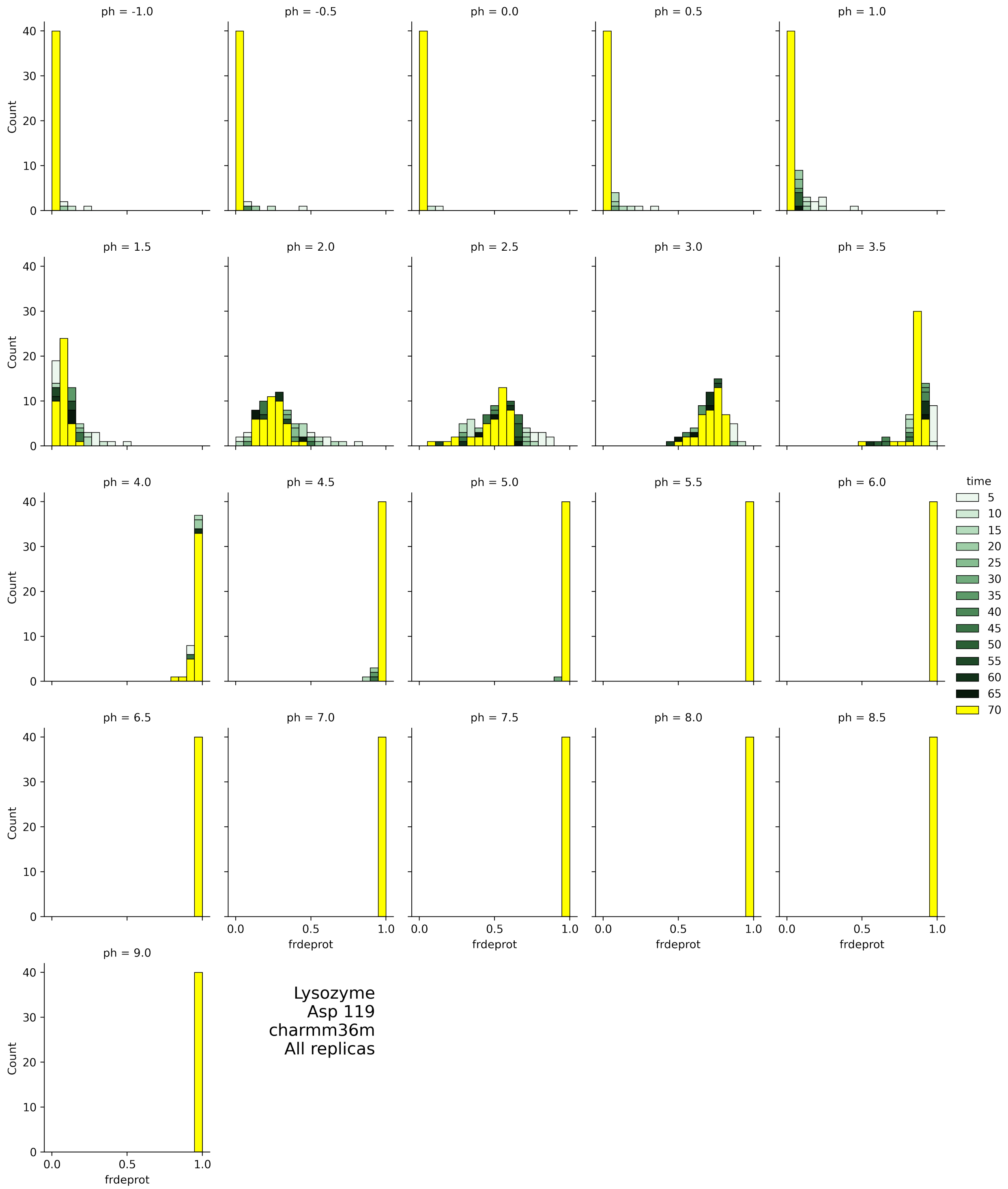

Figure S28: **Titration of Asp 119**: Histogram of the deprotonated fraction as a function of simulation duration (color coded, ns) over which it is computed, for all replicas. Computed for duration multiple of 5 ns.

2.4 Staphylococcal Nuclease

The following pH value were used for the titration of this protein: 1.0, 1.5, 2.0, 2.5, 3.0, 3.5, 4.0, 4.5, 5.0, 5.5, 6.0, 6.5, 7.0, 7.5, 8.0.

Table 5: Computational titration results for staphylococcal nuclease ΔPHS (40 replicas per pH point, 75 ns per replica) using the CHARMM36m force field. (x.xx - x.xx): Bootstrapped 95th percentile confidence intervals. **!**: spread-replica titration. Asp 19, Asp 21: the pH of the two chemical shift transition observed in NMR are reported in the Exp. column

Residue	HendHass pK_a	Exp.[5]
His 8	6.01 (5.99 - 6.04)	6.5
Glu 10	3.60 (3.55 - 3.64)	2.8
Asp 19	2.67 (4.49 - 5.12) **!**	2.2, 6.5
Asp 21	6.28 (3.44 - 3.89)**!**	3.0, 6.5
Asp 40	3.21 (3.16 - 3.26)	3.9
Glu 43	4.17 (4.16 - 4.19)	4.3
Glu 52	4.63 (4.62 - 4.65)	3.9
Glu 57	4.22 (4.21 - 4.24)	3.5
Glu 67	4.45 (4.42 - 4.48)	3.58
Glu 73	3.74 (3.71 - 3.77)	3.3
Glu 75	3.90 (3.82 - 3.98) **!**	3.3
Asp 77	1.45 (1.24 - 1.65) **!**	$<$2.2
Asp 83	0.60 (0.36 - 0.78) **!**	$<$2.2
Asp 95	3.09 (3.04 - 3.15)	2.2
Glu 101	4.17 (4.10 - 4.23)	3.8
His 121	4.89 (4.75 - 5.03) **!**	5.24
Glu 122	4.22 (4.14 - 4.29)	3.9
Glu 129	3.84 (3.79 - 3.89)	3.8
Glu 135	3.95 (3.91 - 3.99)	3.8
RMSE	0.53	

Table 6: Computational titration results for staphylococcal nuclease ΔPHS (40 replicas per pH point, 75 ns per replica) using the Amber99sb*-ILDN force field. (x.xx - x.xx): Bootstrapped 95th percentile confidence intervals. **!**: spread-replica titration. Asp 19, Asp 21: the pH of the two chemical shift transition observed in NMR are reported in the Exp. column

Residue	HendHass pK_a	Exp.[5]
His 8	5.50 (5.48 - 5.52)	6.5
Glu 10	3.12 (3.05 - 3.18)	2.8
Asp 19	2.37 (2.32 - 2.42)	2.2, 6.5
Asp 21	5.65 (5.53 - 5.78) **!**	3.0, 6.5
Asp 40	2.95 (2.91 - 3.00)	3.9
Glu 43	3.90 (3.88 - 3.92)	4.3
Glu 52	4.65 (4.63 - 4.67)	3.9
Glu 57	4.17 (4.15 - 4.18)	3.5
Glu 67	3.92 (3.90 - 3.93)	3.58
Glu 73	3.53 (3.51 - 3.55)	3.3
Glu 75	2.08 (1.95 - 2.20) **!**	3.3
Asp 77	1.82 (1.68 - 1.96) **!**	$<$2.2
Asp 83	0.07 (-0.21 - 0.28) **!**	$<$2.2
Asp 95	3.34 (3.33 - 3.37)	2.2
Glu 101	3.31 (3.25 - 3.37)	3.8
His 121	6.86 (6.77 - 6.95) **!**	5.24
Glu 122	2.72 (2.65 - 2.80)	3.9
Glu 129	3.97 (3.94 - 4.00)	3.8
Glu 135	2.98 (2.92 - 3.05)	3.8
RMSE	0.83	

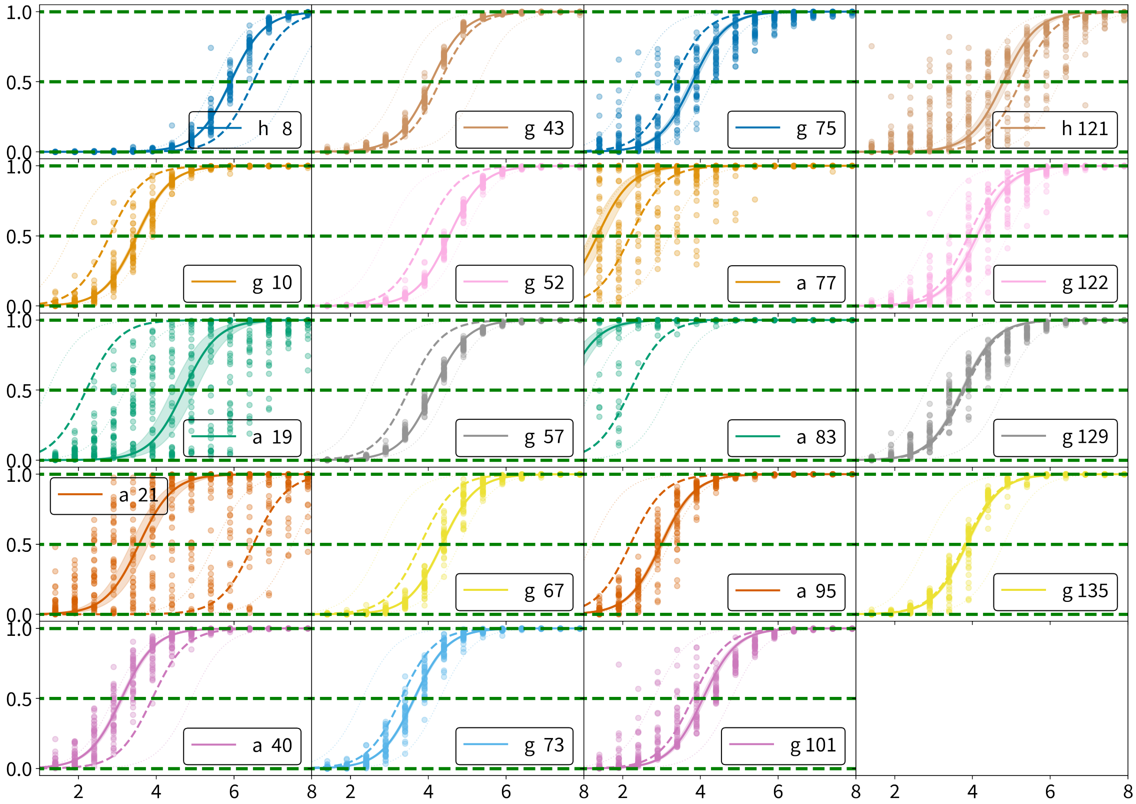

Figure S29: Individual computational titration curves for all residues in Staph. Nuclease Δ PHS, with all replicas shown (circles), for CHARMM36m. Labelled as first letter of amino acid type and sequence number of the residue. Solid line: fitted Henderson-Hasselbalch curve to constant pH data. Dashed line: Henderson-Hasselbalch curve from NMR-determined pKa

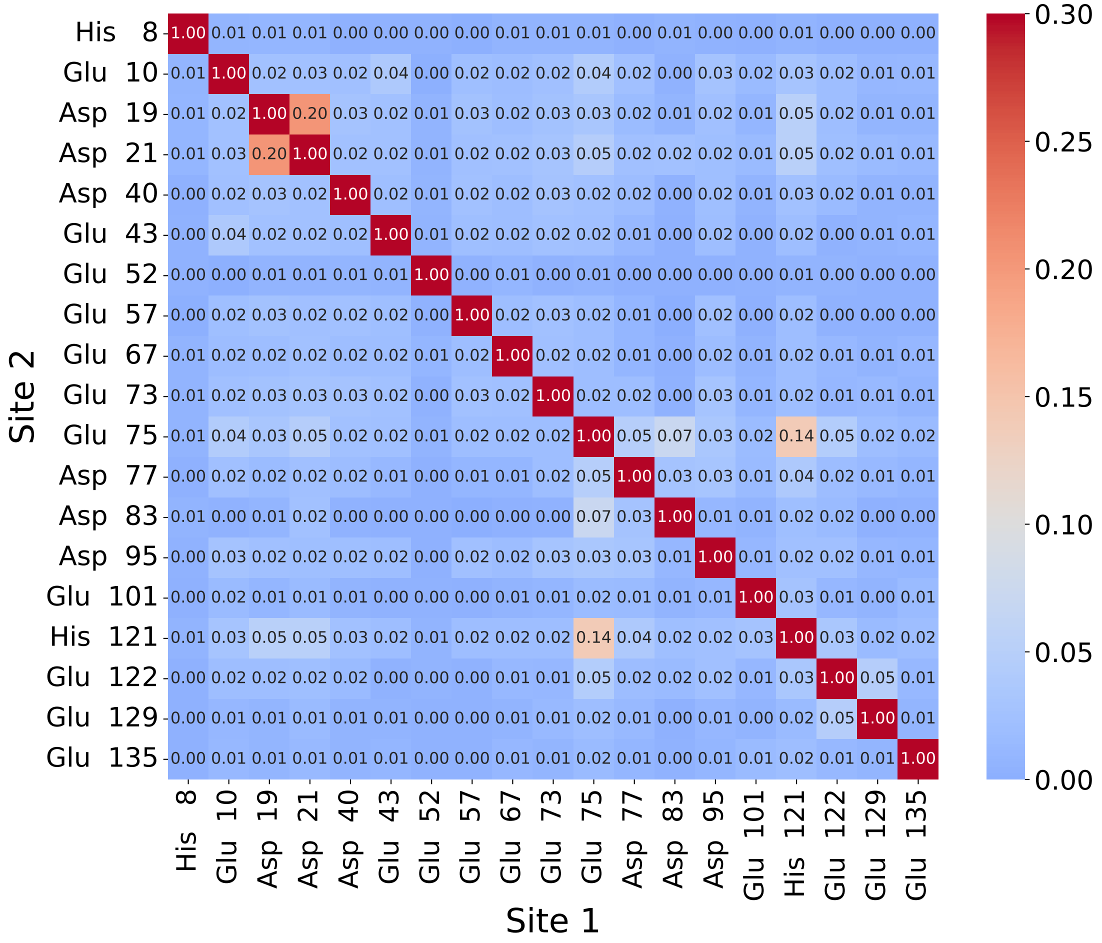

Figure S30: Matrix of the mean Normalized Mutual Information for each pairs of residue (using the CHARMM36m force field), at the pH point where is is maximum for that pair. Non-diagonal red squares correspond to a positive criteria (NMI > 0.1) under our criteria.

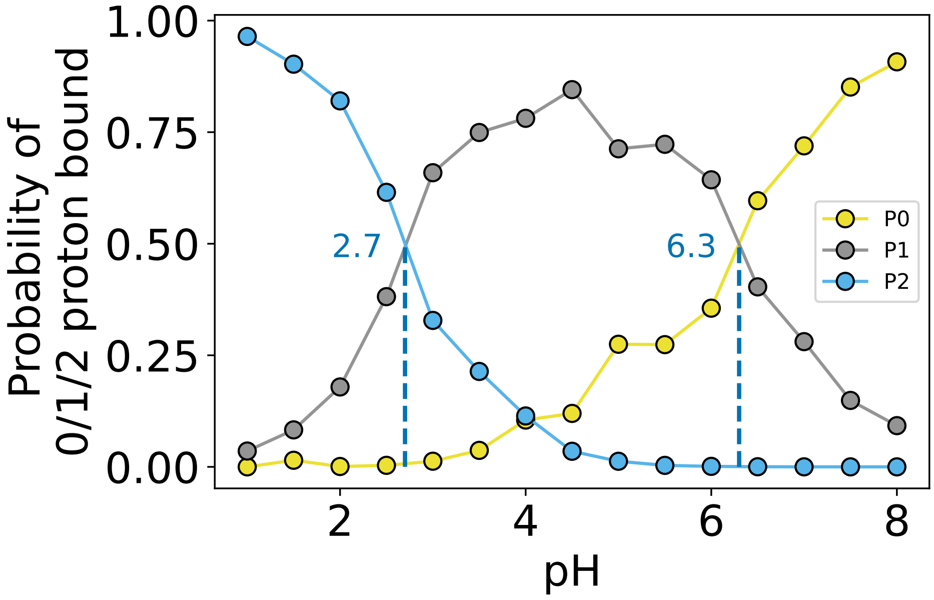

Figure S31: **Titration of Asp 19 – Asp 21** using the CHARMM36m force field. The probability of binding 0, 1 or 2 protons to the pair of residues is shown. These quantities are average over all replicas. The pH point at which the probability of binding 0 and 2 protons, respectively, crosses the 0.5 threshold is shown as vertical lines, and corresponds to the macroscopic pK_a values, yielding the same results as the macroscopic titration curve model found in the main text. This alternative method is described in detail in Yue et al.[6]

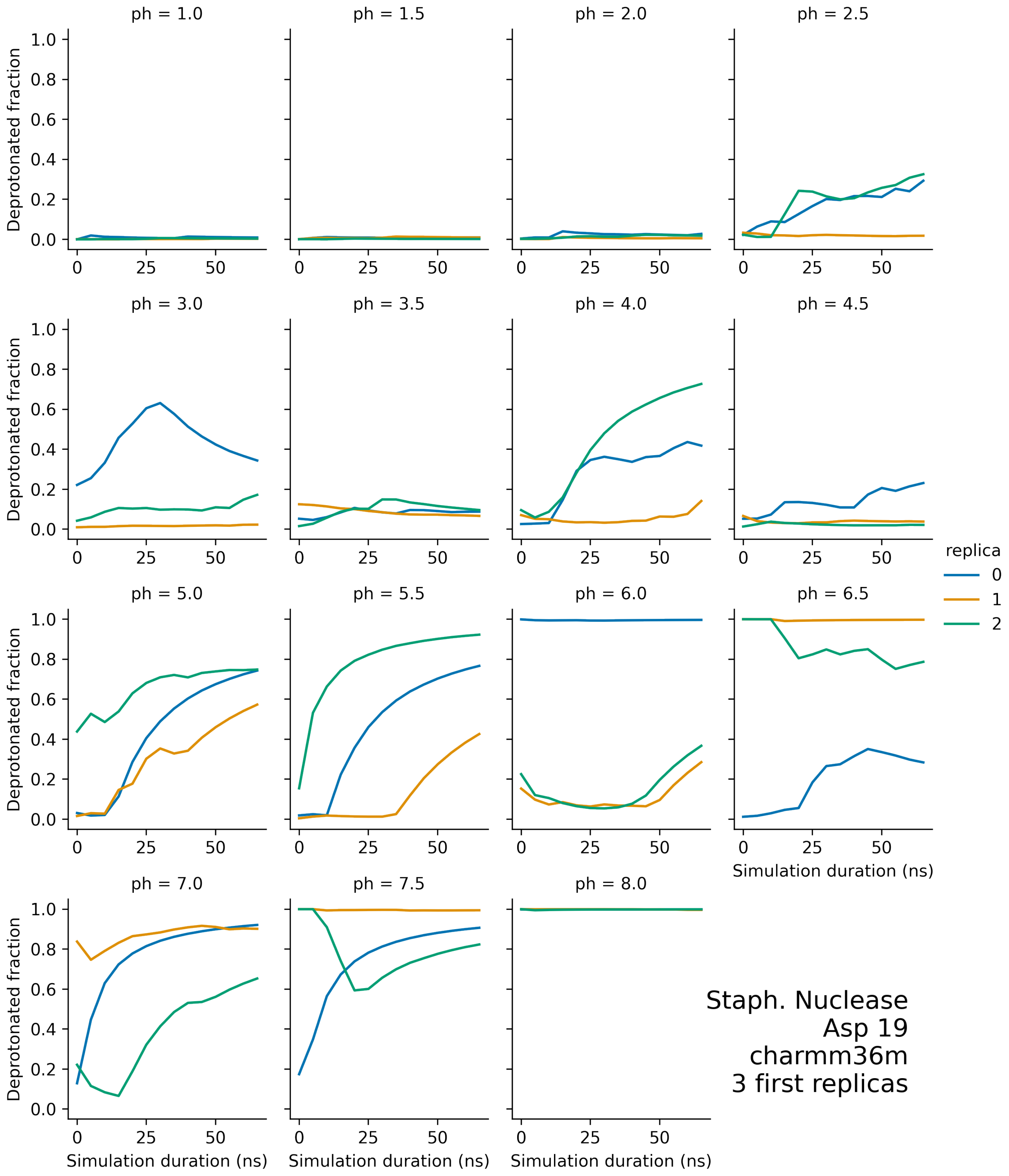

Figure S32: **Titration of Asp 19**: Deprotonated fraction as a function of simulation duration (ns) over which it is computed, for the three first replicas. Computed for duration multiple of 5 ns.

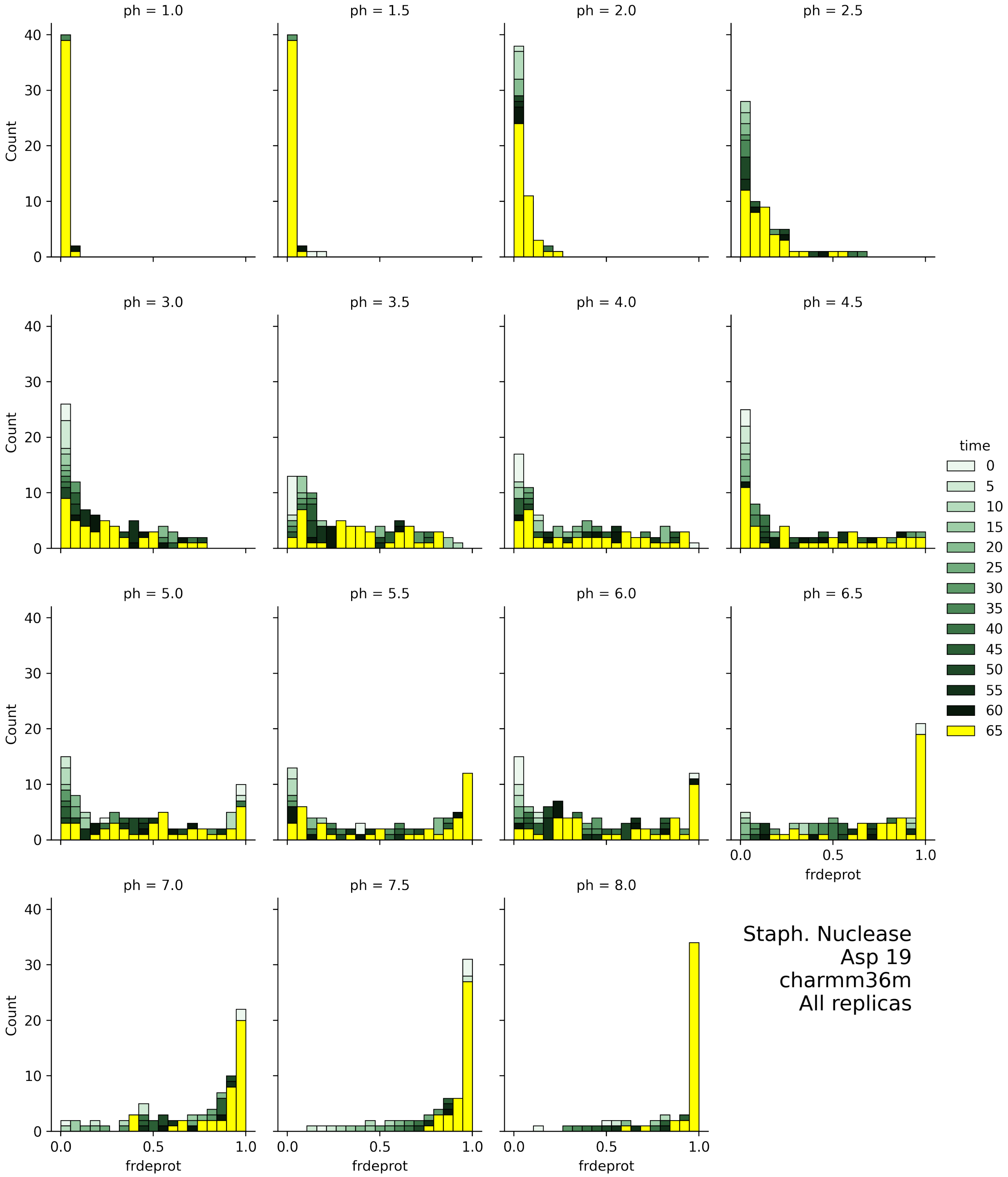

Figure S33: **Titration of Asp 19**: Histogram of the deprotonated fraction as a function of simulation duration (color coded, ns) over which it is computed, for all replicas. Computed for duration multiple of 5 ns.

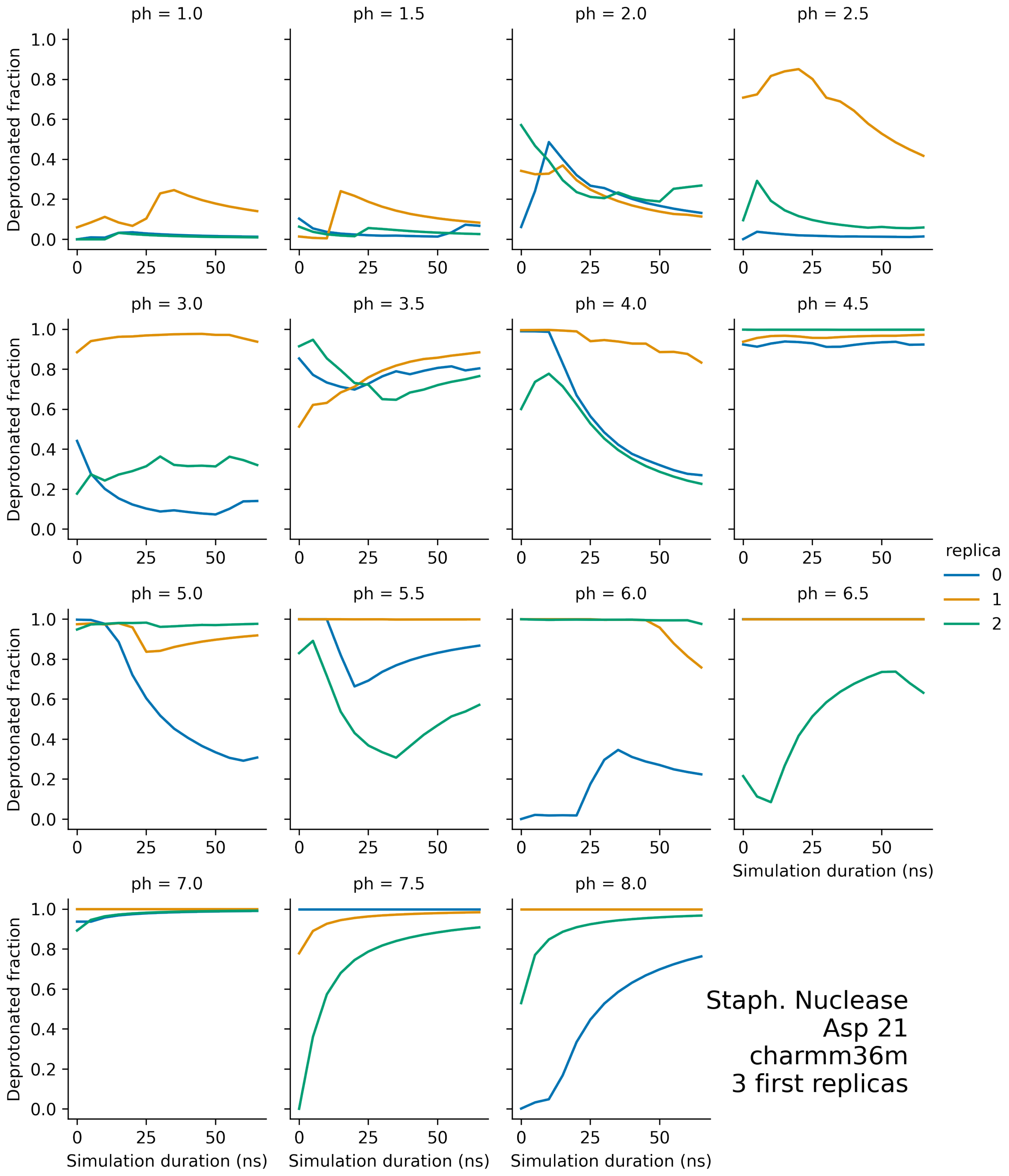

Figure S34: **Titration of Asp 21**: Deprotonated fraction as a function of simulation duration (ns) over which it is computed, for the three first replicas. Computed for duration multiple of 5 ns.

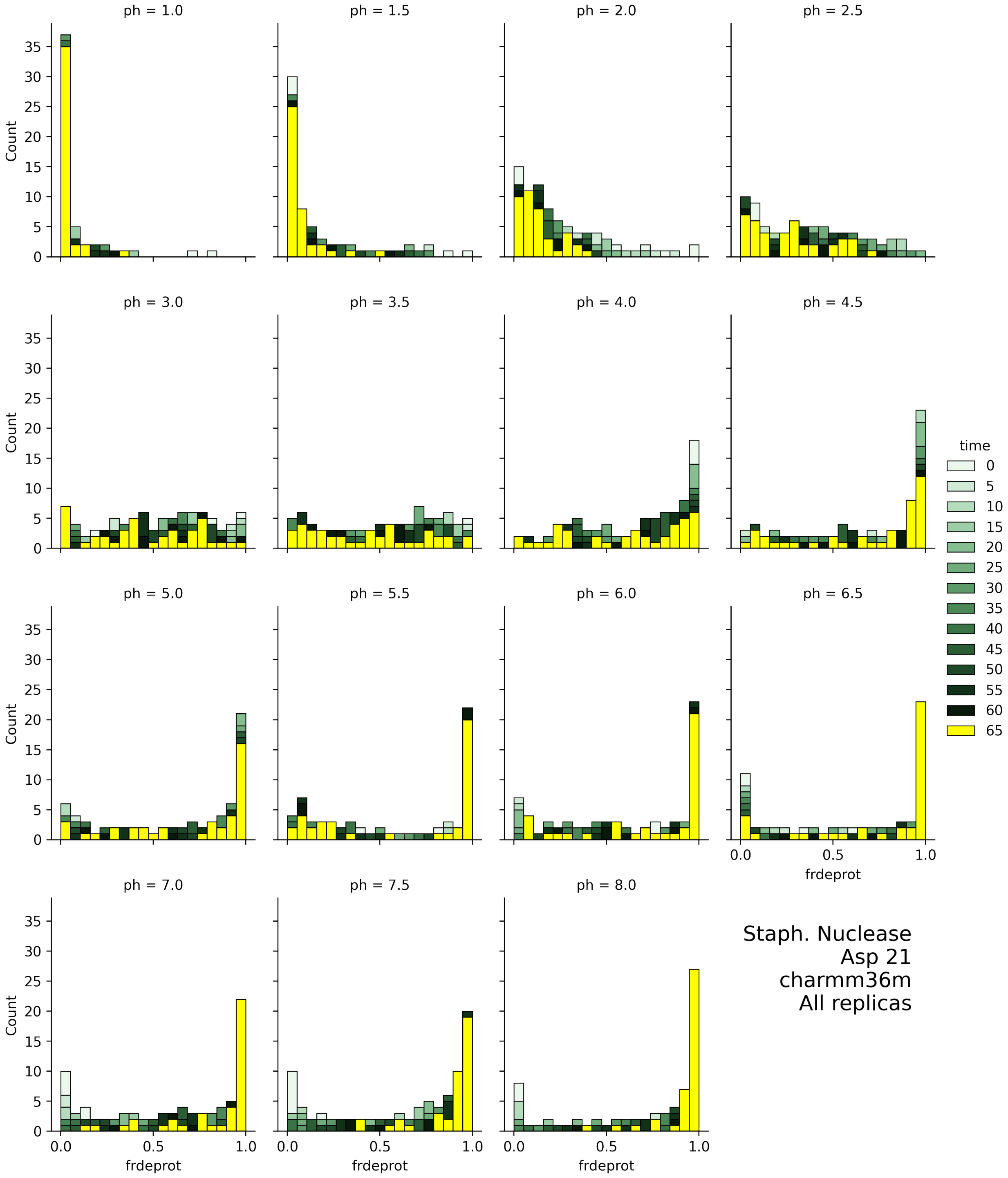

Figure S35: **Titration of Asp 21**: Histogram of the deprotonated fraction as a function of simulation duration (color coded, ns) over which it is computed, for all replicas. Computed for duration multiple of 5 ns.

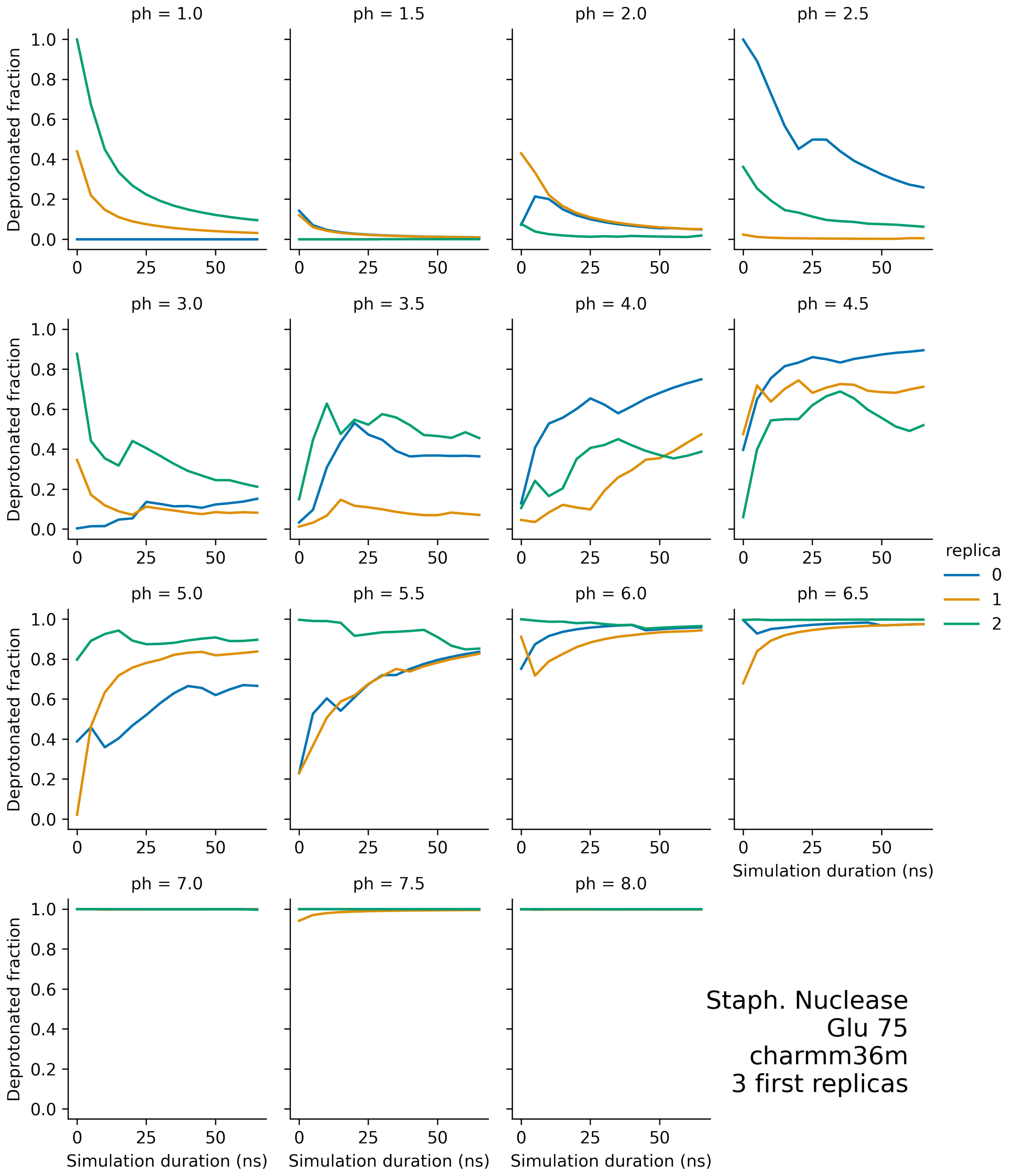

Figure S36: **Titration of Glu 75**: Deprotonated fraction as a function of simulation duration (ns) over which it is computed, for the three first replicas. Computed for duration multiple of 5 ns.

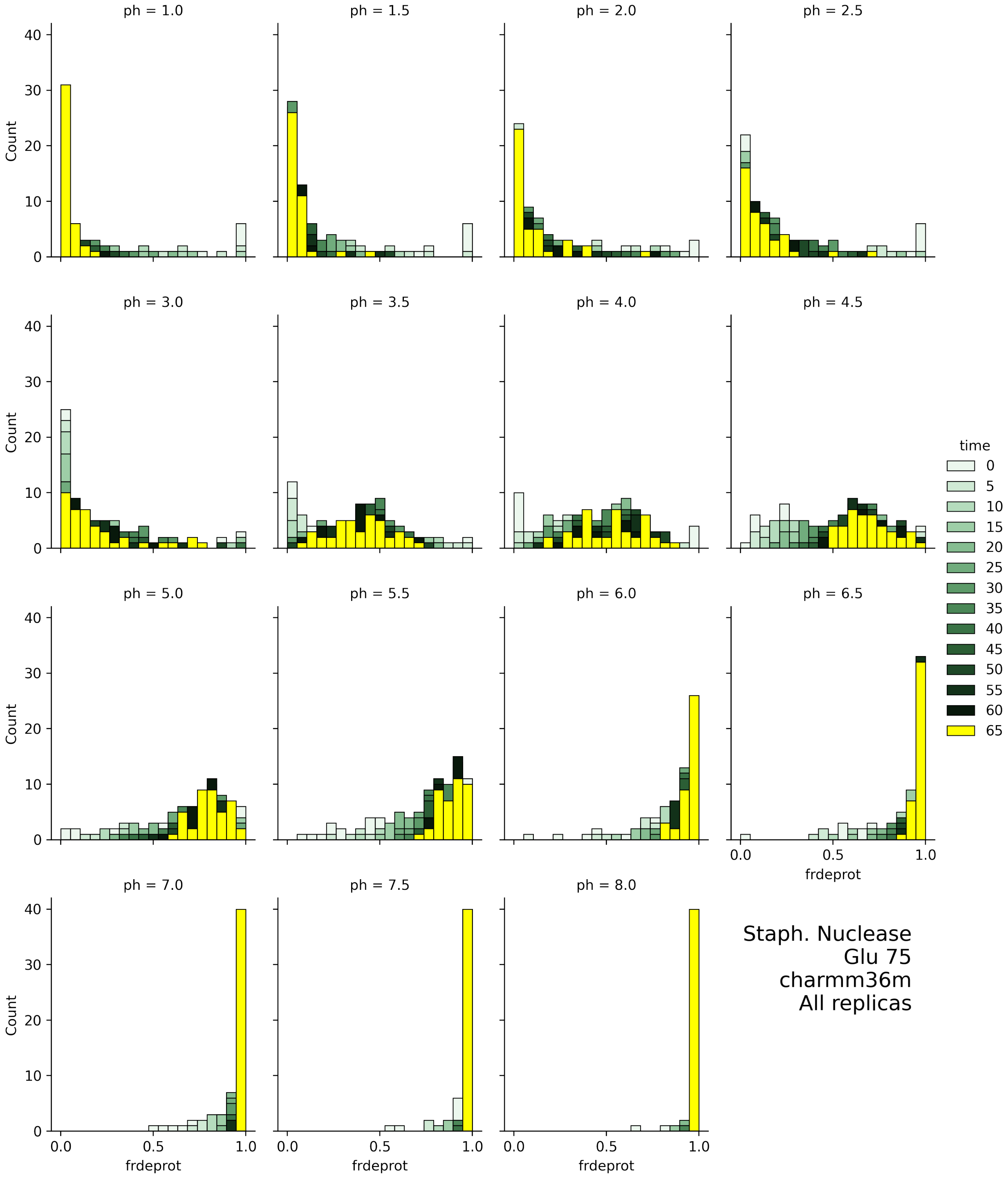

Figure S37: **Titration of Glu 75**: Histogram of the deprotonated fraction as a function of simulation duration (color coded, ns) over which it is computed, for all replicas. Computed for duration multiple of 5 ns.

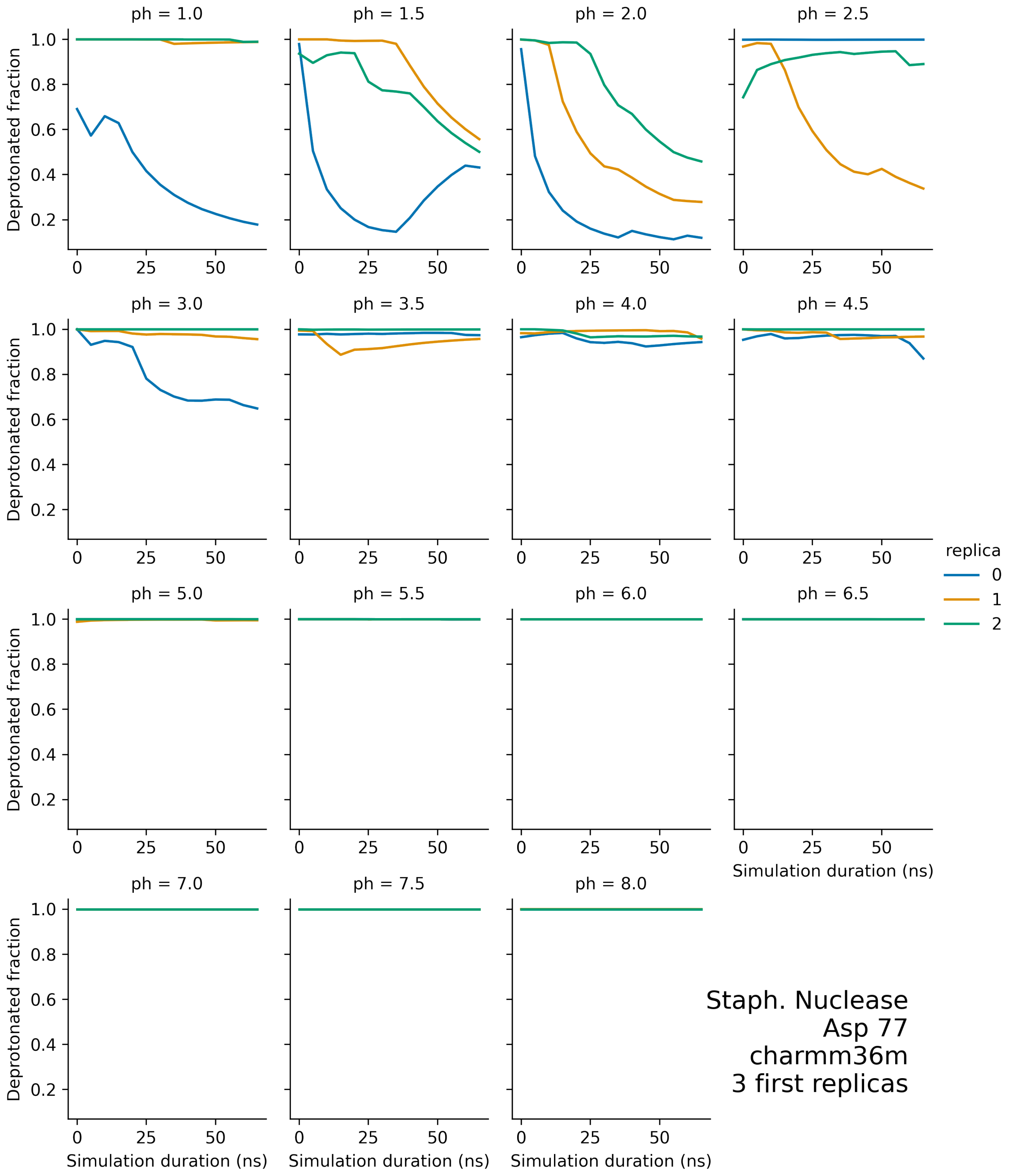

Figure S38: **Titration of Asp 77**: Deprotonated fraction as a function of simulation duration (ns) over which it is computed, for the three first replicas. Computed for duration multiple of 5 ns.

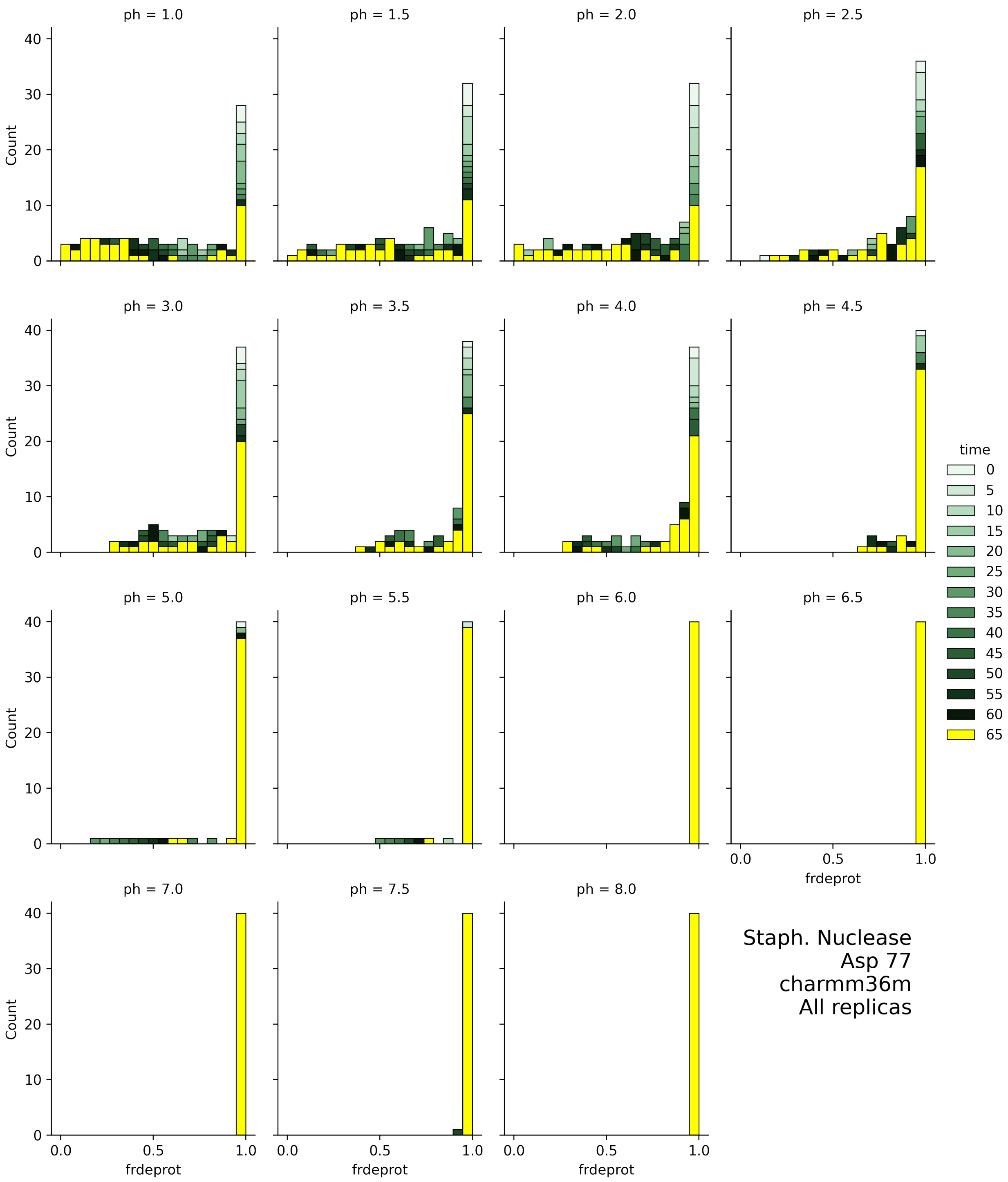

Figure S39: **Titration of Asp 77**: Histogram of the deprotonated fraction as a function of simulation duration (color coded, ns) over which it is computed, for all replicas. Computed for duration multiple of 5 ns.

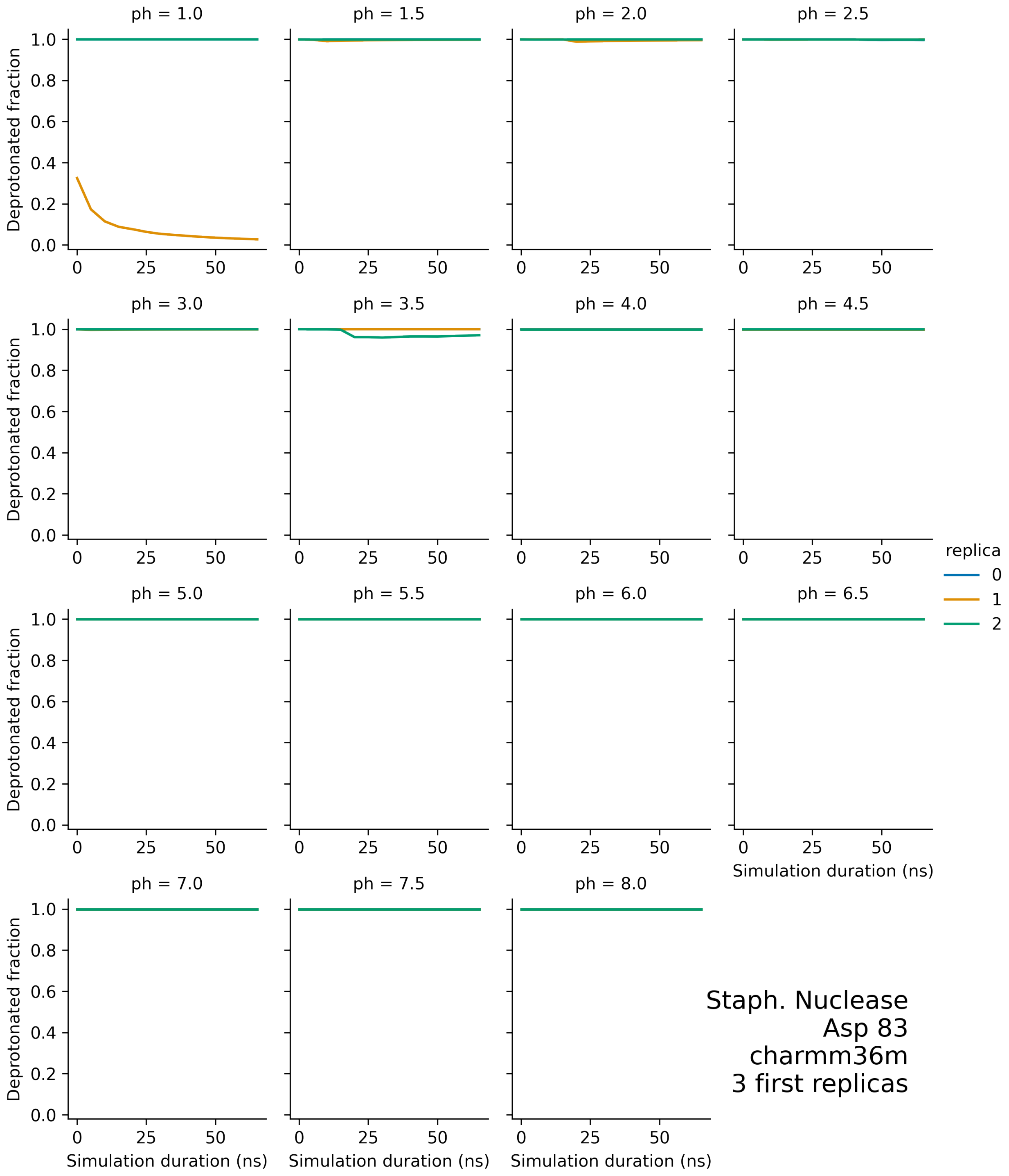

Figure S40: **Titration of Asp 83**: Deprotonated fraction as a function of simulation duration (ns) over which it is computed, for the three first replicas. Computed for duration multiple of 5 ns.

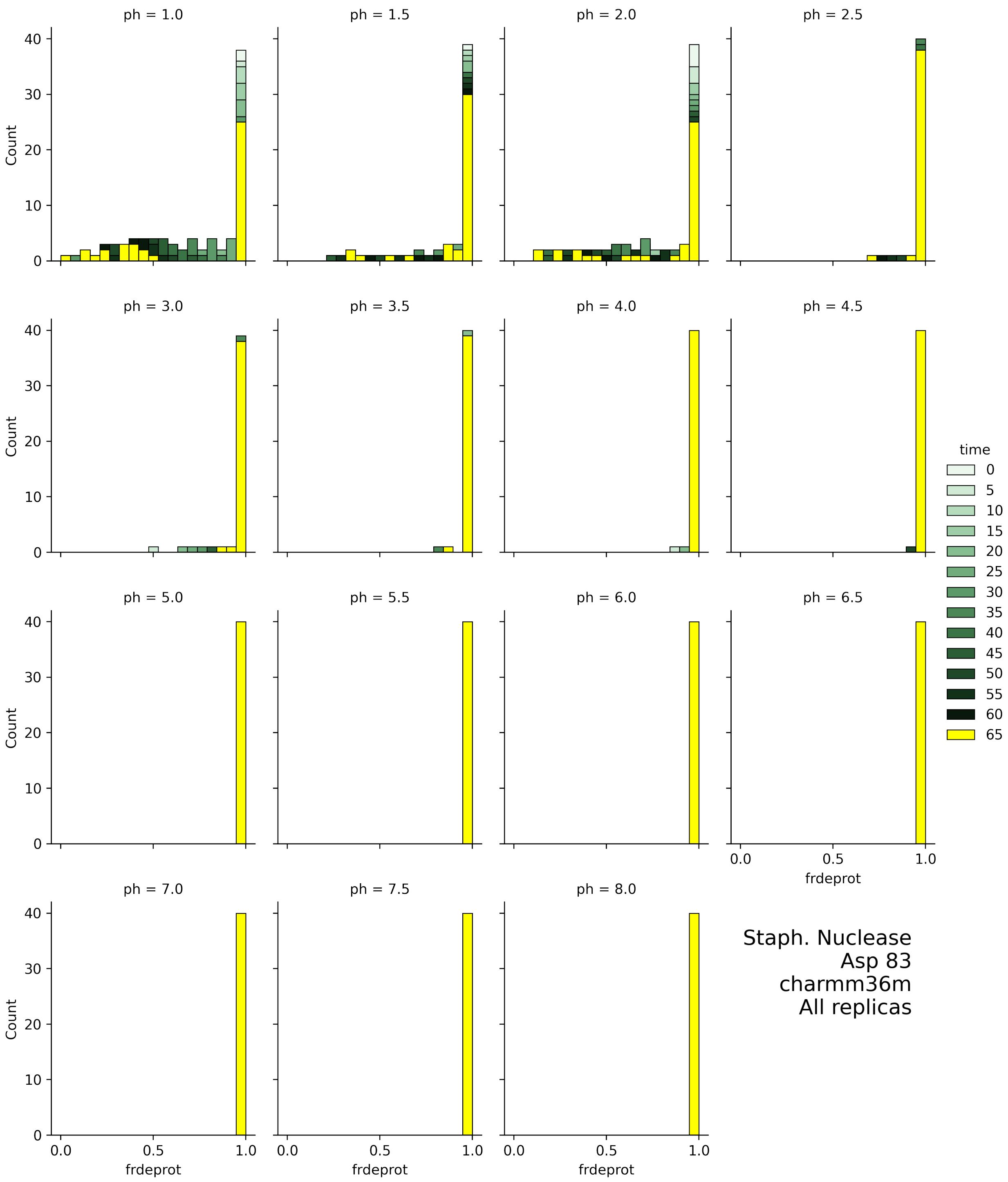

Figure S41: **Titration of Asp 83**: Histogram of the deprotonated fraction as a function of simulation duration (color coded, ns) over which it is computed, for all replicas. Computed for duration multiple of 5 ns.

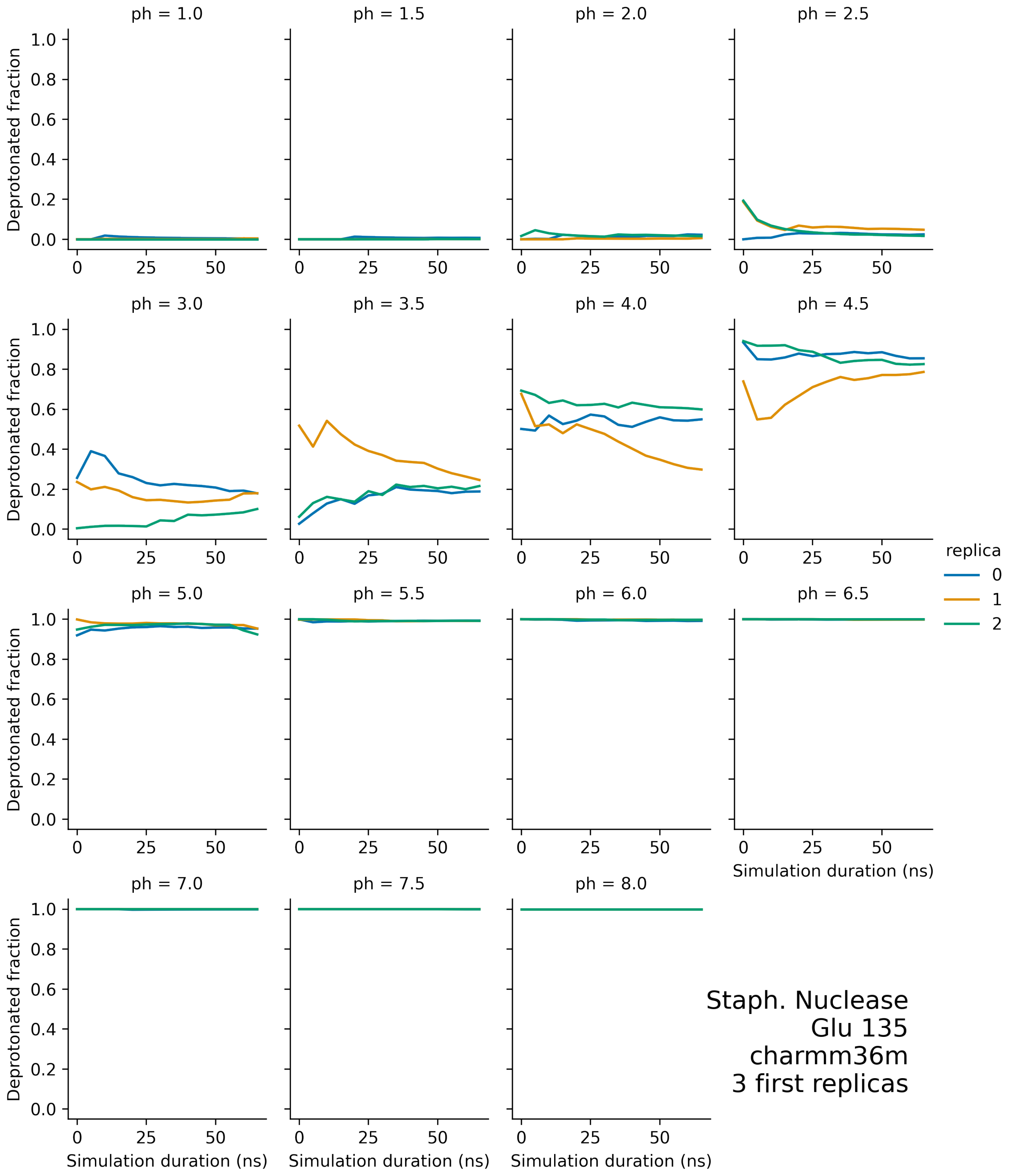

Figure S42: **Titration of Glu 135**: Deprotonated fraction as a function of simulation duration (ns) over which it is computed, for the three first replicas. Computed for duration multiple of 5 ns.

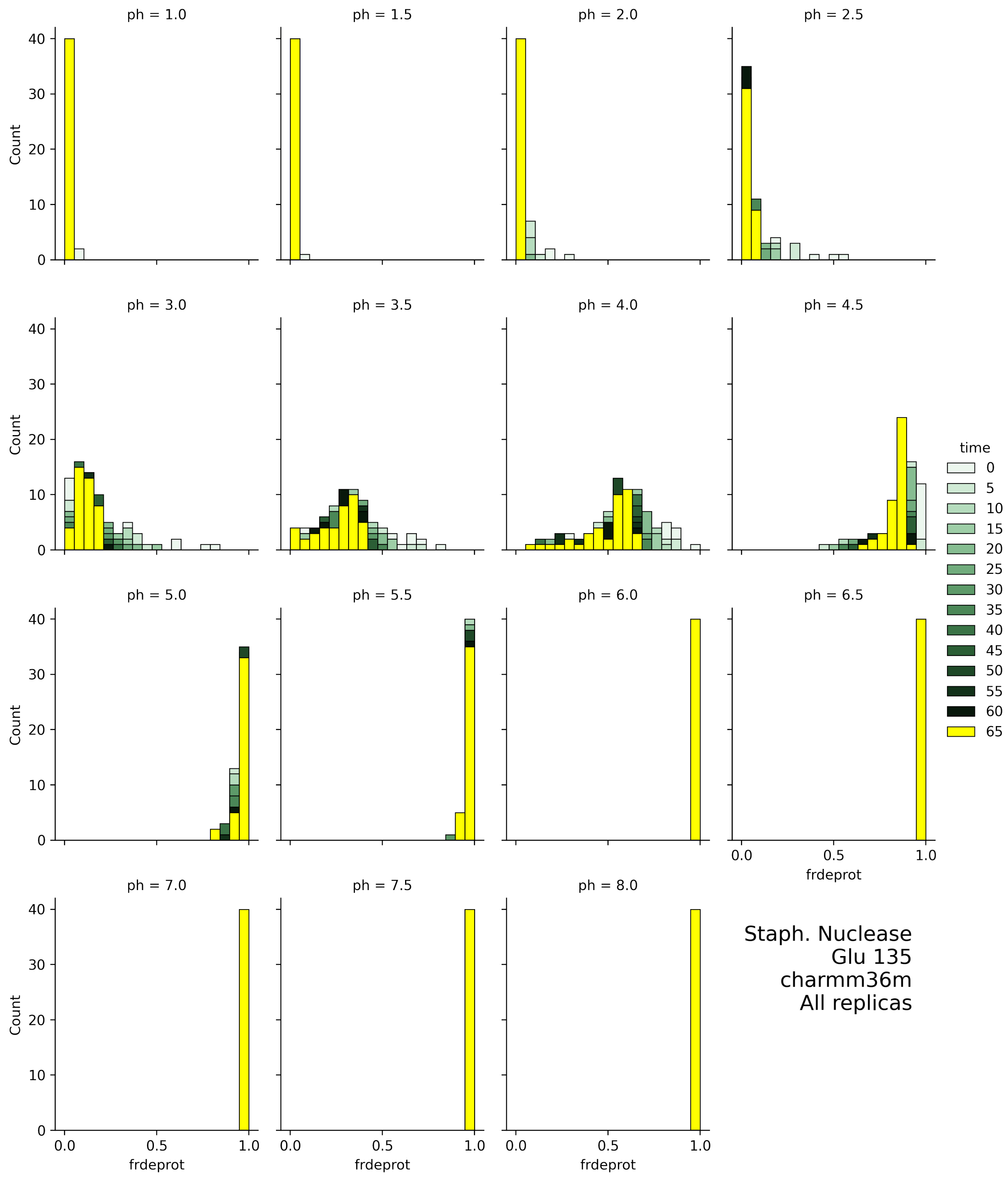

Figure S43: **Titration of Glu 135**: Histogram of the deprotonated fraction as a function of simulation duration (color coded, ns) over which it is computed, for all replicas. Computed for duration multiple of 5 ns.

3 FMA Projections

3.1 Cardiotoxin Asp 59

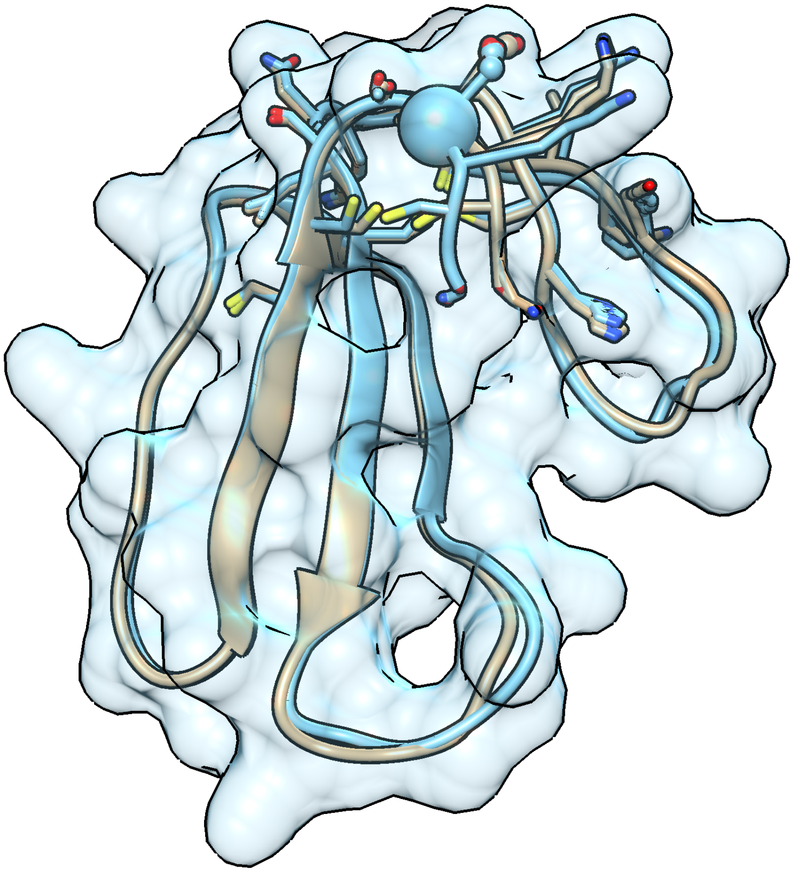

Figure S44: Low (tan) and high (blue) FMA value structure for Asp 59 in Cardiotoxin. Solvent-excluded surface for the high FMA value structure shown, with sphere locating the Asp 59 residue.

3.2 Staphylococcal Nuclease

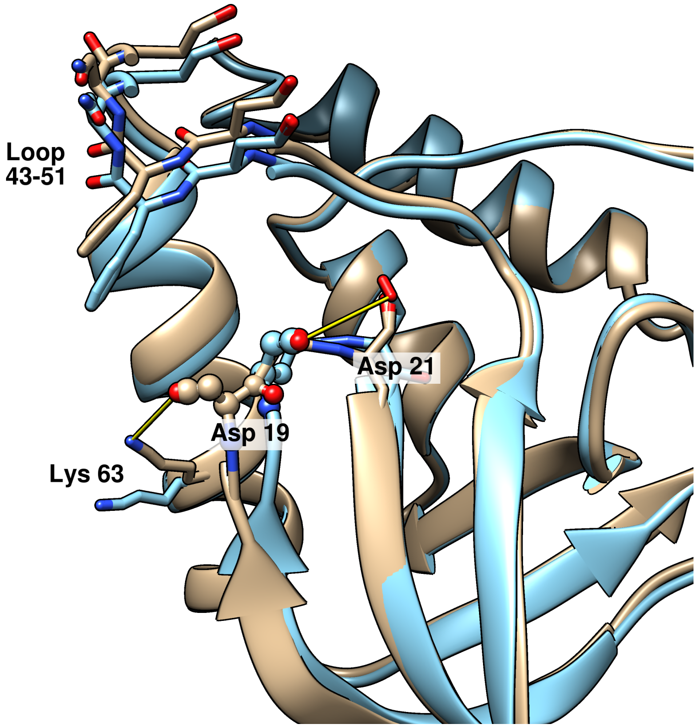

Figure S45: Low (tan) and high (blue) FMA value structure for Asp 19 in SNase. Relevant interaction of Asp 19 depicted with yellow pseudobonds.

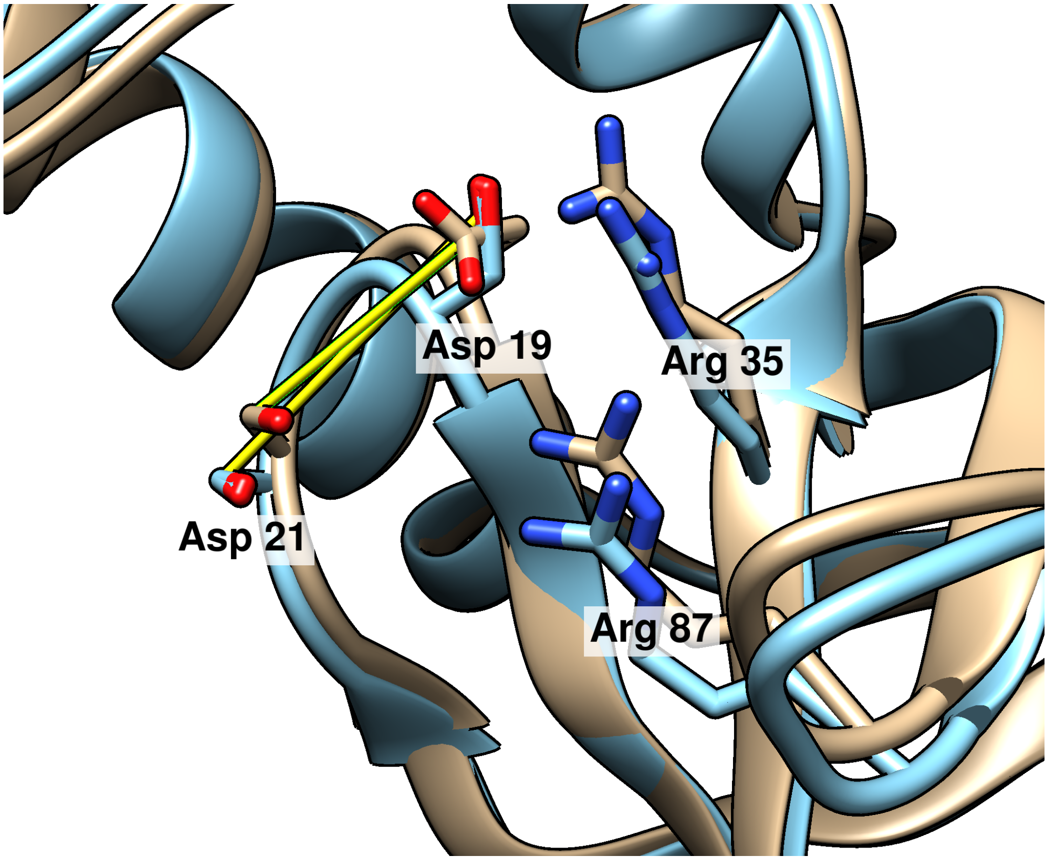

Figure S46: Low (tan) and high (blue) FMA value structure for Asp 21 in SNase. Relevant interaction of Asp 19 depicted with yellow pseudobonds.

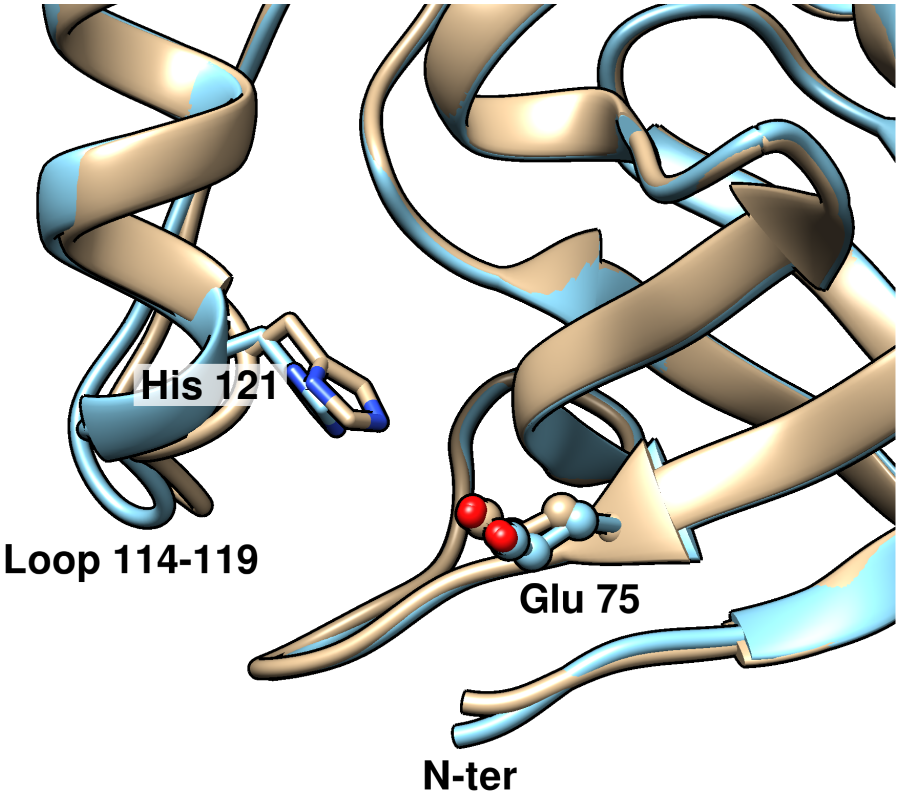

Figure S47: Low (tan) and high (blue) FMA value structure for Glu 75 in SNase.

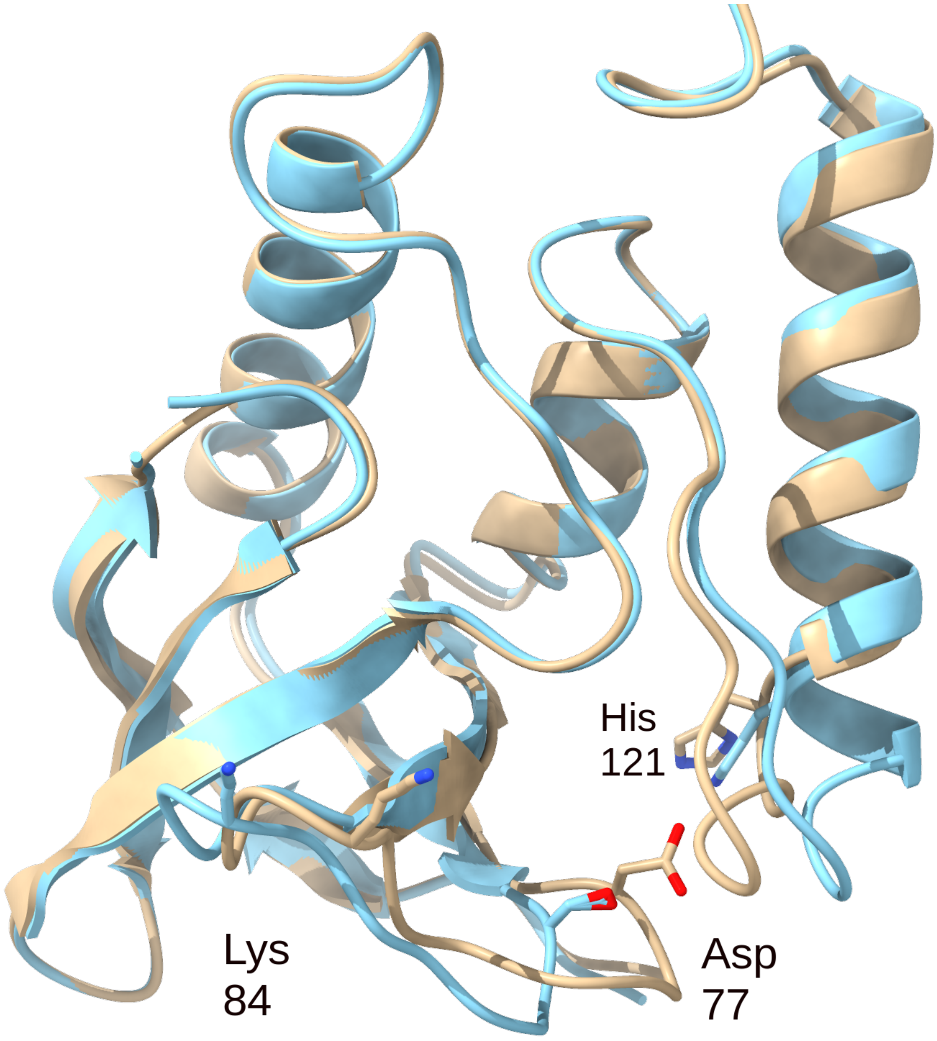

Figure S48: Low (tan) and high (blue) FMA value structure for Asp 77 in SNase. Apart from Asp 77, the other labels are placed to help orientation in the structure, and not to imply interactions with Asp 77. The two fraying loops, differentiating the high and low FMA structures, and presumably involved in early stage of acid-mediated unfolding, run from Glu 75 to Arg 87, and from Val 111 to Glu 122

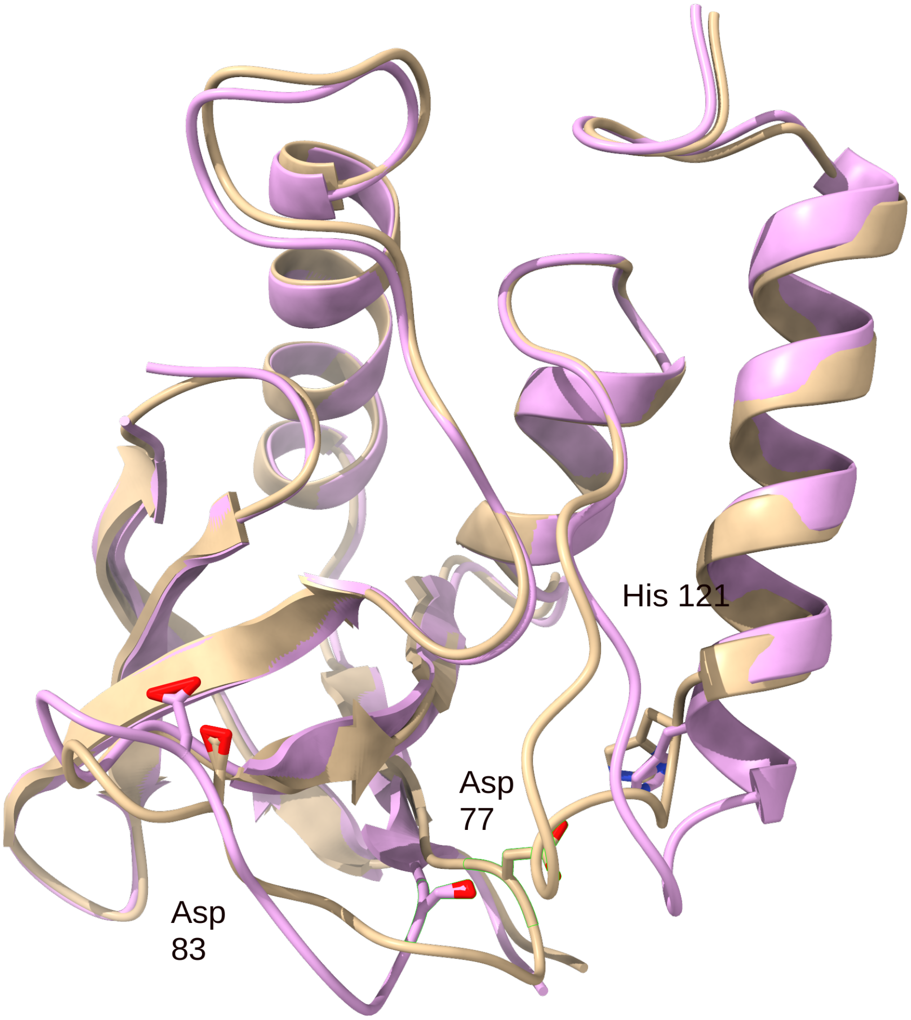

Figure S49: Low (tan) and high (blue) FMA value structure for Asp 83 in SNase. Apart from Asp 83, the other labels are placed to help orientation in the structure, and not to imply interactions with Asp 83. The two fraying loops are similar to those in the figure above concerning Asp 77.

4 Simulations Setup Details

4.1 NVT Simulations

The constant pH simulations in this work were performed in the NVT ensemble, as our GPU-accelerated FMM codes does not yet support pressure coupling. System solvation

was performed using the GROMACS `solvate` tool, which yields solvent density slightly lower in our subsequent constant pH NVT simulation than an equivalent NPT simulations (around 1004 versus 1027 kg/m^3). To correct the density, the user may choose to run a pre-equilibration NPT simulation (without constant pH) prior to the NVT constant pH MD simulations. The subsequent constant pH simulations will requires a different set of calibration data, which can be enabled by setting the `CPH_PRESSUREEQUIL_FIT` define in the `.mdp` file – see Listing 1. We suspect that this is necessary due to the slightly different dielectric permittivity ε of water owing to the increased density, which affects the residue – buffer site contribution to $\partial\mathcal{H}/\partial\lambda$.

However, we found empirically that such pre-equilibration did not affect pK_a values for our test system lysozyme (Figure S50), a globular protein. We therefore did not use this equilibration procedure in this work. We speculate that, in contrast, this result would not necessarily hold for non-homogeneous systems such as non-globular proteins or lipid membranes.

Future development of our FMM implementation will provide a definitive solution to this issue by incorporating pressure coupling.

```
define                          =   -DCPH_PRESSUREEQUIL_FIT
```

Listing 1: Enabling the set of calibration data for NPT-equilibrated box

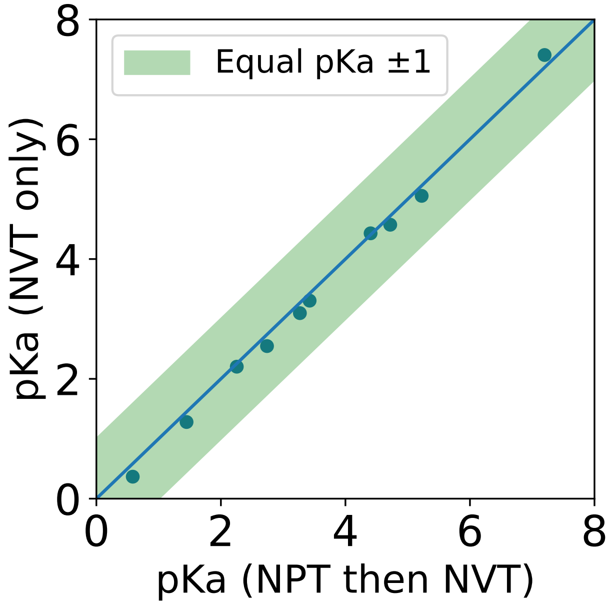

Figure S50: pK_a values for constant pH titration of HEWL (CHARMM36m) where all simulations took place in the NVT ensemble (y-axis) compared to a protocol with a non-constant pH NPT equilibration preceding the titration itself (also in the NVT ensemble). Maximum pK_a deviation is 0.2, Pearson correlation coefficient is 0.99

5 Alternative references compounds for calibration

In theory, any reference compound containing the residue of interest can be used for calibration in λ-dynamics CPH MD, provided the corresponding pK_a value is then used as reference pK_a for the residue. As a result, different CPH codes have been using different reference compounds, in particular AAXAA pentapeptides.[7–9] To assess the putative influence of using those pentapeptides, we have titratated them while using our calibration data based on blocked residues (same protocol as for single residue titrations).

As can be seen, the resulting ΔpK_a are minimal, especially compared to the overall pK_a RMSE (around 0.6 - 0.8 depending on protein and CPH protocols) seen on protein benchmarks, indicating that the choice of reference compound is not the limiting factor in CPH accuracy, at least in the present state of the method.

Table 7: ΔpK_a to reference pK_a for AAXAA pentapeptides, simulated using CHARMM36m. The 95% confidence interval is obtained by boostrapping (see Methods in main text).

Compound	ΔpK_a (CPH)	95% Confidence Interval
Ala-Ala-Glu-Ala-Ala	-0.03	±0.01
Ala-Ala-Asp-Ala-Ala	-0.04	±0.03
Ala-Ala-His-Ala-Ala	-0.02	±0.02

6 Double well potential definition

The double well potential is implemented in `SplineBias.cpp` in the source code.

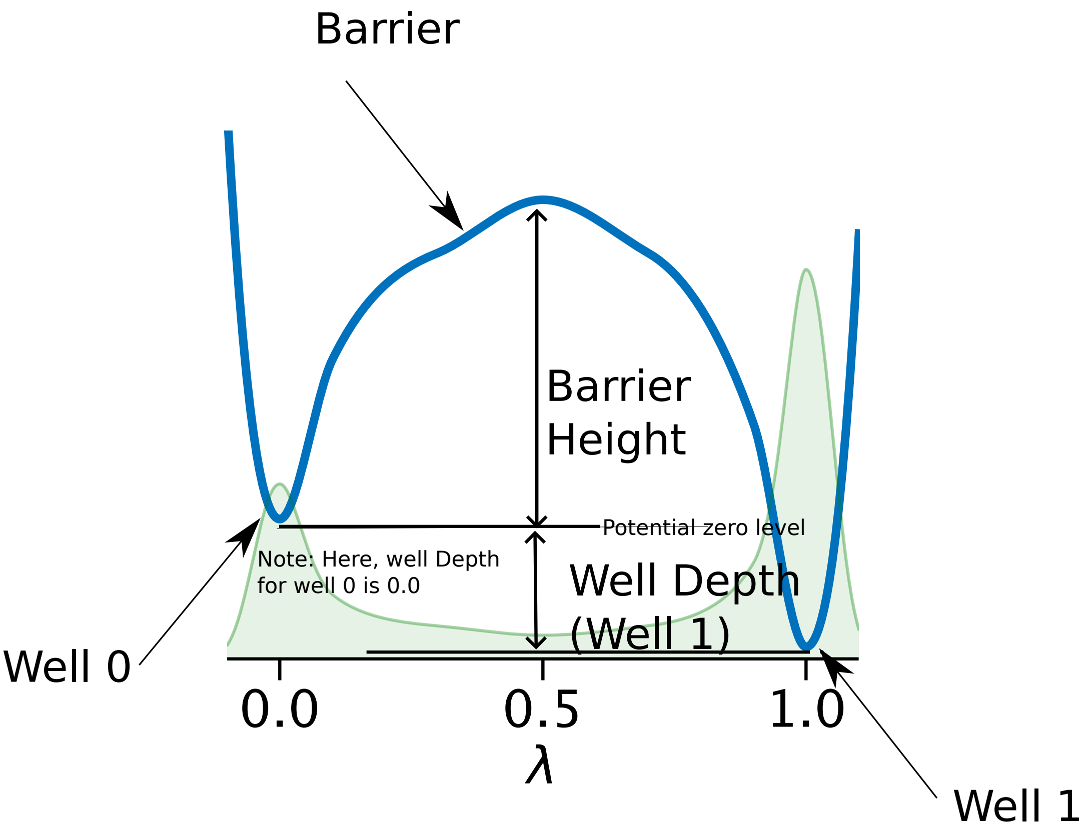

Figure S51: Double well potential with label for the various relevant height/depth and important points.

The double well potential is composed of a main spline section, and exponential wall around the 0-1 interval. The spline are Hermite cubic spline, which are C1 continuous, and have specified derivative at the control points. The spline and derivative are con-

structed such that there is a monotonous decrease from outer wall to well bottom, and a monotonous increase from well bottom to top of the barrier, given the following conditions are satisfied: barrier height zero or positive, left and right depth zero or negative, left and right well position within -0.20 to 0.20 and 0.80 to 1.20 respectively (could possibly hold outside those condition but not verified). The control variables (well positions, well depth, barrier height) are fully independent. e.g. change in well offset does not affect the effective barrier height, apart of course from increasing the overall well+barrier transition barrier.

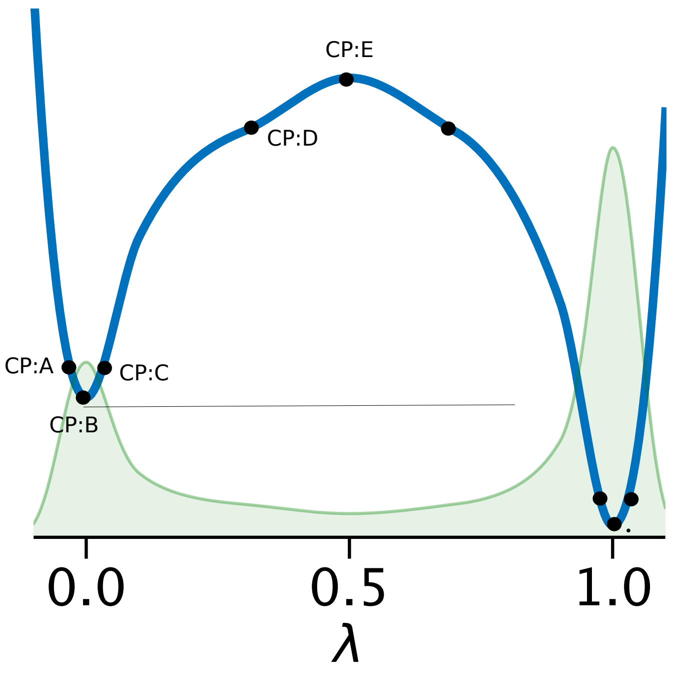

Figure S52: Spline control point for the bias (CP: Control point)

The shape of the double well potential will be described for the left (well 0) side, as the other wide is defined in the same way around the point $\lambda = 1$. Important charactetistics are: An exponential wall is present from $-\infty$ to control point A, with expression $\exp(-R * \lambda) - 1$. By default, R is 20 kj/mol. Control point A is the beginning of spline

toward control point B, at which the potential is enforced to have derivative 0 (thus, resulting force is 0). The position of the well minima, which is control point B, is set in both λ and energy (y-axis) by the constant pH code. For instance, the well adjustment algorithm might adjust its position near 0 to ensure the average λ in this well is indeed 0.0. The position on the energy (y-axis) is either 0, or a (negative) well depth, to realize the desired depth difference between the two well (which is how the pH-linked free energy of (de)protonation is set). Control point A and C are positioned relative to control point B. Control point E is the midpoint, whose height corresponds to the barrier height. The derivative of the potential in E is enforced to be zero (zero force). Control point D is necessary to ensure monotonicity of increase between B, C and E. In its absence, a low barrier height can lead to overshot above the barrier height between C and E. The position of D (and its height relative to E) on the λ axis can be adjusted to set the flatness of the potential barrier — we have chosen a rather round barrier, but a virtually flat barrier is also possible.

For the exact function, derivative and position of this highly configurable bias, we refer to `SplineBias.cpp` in the source code.

References

(1) Dobrev, P.; Vemulapalli, S. P. B.; Nath, N.; Griesinger, C.; Groenhof, G.; Grubmüller, H. Probing the Accuracy of Explicit Solvent Constant pH Molecular Dynamics Simulations for Peptides. *J. Chem. Theory Comput.* **2020**, *16*, 2561–2569.

(2) Chiang, C.-M.; Chien, K.-Y.; Lin, H.-j.; Lin, J.-F.; Yeh, H.-C.; Ho, P.-l.; Wu, W.-g. Conformational change and inactivation of membrane phospholipid-related activity of cardiotoxin V from Taiwan cobra venom at acidic pH. *Biochemistry* **1996**, *35*, 9167–9176.

(3) Chiang, C.-M.; Chang, S.-L.; Lin, H.-j.; Wu, W.-g. The role of acidic amino acid residues in the structural stability of snake cardiotoxins. *Biochemistry* **1996**, *35*, 9177–9186.

(4) Webb, H.; Tynan-Connolly, B. M.; Lee, G. M.; Farrell, D.; O'Meara, F.; Søndergaard, C. R.; Teilum, K.; Hewage, C.; McIntosh, L. P.; Nielsen, J. E. Remeasuring HEWL pK_a values by NMR spectroscopy: Methods, analysis, accuracy, and implications for theoretical pK_a calculations. *Proteins Struct. Funct. Bioinf.* **2011**, *79*, 685–702.

(5) Castañeda, C. A.; Fitch, C. A.; Majumdar, A.; Khangulov, V.; Schlessman, J. L.; García-Moreno, B. E. Molecular determinants of the pK_a values of Asp and Glu residues in staphylococcal nuclease. *Proteins Struct. Funct. Bioinf.* **2009**, *77*, 570–588.

(6) Yue, Z.; Chen, W.; Zgurskaya, H. I.; Shen, J. Constant pH molecular dynamics reveals how proton release drives the conformational transition of a transmembrane efflux pump. *J. Chem. Theory Comput.* **2017**, *13*, 6405–6414.

(7) Huang, Y.; Chen, W.; Wallace, J. A.; Shen, J. All-atom continuous constant pH molecular dynamics with particle mesh Ewald and titratable water. *J. Chem. Theory Comput.* **2016**, *12*, 5411–5421.

(8) Harris, J. A.; Liu, R.; Martins de Oliveira, V.; Vázquez-Montelongo, E. A.; Henderson, J. A.; Shen, J. GPU-Accelerated All-Atom Particle-Mesh Ewald Continuous Constant pH Molecular Dynamics in Amber. *J. Chem. Theory Comput.* **2022**, *18*, 7510–7527.

(9) Pace, C. N.; Grimsley, G. R.; Scholtz, J. M. Protein ionizable groups: pK values and their contribution to protein stability and solubility. *J. Biol. Chem.* **2009**, *284*, 13285–13289.
